# Supplementary material for: Accuracy of efficient data methods to determine the incidence of hospital-acquired thrombosis and major bleeding in medical and surgical inpatients: a multicentre observational cohort study in four UK hospitals
Source: BMJ Open. 2023 Feb 6;13(2):e069244. doi: 10.1136/bmjopen-2022-069244 (PMC9906300; doi:10.1136/bmjopen-2022-069244)
Supplement: Supplementary data [file bmjopen-2022-069244supp001.pdf]

Supplementary material: Evaluating routine data sources to determine the incidence of hospital acquired thrombosis and major bleeding in medical and surgical inpatients.

Figure 1: Examples of electronic and paper documentation aids for RAM completion

Site 1- Structured note completed through Electronic Health Record and designed to aid VTE risk assessment at the point of hospital admission, using the Department of Health RAM (Salford Royal NHS Foundation Trust).

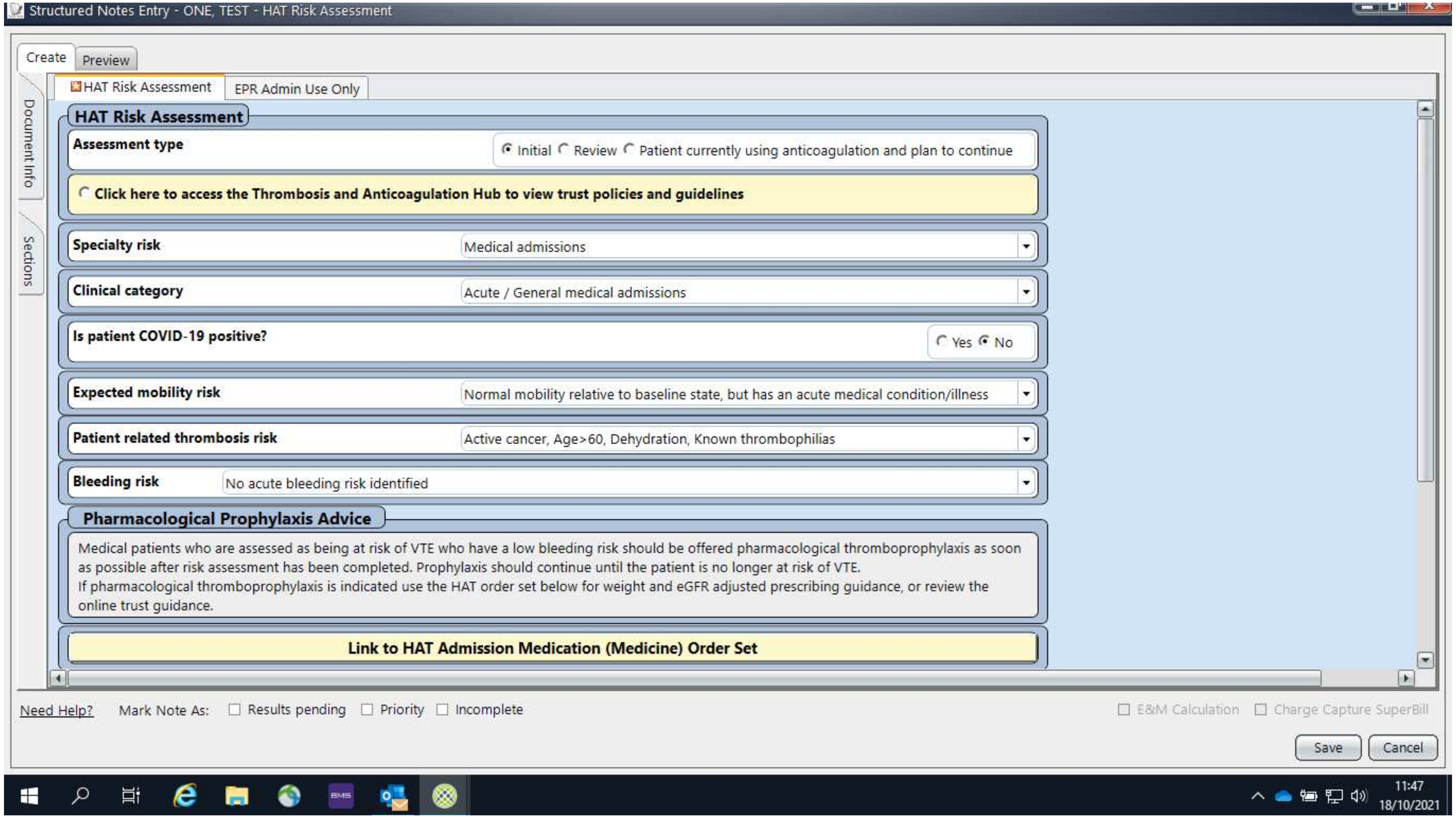

Supplementary material: Evaluating routine data sources to determine the incidence of hospital acquired thrombosis and major bleeding in medical and surgical inpatients.

Site 2 – Paper risk assessment proforma with linked prescribing (Manchester NHS Foundation Trust)

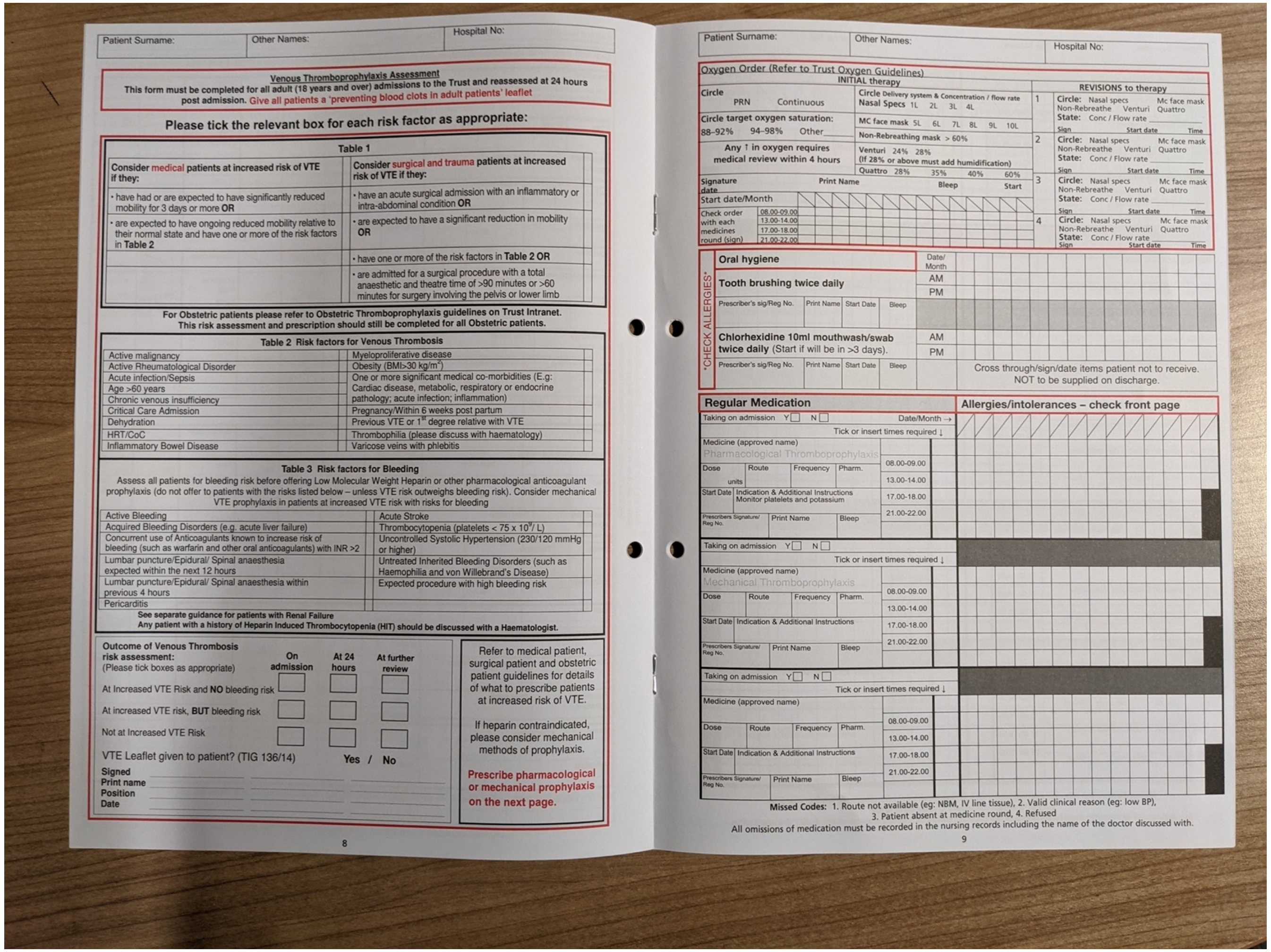

Patient Surname: | Other Names: | Hospital No:

Venous Thromboprophylaxis Assessment

This form must be completed for all adult (18 years and over) admissions to the Trust and reassessed at 24 hours post admission. Give all patients a 'preventing blood clots in adult patients' leaflet

Please tick the relevant box for each risk factor as appropriate:

Table 1

Consider medical patients at increased risk of VTE if they:	Consider surgical and trauma patients at increased risk of VTE if they:
• have had or are expected to have significantly reduced mobility for 3 days or more **OR**	• have an acute surgical admission with an inflammatory or intra-abdominal condition **OR**
• are expected to have ongoing reduced mobility relative to their normal state and have one or more of the risk factors in **Table 2**	• are expected to have a significant reduction in mobility **OR**
	• have one or more of the risk factors in **Table 2 OR**
	• are admitted for a surgical procedure with a total anaesthetic and theatre time of >90 minutes or >60 minutes for surgery involving the pelvis or lower limb

For Obstetric patients please refer to Obstetric Thromboprophylaxis guidelines on Trust Intranet. This risk assessment and prescription should still be completed for all Obstetric patients.

Table 2 Risk factors for Venous Thrombosis

Active malignancy	Myeloproliferative disease
Active Rheumatological Disorder	Obesity (BMI>30 kg/m^2)
Acute infection/Sepsis	One or more significant medical co-morbidities (E.g: Cardiac disease, metabolic, respiratory or endocrine pathology; acute infection; inflammation)
Age >60 years	
Chronic venous insufficiency	
Critical Care Admission	Pregnancy/Within 6 weeks post partum
Dehydration	Previous VTE or 1st degree relative with VTE
HRT/CoC	Thrombophilia (please discuss with haematology)
Inflammatory Bowel Disease	Varicose veins with phlebitis

Table 3 Risk factors for Bleeding

Assess all patients for bleeding risk before offering Low Molecular Weight Heparin or other pharmacological anticoagulant prophylaxis (do not offer to patients with the risks listed below – unless VTE risk outweighs bleeding risk). Consider mechanical VTE prophylaxis in patients at increased VTE risk with risks for bleeding

Active Bleeding	Acute Stroke
Acquired Bleeding Disorders (e.g. acute liver failure)	Thrombocytopenia (platelets < 75 x 10^9/ L)
Concurrent use of Anticoagulants known to increase risk of bleeding (such as warfarin and other oral anticoagulants) with INR >2	Uncontrolled Systolic Hypertension (230/120 mmHg or higher)
Lumbar puncture/Epidural/ Spinal anaesthesia expected within the next 12 hours	Untreated Inherited Bleeding Disorders (such as Haemophilia and von Willebrand's Disease)
Lumbar puncture/Epidural/ Spinal anaesthesia within previous 4 hours	Expected procedure with high bleeding risk
Pericarditis	

See separate guidance for patients with Renal Failure
Any patient with a history of Heparin Induced Thrombocytopenia (HIT) should be discussed with a Haematologist.

**Outcome of Venous Thrombosis risk assessment:** (Please tick boxes as appropriate)	**On admission**	**At 24 hours**	**At further review**
At Increased VTE Risk and **NO** bleeding risk	☐	☐	☐
At increased VTE risk, **BUT** bleeding risk	☐	☐	☐
Not at Increased VTE Risk	☐	☐	☐

VTE Leaflet given to patient? (TIG 136/14) **Yes / No**

Signed
Print name
Position
Date

Refer to medical patient, surgical patient and obstetric patient guidelines for details of what to prescribe patients at increased risk of VTE.

If heparin contraindicated, please consider mechanical methods of prophylaxis.

Prescribe pharmacological or mechanical prophylaxis on the next page.

8

Patient Surname: | Other Names: | Hospital No:

Oxygen Order (Refer to Trust Oxygen Guidelines)

INITIAL therapy

Circle PRN Continuous

Circle target oxygen saturation: 88–92% 94–98% Other

Any ↑ in oxygen requires medical review within 4 hours

Circle Delivery system & Concentration / flow rate
Nasal Specs 1L 2L 3L 4L
MC face mask 5L 6L 7L 8L 9L 10L
Non-Rebreathing mask > 60%
Venturi 24% 28% (If 28% or above must add humidification)
Quattro 28% 35% 40% 60%

Signature date | Print Name | Bleep | Start

Start date/Month

Check order with each medicines round (sign): 08.00-09.00, 13.00-14.00, 17.00-18.00, 21.00-22.00

REVISIONS to therapy

1 Circle: Nasal specs Mc face mask Non-Rebreathe Venturi Quattro State: Conc / Flow rate Sign Start date Time
2 Circle: Nasal specs Mc face mask Non-Rebreathe Venturi Quattro State: Conc / Flow rate Sign Start date Time
3 Circle: Nasal specs Mc face mask Non-Rebreathe Venturi Quattro State: Conc / Flow rate Sign Start date Time
4 Circle: Nasal specs Mc face mask Non-Rebreathe Venturi Quattro State: Conc / Flow rate Sign Start date Time

CHECK ALLERGIES

Oral hygiene Date/ Month

Tooth brushing twice daily AM PM

Prescriber's sig/Reg No. | Print Name | Start Date | Bleep

Chlorhexidine 10ml mouthwash/swab twice daily (Start if will be in >3 days). AM PM

Prescriber's sig/Reg No. | Print Name | Start Date | Bleep

Cross through/sign/date items patient not to receive. NOT to be supplied on discharge.

Regular Medication

Allergies/intolerances – check front page

Taking on admission Y☐ N☐ Date/Month →
Tick or insert times required ↓
Medicine (approved name) Pharmacological Thromboprophylaxis
Dose units | Route | Frequency | Pharm.
Start Date | Indication & Additional Instructions Monitor platelets and potassium
Prescribers Signature/ Reg No. | Print Name | Bleep
08.00-09.00 13.00-14.00 17.00-18.00 21.00-22.00

Taking on admission Y☐ N☐
Tick or insert times required ↓
Medicine (approved name) Mechanical Thromboprophylaxis
Dose | Route | Frequency | Pharm.
Start Date | Indication & Additional Instructions
Prescribers Signature/ Reg No. | Print Name | Bleep
08.00-09.00 13.00-14.00 17.00-18.00 21.00-22.00

Taking on admission Y☐ N☐
Tick or insert times required ↓
Medicine (approved name)
Dose | Route | Frequency | Pharm.
Start Date | Indication & Additional Instructions
Prescribers Signature/ Reg No. | Print Name | Bleep
08.00-09.00 13.00-14.00 17.00-18.00 21.00-22.00

Missed Codes: 1. Route not available (eg: NBM, IV line tissue), 2. Valid clinical reason (eg: low BP), 3. Patient absent at medicine round, 4. Refused

All omissions of medication must be recorded in the nursing records including the name of the doctor discussed with.

9

BMJ Publishing Group Limited (BMJ) disclaims all liability and responsibility arising from any reliance placed on this supplemental material which has been supplied by the author(s)

Supplementary material: Evaluating routine data sources to determine the incidence of hospital acquired thrombosis and major bleeding in medical and surgical inpatients.

Site 3 – Paper risk assessment proforma without linked prescribing (Sheffield Teaching Hospitals NHS Foundation Trust)

Sheffield Teaching Hospitals NHS
NHS Foundation Trust

RISK ASSESSMENT FOR VENOUS THROMBOEMBOLISM (VTE)
FOR MEDICAL PATIENTS

Date of admission: ……………………………………

Date of assessment: ……………………………………

Patients should be risk assessed on admission to hospital, and reassessed whenever their clinical situation changes.

Tick all boxes that apply:

Name:

Date of birth:

Hospital No:

Consultant:

□ Medical patients with a reduction in mobility or acute illness: →	Continue full risk assessment of thrombosis and bleeding risk factors below
□ Medical patients with **normal mobility and without acute illness** (including cancer patients solely admitted for therapy) →	Thromboprophylaxis not usually required. *Risk assessment complete – sign box below*
□ Medical patients already on therapeutic anticoagulation (e.g. heparin, warfarin, dabigatran, rivaroxaban, apixaban e.t.c) *with no intention to change* →	Continue existing anticoagulation *Risk assessment complete – sign box below*

Thrombosis (VTE) Risk Factors

Patient related risk factors
- □ Active cancer or cancer treatment
- □ Age over 60 yrs
- □ Dehydration
- □ One or more significant comorbidities (e.g. heart disease; metabolic, endocrine or respiratory pathologies)
- □ Acute infection or inflammatory condition
- □ Personal history or first-degree relative with a history of VTE
- □ Obesity (BMI greater than $30kg/m^2$)
- □ Use of oestrogen-containing contraceptive therapy
- □ Use of hormone replacement therapy (HRT)
- □ Varicose veins with phlebitis
- □ Known thrombophilia
- □ Pregnant or less than 6 weeks post partum – complete *antenatal (PD7262)* or *postnatal (PD7263) risk assessment for venous thromboembolism in pregnancy* form as appropriate

Admission related risk factors
- □ Significantly reduced mobility for 3 days or more (relative to normal state)
- □ Hip or knee replacement surgery
- □ Hip fracture
- □ Total anaesthetic plus surgical time greater than 90mins
- □ Surgery involving pelvis or lower limb with a total anaesthetic plus surgical time greater than 60mins
- □ Acute surgical admission with inflammatory or intra-abdominal condition
- □ Critical care admission
- □ Surgery with significant reduction in mobility

Bleeding Risk Factors

Patient related risk factors
- □ Active bleeding
- □ Acquired bleeding
- □ Uncontrolled hypertension (greater than or equal to 230mmHg systolic or 120mmHg diastolic)
- □ Acute stroke
- □ Thrombocytopaenia (platelets less than $75 \times 10^9/L$)
- □ Untreated inherited bleeding disorders (e.g. haemophilia and von Willebrand's disease)

Admission related risk factors
- □ Neurosurgery, spinal surgery or eye surgery
- □ Other procedure with high bleeding risk
- □ Lumbar puncture/ epidural/ spinal anaesthesia expected within the next 12 hours
- □ Lumbar puncture/ epidural/ spinal anaesthesia within the previous 4 hours

Action taken (for full advice see "STH Guidelines for the prevention of Venous Thromboembolic Disease")
- □ No thrombosis risk factors present so no prophylaxis needed
- □ Thrombosis risk factors present but no prophylaxis prescribed: *state reason why (e.g. already on anticoagulation therapy, bleeding risk outweighs thrombosis risk)*……………………………

Thrombosis risk factors present:
- □ dalteparin prescribed
- □ mechanical prophylaxis prescribed (if dalteparin contraindicated)

If thrombosis risk factors are present, prescribe dalteparin UNLESS bleeding risk outweighs VTE risk, or dalteparin is contraindicated; see below for doses:

**Patient's weight** to nearest kilogram	**eGFR greater than or equal to 20 mL/min/1.73m²**	**eGFR less than 20 mL/min/1.73m²**	**Pregnant women**
**Less than 45kg**	2500 units once daily	2500 units once daily	Refer to *"Risk assessment for VTE in pregnancy and up to 6 weeks postpartum* or *"STH Guidelines for the prevention of Venous Thromboembolic Disease"*
**45-99kg**	5000 units once daily		
**100-149kg**	7500 units once daily		
**150kg and greater**	5000 units twice a day		

8 of 12

Supplementary material: Evaluating routine data sources to determine the incidence of hospital acquired thrombosis and major bleeding in medical and surgical inpatients.

Site 4 – Electronic form within prescribing electronic health record designed to trigger consideration of VTE risk assessment at the point of hospital admission and prompt prescribing in accordance with local guidelines (St Thomas' NHS Foundation Trust).

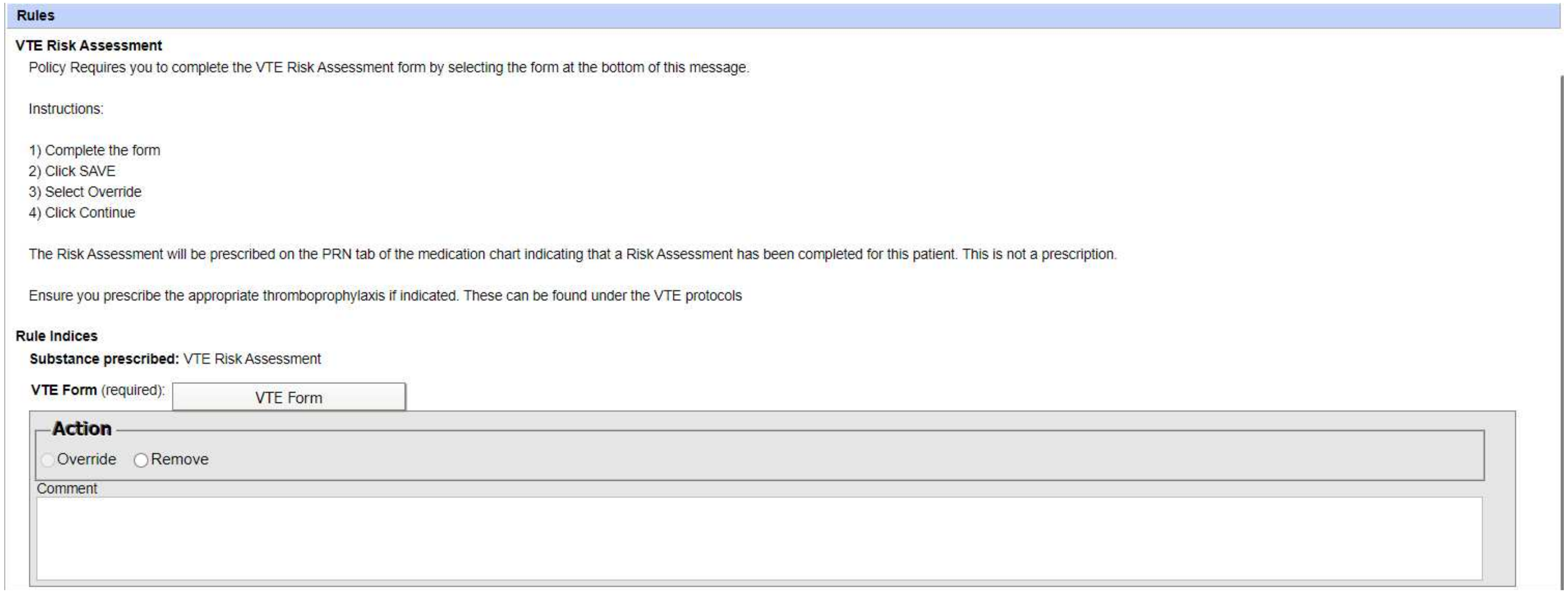
Rules

VTE Risk Assessment

Policy Requires you to complete the VTE Risk Assessment form by selecting the form at the bottom of this message.

Instructions:

1) Complete the form
2) Click SAVE
3) Select Override
4) Click Continue

The Risk Assessment will be prescribed on the PRN tab of the medication chart indicating that a Risk Assessment has been completed for this patient. This is not a prescription.

Ensure you prescribe the appropriate thromboprophylaxis if indicated. These can be found under the VTE protocols

Rule Indices

Substance prescribed: VTE Risk Assessment

VTE Form (required): VTE Form

Action

Override Remove

Comment

Supplementary data

Figure 2: Workflow diagram. CTRU – Clinical Trials Research Unit. VTEAM – Venous Thromboembolism Assessment Models (VTEAM) Study

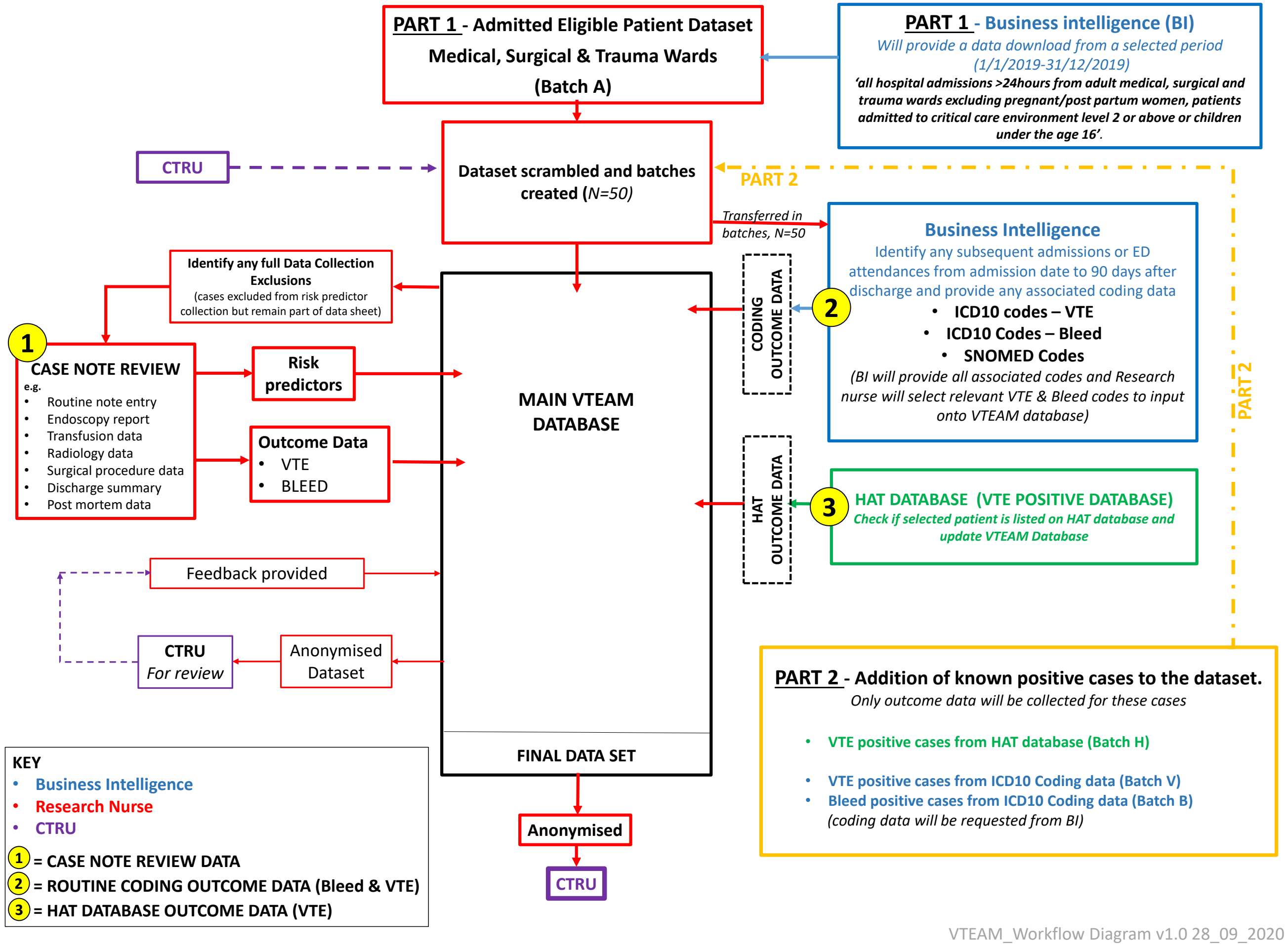

Supplementary data

Table 1: Relevant ICD10 codes for VTE and Bleeding agreed by chief investigators and approved by project management group

ICD10 - 4 digit	3 character description	4 character description	All diagnoses	Main diagnosis	Category	Sub-Category	Final selection
I26.0	Pulmonary embolism	Pulmonary embolism with mention of acute cor pulmonale	4031	2353	VTE		Yes
I26.9	Pulmonary embolism	Pulmonary embolism without mention of acute cor pulmonale	108637	53273	VTE		Yes
I80.1	Phlebitis and thrombophlebitis	Phlebitis and thrombophlebitis of femoral vein	10156	4294	VTE		Yes
I80.2	Phlebitis and thrombophlebitis	Phlebitis and thrombophlebitis of other deep vessels of lower extremities	61647	24297	VTE		Yes
I80.3	Phlebitis and thrombophlebitis	Phlebitis and thrombophlebitis of lower extremities, unspecified	3971	1876	VTE		Yes
I80.9	Phlebitis and thrombophlebitis	Phlebitis and thrombophlebitis of unspecified site	2906	524	VTE		Yes
I82.2	Other venous embolism and thrombosis	Embolism and thrombosis of vena cava	3891	543	VTE		Yes
I82.8	Other venous embolism and thrombosis	Embolism and thrombosis of other specified veins	10001	1870	VTE		Yes
I82.9	Other venous embolism and thrombosis	Embolism and thrombosis of unspecified vein	1124	215	VTE		Yes
I60.0	Subarachnoid haemorrhage	Subarachnoid haemorrhage from carotid siphon and bifurcation	226	212	Bleeding	intracranial bleed	Yes
I60.1	Subarachnoid haemorrhage	Subarachnoid haemorrhage from middle cerebral artery	1125	1014	Bleeding	intracranial bleed	Yes
I60.2	Subarachnoid haemorrhage	Subarachnoid haemorrhage from anterior communicating artery	1731	1599	Bleeding	intracranial bleed	Yes
I60.3	Subarachnoid haemorrhage	Subarachnoid haemorrhage from posterior communicating artery	899	838	Bleeding	intracranial bleed	Yes
I60.4	Subarachnoid haemorrhage	Subarachnoid haemorrhage from basilar artery	384	324	Bleeding	intracranial bleed	Yes
I60.5	Subarachnoid haemorrhage	Subarachnoid haemorrhage from vertebral artery	101	87	Bleeding	intracranial bleed	Yes
I60.6	Subarachnoid haemorrhage	Subarachnoid haemorrhage from other intracranial arteries	564	513	Bleeding	intracranial bleed	Yes

Supplementary data

I60.7	Subarachnoid haemorrhage	Subarachnoid haemorrhage from intracranial artery, unspecified	426	319	Bleeding	intracranial bleed	Yes
I60.8	Subarachnoid haemorrhage	Other subarachnoid haemorrhage	977	737	Bleeding	intracranial bleed	Yes
I60.9	Subarachnoid haemorrhage	Subarachnoid haemorrhage, unspecified	7642	4585	Bleeding	intracranial bleed	Yes
I61.0	Intracerebral haemorrhage	Intracerebral haemorrhage in hemisphere, subcortical	4996	4396	Bleeding	intracranial bleed	Yes
I61.1	Intracerebral haemorrhage	Intracerebral haemorrhage in hemisphere, cortical	5439	4254	Bleeding	intracranial bleed	Yes
I61.2	Intracerebral haemorrhage	Intracerebral haemorrhage in hemisphere, unspecified	1431	1157	Bleeding	intracranial bleed	Yes
I61.3	Intracerebral haemorrhage	Intracerebral haemorrhage in brain stem	1029	866	Bleeding	intracranial bleed	Yes
I61.4	Intracerebral haemorrhage	Intracerebral haemorrhage in cerebellum	1901	1508	Bleeding	intracranial bleed	Yes
I61.5	Intracerebral haemorrhage	Intracerebral haemorrhage, intraventricular	3678	1886	Bleeding	intracranial bleed	Yes
I61.6	Intracerebral haemorrhage	Intracerebral haemorrhage, multiple localized	764	561	Bleeding	intracranial bleed	Yes
I61.8	Intracerebral haemorrhage	Other intracerebral haemorrhage	3854	3103	Bleeding	intracranial bleed	Yes
I61.9	Intracerebral haemorrhage	Intracerebral haemorrhage, unspecified	11863	9028	Bleeding	intracranial bleed	Yes
I62.0	Other nontraumatic intracranial haemorrhage	Subdural haemorrhage (acute)(nontraumatic)	17161	8197	Bleeding	intracranial bleed	Yes
I62.1	Other nontraumatic intracranial haemorrhage	Nontraumatic extradural haemorrhage	318	100	Bleeding	intracranial bleed	Yes
I62.9	Other nontraumatic intracranial haemorrhage	Intracranial haemorrhage (nontraumatic), unspecified	3230	2383	Bleeding	intracranial bleed	Yes
I85.0	Oesophageal varices	Oesophageal varices with bleeding	4074	2876	Bleeding	gastrointestinal	Yes
K22.6	Other diseases of oesophagus	Gastro-oesophageal laceration-haemorrhage syndrome	7232	3237	Bleeding		Yes
K25.0	Gastric ulcer	Gastric ulcer - Acute with haemorrhage	2077	1469	Bleeding	gastrointestinal	Yes
K25.2	Gastric ulcer	Gastric ulcer - Acute with both haemorrhage and perforation	49	20	Bleeding	gastrointestinal	Yes

Supplementary data

K25.4	Gastric ulcer	Gastric ulcer - Chronic or unspecified with haemorrhage	4742	2951	Bleeding	gastrointestinal	Yes
K25.6	Gastric ulcer	Gastric ulcer - Chronic or unspecified with both haemorrhage and perforation	145	74	Bleeding	gastrointestinal	Yes
K26.0	Duodenal ulcer	Duodenal ulcer - Acute with haemorrhage	2955	2161	Bleeding	gastrointestinal	Yes
K26.2	Duodenal ulcer	Duodenal ulcer - Acute with both haemorrhage and perforation	126	96	Bleeding	gastrointestinal	Yes
K26.4	Duodenal ulcer	Duodenal ulcer - Chronic or unspecified with haemorrhage	7607	4972	Bleeding	gastrointestinal	Yes
K26.6	Duodenal ulcer	Duodenal ulcer - Chronic or unspecified with both haemorrhage and perforation	386	263	Bleeding	gastrointestinal	Yes
K27.0	Peptic ulcer, site unspecified	Peptic ulcer, site unspecified - Acute with haemorrhage	78	32	Bleeding	gastrointestinal	Yes
K27.2	Peptic ulcer, site unspecified	Peptic ulcer, site unspecified - Acute with both haemorrhage and perforation	4	1	Bleeding	gastrointestinal	Yes
K27.4	Peptic ulcer, site unspecified	Peptic ulcer, site unspecified - Chronic or unspecified with haemorrhage	231	116	Bleeding	gastrointestinal	Yes
K27.6	Peptic ulcer, site unspecified	Peptic ulcer, site unspecified - Chronic or unspecified with both haemorrhage and perforation	29	9	Bleeding	gastrointestinal	Yes
K28.0	Gastrojejunal ulcer	Gastrojejunal ulcer - Acute with haemorrhage	29	24	Bleeding	gastrointestinal	Yes
K28.2	Gastrojejunal ulcer	Gastrojejunal ulcer - Acute with both haemorrhage and perforation	3	3	Bleeding	gastrointestinal	Yes
K28.4	Gastrojejunal ulcer	Gastrojejunal ulcer - Chronic or unspecified with haemorrhage	149	96	Bleeding	gastrointestinal	Yes
K28.6	Gastrojejunal ulcer	Gastrojejunal ulcer - Chronic or unspecified with both haemorrhage and perforation	11	6	Bleeding	gastrointestinal	Yes
K29.0	Gastritis and duodenitis	Acute haemorrhagic gastritis	3340	1365	Bleeding	gastrointestinal	Yes
K62.5	Other diseases of anus and rectum	Haemorrhage of anus and rectum	37545	21106	Bleeding	gastrointestinal	Yes
K66.1	Other disorders of peritoneum	Haemoperitoneum	3317	642	Bleeding	gastrointestinal	Yes
K92.0	Other diseases of digestive system	Haematemesis	67589	27503	Bleeding	gastrointestinal	Yes
K92.1	Other diseases of digestive system	Melaena	67036	22979	Bleeding	gastrointestinal	Yes
K92.2	Other diseases of digestive system	Gastrointestinal haemorrhage, unspecified	192053	97428	Bleeding	gastrointestinal	Yes

Supplementary data

M25.0	Other joint disorders, not elsewhere classified	Haemarthrosis	3362	1730	Bleeding		Yes
N02.0	Recurrent and persistent haematuria	Recurrent and persistent haematuria - Minor glomerular abnormality	48	21	Bleeding		Yes
N02.1	Recurrent and persistent haematuria	Recurrent and persistent haematuria - Focal and segmental glomerular lesions	286	76	Bleeding		Yes
N02.2	Recurrent and persistent haematuria	Recurrent and persistent haematuria - Diffuse membranous glomerulonephritis	1858	517	Bleeding		Yes
N02.3	Recurrent and persistent haematuria	Recurrent and persistent haematuria - Diffuse mesangial proliferative glomerulonephritis	160	49	Bleeding		Yes
N02.4	Recurrent and persistent haematuria	Recurrent and persistent haematuria - Diffuse endocapillary proliferative glomerulonephritis	10	4	Bleeding		Yes
N02.5	Recurrent and persistent haematuria	Recurrent and persistent haematuria - Diffuse mesangiocapillary glomerulonephritis	47	12	Bleeding		Yes
N02.6	Recurrent and persistent haematuria	Recurrent and persistent haematuria - Dense deposit disease	9	1	Bleeding		Yes
N02.7	Recurrent and persistent haematuria	Recurrent and persistent haematuria - Diffuse crescentic glomerulonephritis	164	48	Bleeding		Yes
N02.8	Recurrent and persistent haematuria	Recurrent and persistent haematuria - Other	10852	1289	Bleeding		Yes
N02.9	Recurrent and persistent haematuria	Recurrent and persistent haematuria - Unspecified	2902	1331	Bleeding		Yes
N93.8	Other abnormal uterine and vaginal bleeding	Other specified abnormal uterine and vaginal bleeding	4801	2899	Bleeding	other bleed	Yes
N93.9	Other abnormal uterine and vaginal bleeding	Abnormal uterine and vaginal bleeding, unspecified	24423	11036	Bleeding	other bleed	Yes
R04.0	Haemorrhage from respiratory passages	Epistaxis	48741	24610	Bleeding	other bleed	Yes
R04.1	Haemorrhage from respiratory passages	Haemorrhage from throat	191	69	Bleeding	other bleed	Yes
R04.2	Haemorrhage from respiratory passages	Haemoptysis	32143	12743	Bleeding	other bleed	Yes
R04.8	Haemorrhage from respiratory passages	Haemorrhage from other sites in respiratory passages	1332	222	Bleeding	other bleed	Yes
R04.9	Haemorrhage from respiratory passages	Haemorrhage from respiratory passages, unspecified	83	23	Bleeding	other bleed	Yes

Supplementary data

R23.3	Other skin changes	Spontaneous ecchymoses	10624	2774	Bleeding		Yes
R58.X	Haemorrhage, not elsewhere classified	Haemorrhage, not elsewhere classified	2747	408	Bleeding		Yes
T81.0	Complications of procedures, not elsewhere classified	Haemorrhage and haematoma complicating a procedure, not elsewhere classified	67338	28601	Bleeding		Yes
T81.7	Complications of procedures, not elsewhere classified	Vascular complications following a procedure, not elsewhere classified	1000	201	Bleeding		Yes
