# Supplementary material for: Accuracy of efficient data methods to determine the incidence of hospital-acquired thrombosis and major bleeding in medical and surgical inpatients: a multicentre observational cohort study in four UK hospitals
Source: BMJ Open. 2023 Feb 6;13(2):e069244. doi: 10.1136/bmjopen-2022-069244 (PMC9906300; doi:10.1136/bmjopen-2022-069244)
Supplement: Author's manuscript [file bmjopen-2022-069244.draft_revisions.pdf]

BMJ Open

BMJ Open is committed to open peer review. As part of this commitment we make the peer review history of every article we publish publicly available.

When an article is published we post the peer reviewers' comments and the authors' responses online. We also post the versions of the paper that were used during peer review. These are the versions that the peer review comments apply to.

The versions of the paper that follow are the versions that were submitted during the peer review process. They are not the versions of record or the final published versions. They should not be cited or distributed as the published version of this manuscript.

BMJ Open is an open access journal and the full, final, typeset and author-corrected version of record of the manuscript is available on our site with no access controls, subscription charges or pay-per-view fees (http://bmjopen.bmj.com).

If you have any questions on BMJ Open's open peer review process please email info.bmjopen@bmj.com

BMJ Open

Evaluating routine data sources to determine the incidence of hospital acquired thrombosis and major bleeding in medical and surgical inpatients.

Journal:	*BMJ Open*
Manuscript ID	bmjopen-2022-069244
Article Type:	Original research
Date Submitted by the Author:	14-Oct-2022
Complete List of Authors:	Horner, Daniel; Northern Care Alliance NHS Foundation Trust, Emergency Department; The University of Manchester, Division of Infection, Immunity and Respiratory Medicine<br>Rex, Saleema; University of Sheffield, ScHARR<br>Reynard, Charles; The University of Manchester, Division of Cardiovascular sciences;<br>Bursnall, Matthew; The University of Sheffield, School of Health and Related Research (ScHARR)<br>Bradburn, Mike; University of Sheffield, ScHARR<br>de Wit, Kerstin; McMaster University Department of Medicine, Department of Medicine; Hamilton General Hospital<br>Goodacre, Steve; University of Sheffield, Medical Care Research Unit<br>Hunt, Beverley; Guy's and St Thomas' NHS Foundation Trust, Kings Healthcare Partners & Thrombosis & Haemophilia Centre
Keywords:	Anticoagulation < HAEMATOLOGY, Thromboembolism < CARDIOLOGY, Risk management < HEALTH SERVICES ADMINISTRATION & MANAGEMENT

SCHOLARONE™
Manuscripts

I, the Submitting Author has the right to grant and does grant on behalf of all authors of the Work (as defined in the below author licence), an exclusive licence and/or a non-exclusive licence for contributions from authors who are: i) UK Crown employees; ii) where BMJ has agreed a CC-BY licence shall apply, and/or iii) in accordance with the terms applicable for US Federal Government officers or employees acting as part of their official duties; on a worldwide, perpetual, irrevocable, royalty-free basis to BMJ Publishing Group Ltd ("BMJ") its licensees and where the relevant Journal is co-owned by BMJ to the co-owners of the Journal, to publish the Work in this journal and any other BMJ products and to exploit all rights, as set out in our licence.

The Submitting Author accepts and understands that any supply made under these terms is made by BMJ to the Submitting Author unless you are acting as an employee on behalf of your employer or a postgraduate student of an affiliated institution which is paying any applicable article publishing charge ("APC") for Open Access articles. Where the Submitting Author wishes to make the Work available on an Open Access basis (and intends to pay the relevant APC), the terms of reuse of such Open Access shall be governed by a Creative Commons licence – details of these licences and which Creative Commons licence will apply to this Work are set out in our licence referred to above.

Other than as permitted in any relevant BMJ Author's Self Archiving Policies, I confirm this Work has not been accepted for publication elsewhere, is not being considered for publication elsewhere and does not duplicate material already published. I confirm all authors consent to publication of this Work and authorise the granting of this licence.

For peer review only

Title:

Evaluating routine data sources to determine the incidence of hospital acquired thrombosis and major bleeding in medical and surgical inpatients.

Author names and positions:

Daniel Horner MD [1, 2]	Consultant in Emergency and Intensive Care Medicine
Saleema Rex[3]	Research Associate
Charlie Reynard [4]	Postgraduate Research Fellow
Matt Bursnall [3]	Research Associate
Mike Bradburn [3]	Senior Medical Statistician
Kerstin de Wit [5]	Associate Professor
Steve Goodacre [3]	Professor of Emergency Medicine
Beverley Hunt [6]	Professor of Thrombosis and Haemostasis

Affiliations:

[1] Emergency Department, Northern Care Alliance NHS Foundation Trust, Salford, UK
[2] Division of Infection, Immunity and Respiratory Medicine, University of Manchester, Oxford Road, UK
[3] Centre for Urgent and Emergency Care Research (CURE), University of Sheffield, Sheffield, UK
[4] Division of Cardiovascular Sciences, University of Manchester, Oxford Road, Manchester, UK
[5] Department of Medicine, McMaster University, Hamilton, Ontario, Canada.
[6] Kings Healthcare Partners & Thrombosis & Haemophilia Centre, Guy's & St Thomas' NHS Foundation Trust, London, UK

Corresponding author:

Daniel Horner daniel.horner@nca.nhs.uk
Mailing address: Emergency Department, Northern Care Alliance NHS Foundation Trust, Stott Lane, Salford, M6 8HD

Word Counts:

Abstract word count: 246
Total word count: 3870
References: 23

Competing Interests Statement:

On behalf of all authors, I declare the following competing interests:
During completion of this study, SG, DH, CR, BH, MBu and MB received funding from the National Institute of Health Research for academic work in this area, through competitive grant application and appoint to a doctoral research fellow position (CR).

Study Registration:

This project is registered at https://fundingawards.nihr.ac.uk/award/NIHR127454

For peer review only

Funding Information:

This study was funded by the United Kingdom National Institute for Health Research (NIHR) Health Technology Assessment (HTA) Programme (project number 127454). The views expressed in this report are those of the authors and not necessarily those of the NIHR HTA Programme. Any errors are the responsibility of the authors. The funders had no role in the study design, in the collection, analysis and interpretation of data; in the writing of the manuscript; and in the decision to submit the manuscript for publication.

Notation of prior abstract publication/presentation:

Sections of this work were presented at the ISTH 2022 conference in London by poster. This work is also accepted for publication by the NIHR in monograph format within their journals library in April 2023. The NIHR strongly encourages wide dissemination of funded work and early submission of manuscripts, as per the embargo policy:

"In line with our embargo policy, we are prepared to delay publication of your Journals Library manuscript and, where possible, co-ordinate publication of your Journals Library manuscript with any journal articles."

More details on the NIHR Embargo policy can be found at the following address: https://www.journalslibrary.nihr.ac.uk/policies/#Embargo

Keywords:

Venous Thromboembolism

Risk Assessment

Thromboprophylaxis

Routine data

Efficient methods

For peer review only

Abstract

Objectives:

We evaluated the accuracy of using routine health service data to identify hospital acquired thrombosis (HAT) and major bleeding events (MBE) compared to a reference standard of case note review.

Design:

A multi-centre observational cohort study.

Setting:

Four acute hospitals in the United Kingdom.

Participants:

A consecutive unselective cohort of general medical and surgical patients requiring hospitalisation for a period of >24h during the calendar year 2021. We excluded paediatric, obstetric and critical care patients due to differential risk profiles.

Interventions:

We compared routinely collected information using hospital coding data and local contractually mandated thrombosis datasets, to data extracted from case notes using a predesigned workflow methodology.

Primary and Secondary Outcome Measures:

We defined HAT as objectively confirmed venous thromboembolism occurring during hospital stay or within 90 days of discharge and MBE as per international consensus.

Results:

We were able to collect the necessary routine data from 87% of 2008 case episodes examined. The sensitivity of hospital coding data (ICD-10) for the diagnosis of HAT and MBE was 62% (95% CI 54 to 69) and 38% (95% CI 27 to 50) respectively. Sensitivity improved to 81% (95% CI 75 to 87) when using local thrombosis databsets.

Conclusions:

Using routine data instead of case note review will miss a substantial proportion of outcome events. Our study suggests that currently available routine data collection methods in the UK are inadequate to support efficient study designs in venous thromboembolism research.

Trial registration:

This project is registered at https://fundingawards.nihr.ac.uk/award/NIHR127454

For peer review only

Article Summary:

Strengths and Limitations of this study

- This observational cohort study recruited over 2000 patients across four NHS trusts to evaluate the accuracy of routine data to identify hospital acquired thrombosis and major bleeding events, compared to case note review.

- We worked with patients, clinical experts and researchers to develop a detailed workflow pathway and iterative dataset, conforming to international outcome definitions.

- Data abstractors were blinded to routine data sources throughout extraction, limiting bias in case ascertainment.

- We only evaluated general medical and surgical hospital admissions; we did not assess the accuracy of routine data for outcomes in complex cohorts such as cancer or neurosurgical patients.

- We only evaluated routinely collected and available data among four large urban hospitals and in a UK healthcare setting. Our findings may therefore lack generalisability to other settings.

For peer review only

BACKGROUND

Venous Thromboembolism (VTE) remains a major global health burden, with significant attributable morbidity and mortality.[1] At least half of all VTE occurs during hospitalisation, or up to 90 days following discharge; such cases are described as Hospital Acquired Thrombosis (HAT).[2] Many of these events are potentially preventable through patient education and provision of thromboprophylaxis to those at risk.

Research into thromboprophylaxis often requires large sample sizes to identify small but important differences in clinically relevant events, such as HAT and/or major bleeding. Study protocols will often necessitate examination of case notes to identify outcome events, which can be time consuming and expensive. This is particularly relevant for external validation of new clinical decision rules or risk assessment models which aim to guide prescribing of thromboprophylaxis for hospital inpatients.[3-5]

Using routine health service data to identify outcome events could markedly improve the efficiency of research and facilitate studies with large sample sizes at acceptable cost. However, this approach requires confirmatory evidence that routine data sources accurately identify outcome events.

Several mechanisms already exist for routine identification of outcomes, including hospital coding, local VTE datasets, and pathology reporting (with thrombosis committee oversight). If such efficient methods could accurately ascertain relevant outcomes, large scale studies would be theoretically deliverable.

We sought to evaluate the accuracy of using routine data to identify HAT and major bleeding events (MBE) compared to case note examination.

For peer review only

METHODS

We conducted a multi-centre observational cohort study within the context of a wider project examining the overall clinical and cost effectiveness of VTE risk assessment models.[6] The aim of this study was to estimate the accuracy and completeness of available coding data and local registry data to determine clinically relevant VTE and bleeding outcomes against case note review by trained research assistants.

We approached four sites to participate in this study; the Northern Care Alliance NHS Foundation Trust, Manchester University Hospitals NHS Foundation Trust, the Northern General Hospital in Sheffield and Guy's & St Thomas' NHS Foundation Trust.

Study Population

We identified a consecutive, unselected cohort of general medical and surgical patients requiring hospital admission at each site during the calendar year 1st January to 31st December 2019. We chose 2019 because of concern that patients admitted during the subsequent COVID-19 pandemic might represent an atypical cohort. We collated data on all risk assessments that clinical staff performed prospectively at the point of hospital admission, then scrambled episodes into randomly assorted batches of 50 (A batches) to ensure diversity in specialty presentation and mitigate seasonal bias. We excluded paediatric patients (age <16), anyone requiring critical care admission (defined as level 2 care or above) and pregnant/post-partum patients due to differential VTE and bleeding risks, as outlined in the wider study protocol.[7]

Study design

For each patient episode, we extracted baseline demographics and prospectively collected data on VTE risk assessment (where available) from the electronic health record, with support from business intelligence teams. Risk assessments were captured differently at each site, including the use of a paper proforma, dichotomous output on electronic prescribing (low/high risk), or through a detailed structured note within the electronic healthcare record. Example images/screenshots for each site can be found in the supplementary appendix. All four sites used the Department of Health tool to facilitate VTE risk assessment.

For peer review only

Research assistants at each site undertook retrospective case note review for each patient episode through shared primary and secondary care electronic health records. We extracted descriptive data on relevant clinical outcomes such as the subsequent diagnosis of VTE/HAT, major bleeding and clinically relevant non-major bleeding events as per internationally agreed definitions.[8 9] Data extractors were trained in identification of these outcomes and followed a detailed workflow diagram (supplementary material). Data abstractors were blinded to batch allocation, final ICD-10 coding, local database entries and the final analysis plan.

Following case note review, we collated multiple routine data sources for each patient episode to evaluate their accuracy against case note review outcomes. Data sources for interrogation were identified a-priori and included the following; ICD-10 diagnostic codes judged relevant to thrombosis or bleeding by the project management group (a-priori, shown in the supplementary data); Emergency Care Data Set/SNOMED codes relevant to thrombosis or bleeding; and contractual local Hospital Acquired Thrombosis (HAT) databases. HAT database entries were co-ordinated by local thrombosis committees in accordance with NHS contract standards. These datasets include a contemporary register of all patients diagnosed with acute VTE at the hospital site (informed by radiology/pathology), with subsequently categorisation as HAT based on preceding hospital admission (up to 90 days) or VTE diagnosis >24h following hospital admission. All data sources were interrogated for the duration of hospital stay and up to 90 days post discharge, for each patient episode. Data sources were obtained through routine local business intelligence requests or direct approach to local coding teams. HAT database entries were obtained where feasible through local site thrombosis committee chairs.

Given the potential for negligible VTE/MBE in the wider study population (leading to limited information on the accuracy of efficient data methods), we augmented the overall sample with positive thrombosis and bleeding cases. We obtained positive cases through ICD-10 coding identification for VTE events (V batches), bleeding events (B batches) and local HAT database entries (H batches). Positive cases were batched and reviewed in accordance with the general study protocol. Data extractors were blinded to batch allocation.

For peer review only

Outcomes

The following criteria were proposed to determine whether routine data identify outcome events with sufficient accuracy to support efficient methods:

1. Proportion of outcome events identified by routine data sources that are confirmed by record review (target 100%)

2. Proportion of cases with no outcome event identified by routine data sources that have an event identified on record review (target 0%)

3. Proportion of inpatients with data collected (target 90%)

Statistical analysis

The accuracy of routinely recorded HAT and bleeding events was compared against direct case note review data for the cohort. Case note review determination of events was assumed to be the gold standard. Data are presented in contingency tables with sensitivity, specificity, positive and negative predictive values along with confidence intervals calculated using the Wilson score method.[10]

The primary analysis included patients identified from all sources (A, V, B or H batches) with bleeding and HAT assumed to have not occurred unless coded as such in the relevant data, or detected following case note review. In addition, two pre-planned sensitivity analyses were undertaken:

a. Inclusion was limited to cases identified in routine case review (i.e. “A batch patients” only), with exclusion of all augmented sample cases.
b. Inclusion limited to participants for whom bleeding or HAT was definitively recorded

We took a conservative approach and interpreted missing or unknown endpoints as ‘no event’ with the exception of the second sensitivity analysis.

We originally planned to identify 3000 inpatients across 4 hospitals during a 12-month study period within the 2-year project plan, dependent on appointment of research assistants and time required for outcome ascertainment. This sample size was designed to allow key parameters to be estimated with a high degree of precision across the whole cohort (standard error <1%). All sites failed to meet their sample target of 750 for reasons mainly related to the SARs COV-2 pandemic, including redeployment of

For peer review only

research staff to clinical care, delayed local approvals secondary to prioritised pandemic research and a longer than anticipated time for individual outcome ascertainment per case review

Ethical Aspects

The study received a favourable opinion from the Proportionate Review Sub-committee of the London - West London & GTAC Research Ethics Committee and approval from the HRA and Care Research Wales (HCRW) on 18th September 2019 (reference 19/LO/1303, IRAS project ID 262220).

Participating sites identified members of the clinical care team (research nurses or assistants predominately) to access patient records and extract clinical data using a predesigned and protected Microsoft Excel© database with embedded macro function, hosted at site. All data subsequently underwent local de-identification following completion and were exported to an independent team of statisticians at the Clinical Trials Research Unit (CTRU) in Sheffield, for collation and analysis.

All aspects of the data collection process, export, analysis and oversight were regularly reviewed by the internal Project Management Group (PMG) including CTRU representation, and an external Trial Steering Committee (TSC), throughout the duration of the project.

We conducted this study in accordance with international EQUATOR guidelines. A STROBE reporting checklist was used throughout to inform design, conduct and analysis of this observational cohort study and is included as supplementary information.

Patient and Public Involvement

Representatives of two Patient and Public Involvement groups (PPI), thrombosis UK and Sheffield Emergency Care Forum joined the research team and were involved in developing the initial proposal and undertaking the wider study.

For peer review only

The Sheffield Emergency Care Forum (SECF) is a patient and public representative group with an interest in emergency care research. The forum has provided PPI for many emergency care research projects over then last ten years.[11] Thrombosis UK is a charity that aims to identify, inform and partner the NHS, healthcare providers and individuals to work to improve prevention of VTE and the management and care of VTE events (see https://www.thrombosisuk.org/).

The PPI members were involved in determining the study design and ensuring that the proposal addressed the needs of patients and the NHS, while respecting the needs of potential participants. Their input regarding the importance of providing thromboprophylaxis for potential participants of any prospective cohort study and the need for such a study to yield reliable findings was instrumental in determining our approach to answering the research question. The PPI members also provided input at project management meetings and, where required, in day-to-day running of the project. The members used meetings and surveys of their wider PPI groups to enhance PPI in the project.

For peer review only

RESULTS

We identified 2115 patients with an original hospital admission occurring in the calendar year 2019. Of these, 107 patient episodes were ineligible due to being pregnant or post-partum women (n=49); admitted to a critical care environment of level 2 or above (n=38); children aged under 16 (n=13) or for unrecorded reasons (n=7) leaving 2008 episodes for analysis. All episodes were suitable for data extraction and comparison to routine data sources.

Patient episodes showed an even balance of medical and surgical cases, but with a focus on emergency (73.7%) rather than elective (25.8%) admissions. A broad range of subspecialty interests were represented within the cohort. Median length of stay was 3 days (IQR 3 to 8) and mean length of stay 7.75 days (SD 16.5). Specialty groups with frequencies and cumulative percentages are shown in Table 1. The vast majority of patient episodes (1809, 90.1%) were taken from 'A' batches. The total sample was augmented by 45 patients (2.2%) with potential bleeding events and 154 (7.7%) patients with potential VTE events. All sites contributed evenly to the sample with one exception; reduced numbers at this site reflect a delay to institutional approval during the pandemic, arising from a high burden of other clinical research studies and high staff turnover. Site and batch numbers are shown in Table 2

Main Findings

Contingency tables for the accuracy of routine data sources compared to case note review for both HAT and major bleeding events are shown in Table 3. Sensitivity was 62% (95% CI 54 to 69) for the use of ICD-10/SNOMED coding data to detect HAT events and 81% (95% CI 75 to 87) for local HAT database entries. Sensitivity by individual site ranged from 45% (95% CI, 28 to 63) to 72% (95% CI, 61 to 82) using ICD-10/SNOMED coding and 68% (95% CI, 51 to 84) to 94% (95% CI 87 to 100) using local HAT database entries.

The sensitivity of ICD10/SNOMED coding to detect major bleeding events identified by case note review was 38% (95% CI 27 to 50). Sensitivity by individual site ranged from 22% (95% CI 0 to 49) to 56% (95% CI 37 to 75).

For peer review only

Pre-planned Sensitivity Analysis

A sensitivity analysis was conducted using only patient episodes obtained through 'A' batches, to remove augmentation of the sample and mitigate bias. The sensitivity of efficient data methods to detect key outcomes identified at case note review remained poor. These results are summarised in Table 4.

We found the HAT event rate on case note review to be 29/1809 (1.6%, 95% CI 1.0 to 2.2) and the major bleeding event rate to be 45/1809 (2.5%, 95% CI 1.8 to 3.2) within this large cohort of hospitalised patients receiving risk assessment and thromboprophylaxis in the context of routine care.

The proportion of outcome HAT events identified by routine data sources that were confirmed by record review (target 100%) was 71% (95% CI 63 to 79) for ICD-10/SNOMED coding and 100% (95% CI 97 to 100) for local HAT database entries. The proportion of cases with no HAT outcome event identified by routine data sources that had an event identified on record review (target 0%) was 3% (95% CI 2 to 4) for ICD-10/SNOMED coding and 2% (95% CI 1 to 2) for local HAT database entries. The proportion of major bleeding events identified by routine data sources that were confirmed by record review (target 100%) was 20% (95% CI 13 to 27) for ICD-10/SNOMED coding. The proportion of cases with no major bleeding outcome event identified by routine data sources that have an event identified on record review (target 0%) was 2% (95% CI 1 to 3) for ICD-10/SNOMED coding. We were able to collect outcome data for 1745/2008 (87%) inpatients (target 90%). This was <100% due to difficulty accessing the local HAT database at a single site. Excluding this issue, the other three sites all managed to collect relevant outcome data for at least 98% of patients.

For peer review only

DISCUSSION

Statement of principal findings

Our findings suggest that using currently available routine data for identification of HAT and MBE during hospital admission or within 90 days of discharge is not sufficiently sensitive to support a large data-enabled study. We failed to demonstrate feasibility for a number of predefined metrics and conclude that use of routine data to identify outcomes would be highly likely to miss important events, and may erroneously identify false positive events.

Strengths and weaknesses of the study

We engaged a combination of digitally mature and paper-based UK NHS sites in this study, used strict consensus definitions for VTE/bleeding events and evaluated only predefined efficient data sources. We also used topic experts and research staff to iteratively develop our data collection tool and workflow diagram, to limit subjective interpretation of case note data. However, there are limitations to this work. We evaluated patient episodes from large urban hospital sites, two of which were VTE exemplar centres and three of which were tertiary centres, which may limit external validity. Research assistants across sites varied in seniority and clinical experience; although all sites had a principal investigator and strict working definitions for outcome events, this may have introduced variation in reporting. Finally, we did not achieve our intended target of 3000 patients. However, it is important to note that the overall results within our cohort of 2008 patients are well outside of feasibility targets and sensitivity values were universally poor. We do not envisage that adding further cases would have significantly affected these values.

Strengths and weaknesses in relation to other studies, discussing important differences in results

Previous international work in this area is conflicting. A comparison of hospital episode statistics (HES) data to general practice records in England reported in 2012, initially concluded reliable identification of vascular disease (derived from ICD-10 coding data).[12] However, this analysis was restricted to pulmonary embolism from a VTE perspective and sought only to correlate disease states, rather than identify new case episodes. Several authors have used primary care research datasets correlated to

For peer review only

evidence of anticoagulation or other secondary care data to identify VTE events, with reported reliable capture. This work does not seek to discriminate between index presentation of VTE and downstream development of hospital acquired thrombosis.[13 14]

A systematic review, with searches run in July 2010 and published in 2012, summarised findings on this topic from nineteen studies. The positive predictive value (PPV) for pulmonary embolism ICD-10 codes ranged from 24% to 92%, with higher values from certain combinations of codes. PPV values for DVT codes ranged from 31% to 97%. More recently, a cross sectional North American study compared ICD-10 codes for VTE in hospitalised medical patients to a 'gold standard' manual review of clinical data in 4000 patients.[15] The authors report a sensitivity of 63% for any DVT and a sensitivity of 83% for PE, implying further discrepancy between types of VTE. Our findings align with these latter reports but offer additional validation of HAT states (in addition to VTE diagnosis) compared with routine data.

Several authors have experimented with composite data sets and diagnostic/procedural/disease coding combinations, similar to our work. One study combined ICD-10 codes for VTE with a common procedural terminology code for a VTE Diagnostic Study plus at least one of the following within 30 days of diagnosis; pharmacy script for anticoagulation, placement of an inferior vena cava filter, or death.[16] This algorithm still lacked sensitivity, reporting a value of 0.67 (0.60, 0.73) although corresponding specificity was high at 0.99 (0.98, 0.99). Alotaibi *et al* subsequently combined routinely collected ICD-10 coding data with imaging procedure codes to identify VTE events over a ten-year period, compared to case note review. Again, they report highly specific results but limited sensitivity, in line with our findings (74.83% (95% CI 67.01-81.62) and 75.24% (95% CI 65.86 to 83.14) for PE and DVT, respectively).[17] Verma et al report using natural language processing (NLP) algorithms for digital interrogation of radiology reports in a large cohort of hospitalised medical patients to identify VTE outcomes.[15] The authors conclude a high level of accuracy, reporting sensitivities of 94% / 91% and PPVs of 90% / 89% for DVT and PE, respectively. Finally, Klil-Drori et al have recently validated an algorithm for confirmation of suspected PE, combining emergency department diagnosis coding, imaging coding and dispensed prescription or hospital treatment.[18] The authors report overall agreement of their algorithm with confirmed PE (adjudicated through chart review) in 92.2% cases. Again, such an algorithim would not discriminate between

For peer review only

index diagnosis of VTE and subsequent development of HAT. Such algorithms also require external validation in a UK setting.

In 2017, Baumgartner et al highlighted further issues through interrogation of an administrative coding database, looking to determine the accuracy of ICD-10 coding for new episodes of recurrent VTE in patients with a prior history.[19] Only 31.1% of coded encounters were verified by reviewers as true recurrent VTE. More recently, Pellathy *et al* have conducted similar work within the United States, comparing accuracy of HAT diagnoses made through administrative coding to manual case note and radiology review.[20] The authors report only 40% of HAT cases identified through routine coding were confirmed by case note review and 45% of HAT confirmed through diagnostic test records lacked corresponding ICD codes.

Meaning of the study

There are multiple potential explanations for the limited performance of routine data to identify HAT. The condition is a temporal phenomenon and routine coding data can therefore mistake index presentation with VTE as HAT, or fail to code subsequent HAT following index admission with alternative pathology (such as pneumonia). International guidelines support outpatient diagnosis and management of VTE, so genuine cases of HAT may not require hospital admission or receive appropriate coding. Prior diagnosis of VTE can often be coded during repeat hospital attendance and mistaken as HAT.

Many UK hospitals conducting root cause analysis of HAT cases in line with NHS contract standards have developed pathways to mitigate these issues, through local reporting arrangements with radiology, ultrasound and pathology services. Such arrangements often work well transiently, but are reliant on individuals and reporting systems subject to human error. These issues are reflected in our findings, which report a positive predictive value of 100% for HAT RCA database findings, but limited sensitivity (implying local identification of positive cases is accurate, but missed cases still occur despite a systematic approach).

Possible explanations and implications for clinicians and policymakers

More generally, these findings raise questions about the current enthusiasm for data enabled trials when outcomes are complex.[21] Such concepts are inherently attractive to researchers and patients, particularly in topic areas with low event rates. However,

For peer review only

complex outcome measures which require temporal evaluation and qualification against prior disease states are unlikely to be reliably delivered through use of routinely collected data in isolation. For example, relevant data may contain coding errors arising from ambiguous documentation by physicians and inconsistent definitions.[22 23] Recent case studies have reported significant amounts of missing data and poor interobserver agreement between routinely collected EHR data accessible through HES and case report form evaluation.[24] Electronic records contain an abundance of free text, but often lack necessary intelligence to classify patient episodes appropriately, or allow processing and comparison of routinely collected data.[25] Increasing complexity in outcome is also likely to correspond with decreasing accuracy of routine data. A registry study of Medicare claims following mitral valve repair compared to formal adjudication, reported a positive predictive value for mortality of 97%, heart failure requiring hospitalisation of 69%, bleeding of 40% and renal failure of 19%.[26]

In addition, the time and effort needed to acquire necessary permissions for national routine coding data or to orchestrate data linkage can be substantial. A UK clinical trials unit recently reported a digital request in the context of a randomised controlled trial, highlighting a negotiation process over consent that took several years. Even after consent, the study team were in receipt of data 15 months following application.[27] Such timeframes may only be realistic within the context of continually adaptive design trials.

Unanswered questions and future research

This work is restricted primarily to medical, surgical and orthopaedic patients. We did not evaluate efficient data methods for VTE or bleeding events in specific patient subgroups, such as cancer or neurosurgery. In addition, our work is UK based; other countries may be able to demonstrate more confidence in the accuracy of routinely collected data, although our review of the literature does not support this theory.

In their call to action, Sydes et al discuss supplementation of trial specific follow up as an option to realise the full potential of data-enabled research.[21] Such an approach has potential merit to attempt identification of potential HAT, given the high positive predictive value and high specificity of routine data sources. In addition, routine data sources may have a role in other research contexts, such as identification of cases for qualitative work, case control studies, targeted individual follow up or downstream survey work.

For peer review only

CONCLUSIONS

Our study highlights the potential limitations of using routine data methods in the context of future research on VTE risk assessment. Such methods identify both false negative and false positive VTE cases, through failure to identify ambulatory cases without formal hospital coding and overdiagnosis of prior disease. Our findings were similar with regard to bleeding events, showing poor sensitivity of ICD-10 coding data and multiple false positive events identified across four sites. These findings have implications for funders looking to support further work in this area and suggest large studies reliant on routine data collection methods in isolation are likely to be inaccurate and therefore unfeasible.

For peer review only

Author statement

The authors were involved as follows: SG and DH (conception), RD, CR, SG, BH and DH (execution, analysis and drafting manuscript). SR designed and developed the iterative database. MBu and MBr conducted statistical evaluation of the dataset on behalf of the CTRU. KdW and BH attended PMG meetings and contributed to drafting of the final manuscript. All authors were involved in critical discussion, revision and final approval of the manuscript. DH acts as guarantor. The corresponding author attests that all listed authors meet authorship criteria and that no others meeting the criteria have been omitted.

Acknowledgements

We would like to acknowledge the support of the research nurses and assistants involved in chart review, data extraction and entry across the four hospital sites, including Reece Doonan, Efia Mainoo, Linda Debattista, Sarah Bird and Anna Wilson. We would also like to acknowledge the wider group directly conducting the VTEAM project (NIHR 127454), including project manager Helen Shulver, literature expert Abdullah Pandor, clerical assistant Heather Dakin, topic expert Xavier Griffin and clinical expert Mark Holland. We would also like to acknowledge the valuable input from the patient and public representatives, Robin Pierce-Williams, Chris Tweedy, Ben Langsdale, Deb Smith (Thrombosis UK), Shan Bennett and Enid Hirst (Sheffield Emergency Care Forum).

Data Sharing Statement

Data are not publicly available but may be obtained or interrogated via written request to the clinical trials research unit at the University of Sheffield.

For peer review only

REFERENCES

1. Raskob GE, Angchaisuksiri P, Blanco AN, et al. Thrombosis: a major contributor to global disease burden. *Semin Thromb Hemost* 2014;40(7):724-35. doi: 10.1055/s-0034-1390325 [published Online First: 2014/10/11]
2. Heit JA, O'Fallon WM, Petterson TM, et al. Relative impact of risk factors for deep vein thrombosis and pulmonary embolism: a population-based study. *Arch Intern Med* 2002;162(11):1245-8. doi: 10.1001/archinte.162.11.1245 [published Online First: 2002/06/01]
3. Horner D, Goodacre S, Davis S, et al. Which is the best model to assess risk for venous thromboembolism in hospitalised patients? *BMJ* 2021;373:n1106. doi: 10.1136/bmj.n1106 [published Online First: 2021/05/29]
4. Riley RD, Ensor J, Snell KI, et al. External validation of clinical prediction models using big datasets from e-health records or IPD meta-analysis: opportunities and challenges. *BMJ* 2016;353:i3140. doi: 10.1136/bmj.i3140 [published Online First: 2016/06/24]
5. Steyerberg EW, Vergouwe Y. Towards better clinical prediction models: seven steps for development and an ABCD for validation. *Eur Heart J* 2014;35(29):1925-31. doi: 10.1093/eurheartj/ehu207 [published Online First: 2014/06/06]
6. Goodacre S, Hogg K, Holland M, et al. The cost-effectiveness of venous thromboembolism risk assessment tools for hospital inpatients https://fundingawards.nihr.ac.uk/: NIHR; 2019 [Available from: https://fundingawards.nihr.ac.uk/award/NIHR127454 accessed 19/10/2021 2021.
7. Goodacre SG, Horner D, Hogg K, et al. The cost-effectiveness of venous thromboembolism risk assessment tools for hospital inpatients fundingawards.nihr.ac.uk: NIHR; 2020 [Available from: https://fundingawards.nihr.ac.uk/award/NIHR127454 accessed 1/7 2020.
8. Schulman S, Angeras U, Bergqvist D, et al. Definition of major bleeding in clinical investigations of antihemostatic medicinal products in surgical patients. *J Thromb Haemost* 2010;8(1):202-4. doi: 10.1111/j.1538-7836.2009.03678.x [published Online First: 2009/11/03]
9. Schulman S, Kearon C, Subcommittee on Control of Anticoagulation of the S, et al. Definition of major bleeding in clinical investigations of antihemostatic medicinal products in non-surgical patients. *J Thromb Haemost* 2005;3(4):692-4. doi: 10.1111/j.1538-7836.2005.01204.x [published Online First: 2005/04/22]
10. Newcombe RG. Two-sided confidence intervals for the single proportion: comparison of seven methods. *Stat Med* 1998;17(8):857-72. doi: 10.1002/(sici)1097-0258(19980430)17:8<857::aid-sim777>3.0.co;2-e [published Online First: 1998/05/22]
11. Hirst E, Irving A, Goodacre S. Patient and public involvement in emergency care research. *Emerg Med J* 2016;33(9):665-70. doi: 10.1136/emermed-2016-205700 [published Online First: 2016/04/06]
12. Wright FL, Green J, Canoy D, et al. Vascular disease in women: comparison of diagnoses in hospital episode statistics and general practice records in England. *BMC Med Res Methodol* 2012;12:161. doi: 10.1186/1471-2288-12-161 [published Online First: 2012/11/01]

For peer review only

13. Abdul Sultan A, Tata LJ, Grainge MJ, et al. The incidence of first venous thromboembolism in and around pregnancy using linked primary and secondary care data: a population based cohort study from England and comparative meta-analysis. *PLoS One* 2013;8(7):e70310. doi: 10.1371/journal.pone.0070310 [published Online First: 2013/08/08]
14. Lawrenson R, Todd JC, Leydon GM, et al. Validation of the diagnosis of venous thromboembolism in general practice database studies. *Br J Clin Pharmacol* 2000;49(6):591-6. doi: 10.1046/j.1365-2125.2000.00199.x [published Online First: 2000/06/10]
15. Verma AA, Masoom H, Pou-Prom C, et al. Developing and validating natural language processing algorithms for radiology reports compared to ICD-10 codes for identifying venous thromboembolism in hospitalized medical patients. *Thromb Res* 2021;209:51-58. doi: 10.1016/j.thromres.2021.11.020 [published Online First: 2021/12/07]
16. Sanfilippo KM, Wang TF, Gage BF, et al. Improving accuracy of International Classification of Diseases codes for venous thromboembolism in administrative data. *Thromb Res* 2015;135(4):616-20. doi: 10.1016/j.thromres.2015.01.012 [published Online First: 2015/01/24]
17. Alotaibi GS, Wu C, Senthilselvan A, et al. The validity of ICD codes coupled with imaging procedure codes for identifying acute venous thromboembolism using administrative data. *Vasc Med* 2015;20(4):364-8. doi: 10.1177/1358863X15573839 [published Online First: 2015/04/03]
18. Klil-Drori AJ, Prajapati D, Liang Z, et al. External Validation of ASPECT (Algorithm for Suspected Pulmonary Embolism Confirmation and Treatment). *Med Care* 2019;57(8):e47-e52. doi: 10.1097/MLR.0000000000001055 [published Online First: 2019/01/05]
19. Baumgartner C, Go AS, Fan D, et al. Administrative codes inaccurately identify recurrent venous thromboembolism: The CVRN VTE study. *Thromb Res* 2020;189:112-18. doi: 10.1016/j.thromres.2020.02.023 [published Online First: 2020/03/22]
20. Pellathy T, Saul M, Clermont G, et al. Accuracy of identifying hospital acquired venous thromboembolism by administrative coding: implications for big data and machine learning research. *J Clin Monit Comput* 2021 doi: 10.1007/s10877-021-00664-6 [published Online First: 2021/02/10]
21. Sydes MR, Barbachano Y, Bowman L, et al. Realising the full potential of data-enabled trials in the UK: a call for action. *BMJ Open* 2021;11(6):e043906. doi: 10.1136/bmjopen-2020-043906 [published Online First: 2021/06/18]
22. Spencer FA, Emery C, Lessard D, et al. The Worcester Venous Thromboembolism study: a population-based study of the clinical epidemiology of venous thromboembolism. *J Gen Intern Med* 2006;21(7):722-7. doi: 10.1111/j.1525-1497.2006.00458.x [published Online First: 2006/07/01]
23. Burles K, Innes G, Senior K, et al. Limitations of pulmonary embolism ICD-10 codes in emergency department administrative data: let the buyer beware. *BMC Med Res Methodol* 2017;17(1):89. doi: 10.1186/s12874-017-0361-1 [published Online First: 2017/06/10]
24. Powell GA, Bonnett LJ, Smith CT, et al. Using routinely recorded data in a UK RCT: a comparison to standard prospective data collection methods. *Trials* 2021;22(1):429. doi: 10.1186/s13063-021-05294-6 [published Online First: 2021/07/07]

For peer review only

25. Liaw ST, Chen HY, Maneze D, et al. Health reform: is routinely collected electronic information fit for purpose? *Emerg Med Australas* 2012;24(1):57-63. doi: 10.1111/j.1742-6723.2011.01486.x [published Online First: 2012/02/09]
26. Lowenstern A, Lippmann SJ, Brennan JM, et al. Use of Medicare Claims to Identify Adverse Clinical Outcomes After Mitral Valve Repair. *Circ Cardiovasc Interv* 2019;12(5):e007451. doi: 10.1161/CIRCINTERVENTIONS.118.007451 [published Online First: 2019/05/16]
27. Macnair A, Love SB, Murray ML, et al. Accessing routinely collected health data to improve clinical trials: recent experience of access. *Trials* 2021;22(1):340. doi: 10.1186/s13063-021-05295-5 [published Online First: 2021/05/12]

For peer review only

Table 1: Clinical category and admission type, with frequency and cumulative percentage

	Frequency	Percentage	Cumulative
**Admission type**			
Missing	1	0.05	0.05
Elective	518	25.80	25.85
Emergency	1,480	73.71	99.55
Unknown	9	0.45	100
Total	2,008	100	
**Specialty Group**			
Missing	9	0.45	0.45
Medical	902	44.92	45.37
Surgical	951	47.36	92.73
Tertiary specialty	146	7.27	100
Total	2,008	100	
**Clinical Category**			
Missing	9	0.45	0.45
Acute medicine	340	16.93	17.38
Ageing and complex medicine	133	6.62	24.0
Cardiology	41	2.04	26.04
Cardiothoracic Surgery	87	4.33	30.38
Dermatology	2	0.1	30.48
Emergency Medicine	87	4.33	34.81
Gastroenterology	61	3.04	37.85
General Surgery	285	14.19	52.04
Medical: Other	169	8.42	60.46
Neurology	10	0.5	60.96
Neurorehabilitation	2	0.1	61.06
Neurosurgery	39	1.94	63.0
Gynaecology	57	2.84	65.84
Renal Medicine	26	1.29	67.13
Respiratory	63	3.14	70.27
Rheumatology	2	0.1	70.37
Trauma and Orthopaedics	158	7.87	78.24
Upper GI Surgery	13	0.65	78.89
Urology	107	5.33	84.22
Surgery: Other	170	8.47	92.69
Tertiary specialty: Other	147	7.32	100
Total	2,008	100	

For peer review only

Table 2: Number of cases submitted by site and batch type

A – Patient admissions requiring routine risk assessment
B – Potential cases of bleeding (selected from relevant ICD-10 codes)
H – Cases of Hospital Acquired Thrombosis (HAT) identified through local thrombosis committee infrastructure
V - Potential cases of venous thromboembolic disease (selected from relevant ICD-10 codes)

	Batch				Total
	A	B	H	V	
GSTT	504	0	21	0	525
Manchester	241	0	0	0	241
Salford	570	45	44	46	705
Sheffield	494	0	43	0	537
Total	1809	45	108	46	2008

For peer review only

Table 3: Contingency tables for main outcomes

HAT = Hospital Acquired Thrombosis
*Manchester site excluded from this analysis as unable to access local HAT database.

		**HAT from case note review**		
		Yes	No	
**HAT from ICD10/SNOMED codes**	Yes	95	39	71% (63%, 79%) True positive rate and 95% CI
	No	59	1815	3% (2%, 4%) False negative rate and 95% CI
		62% (54%, 69%) Sensitivity and 95% CI	98% (97%, 99%) Specificity and 95% CI	(N=2008)
		Yes	No	
**HAT from HAT RCA database**	Yes	122	0	100% (100%, 100%) True positive rate and 95% CI
	No	29	1616	2% (1%, 2%) False negative rate and 95% CI
		81% (75%, 87%) Sensitivity and 95% CI	100% (100%, 100%) Specificity and 95% CI	(N=1767)*
		**Major bleed from case note review**		
		Yes	No	
**Major Bleed from ICD10/SNOMED codes**	Yes	25	98	20% (13%, 27%) True positive rate and 95% CI
	No	40	1845	2% (1%, 3%) False negative rate and 95% CI
		38% (27%, 50%) Sensitivity and 95% CI	95% (94%, 96%) Specificity and 95% CI	(N=2008)

For peer review only

Table 4: Sensitivity analysis using only A batch cases. N=1809 following removal of H/B/V batch patients. *Manchester unable to access HAT database

		**HAT from case note review**		
		Yes	No	
**HAT from ICD10/SNOMED codes**	Yes	18	18	50% (34%, 66%) True positive rate and 95% CI
	No	11	1762	1% (0%, 1%) False negative rate and 95% CI
		62% (44%, 80%) Sensitivity and 95% CI	99% (99%, 99%) Specificity and 95% CI	(N=1809)
		Yes	No	
**HAT from HAT RCA database**	Yes	7	0	100% (100%, 100%) True positive rate and 95% CI
	No	19	1542	1% (1%, 2%) False negative rate and 95% CI
		27% (10%, 44%) Sensitivity and 95% CI	100% (100%, 100%) Specificity and 95% CI	(N=1568)*
		**Major bleed from case note review**		
		Yes	No	
**Bleed from ICD10/SNOMED codes**	Yes	14	68	17% (9%, 25%) True positive rate and 95% CI
	No	31	1696	2% (1%, 2%) False negative rate and 95% CI
		31% (18%, 45%) Sensitivity and 95% CI	96% (95%, 97%) Specificity and 95% CI	(N=1809)

For peer review only

Supplementary material: Evaluating routine data sources to determine the incidence of hospital acquired thrombosis and major bleeding in medical and surgical inpatients.

Figure 1: Examples of electronic and paper documentation aids for RAM completion

Site 1- Structured note completed through Electronic Health Record and designed to aid VTE risk assessment at the point of hospital admission, using the Department of Health RAM (Salford Royal NHS Foundation Trust).

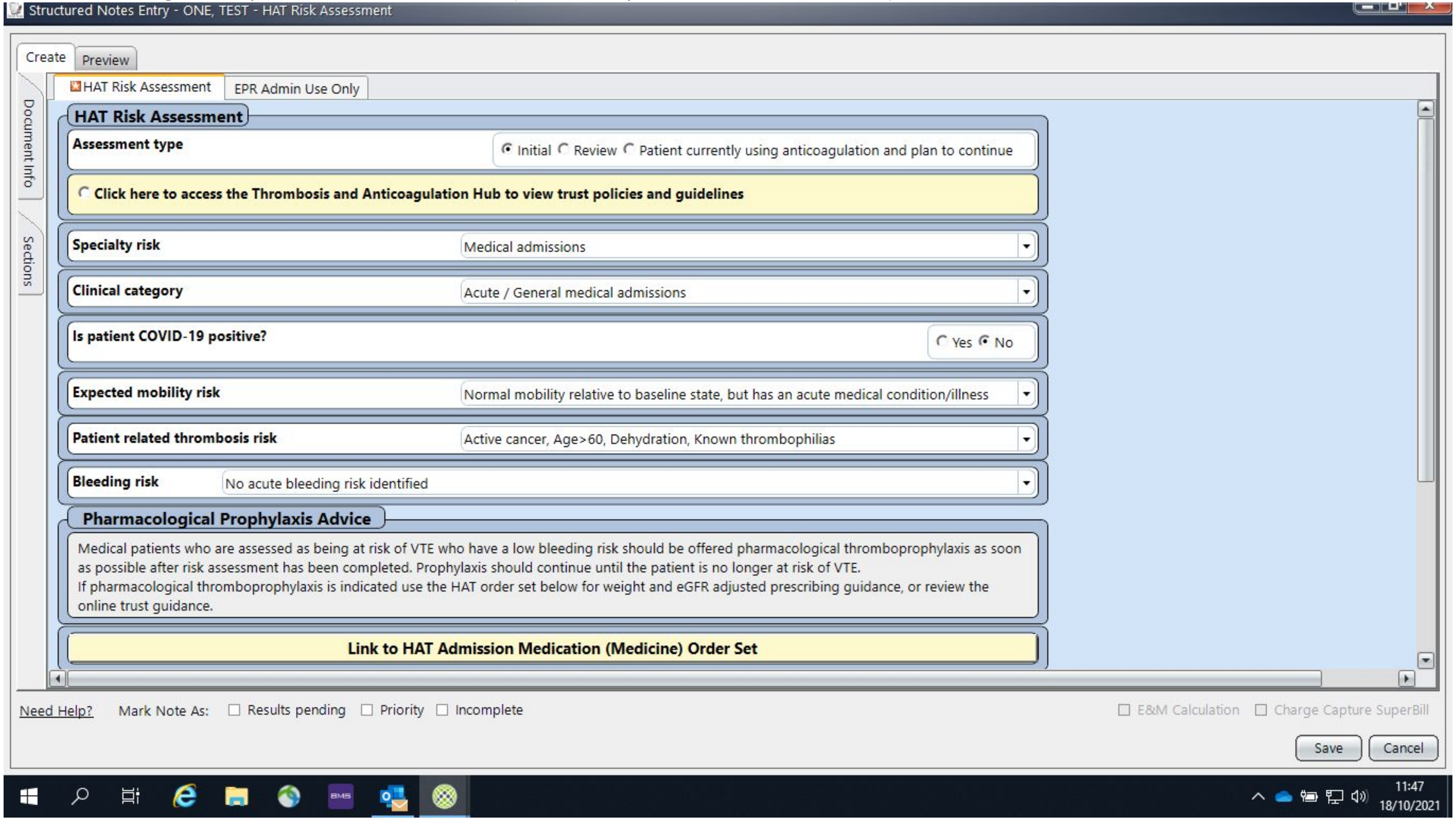

Supplementary material: Evaluating routine data sources to determine the incidence of hospital acquired thrombosis and major bleeding in medical and surgical inpatients.

Site 2 – Paper risk assessment proforma with linked prescribing (Manchester NHS Foundation Trust)

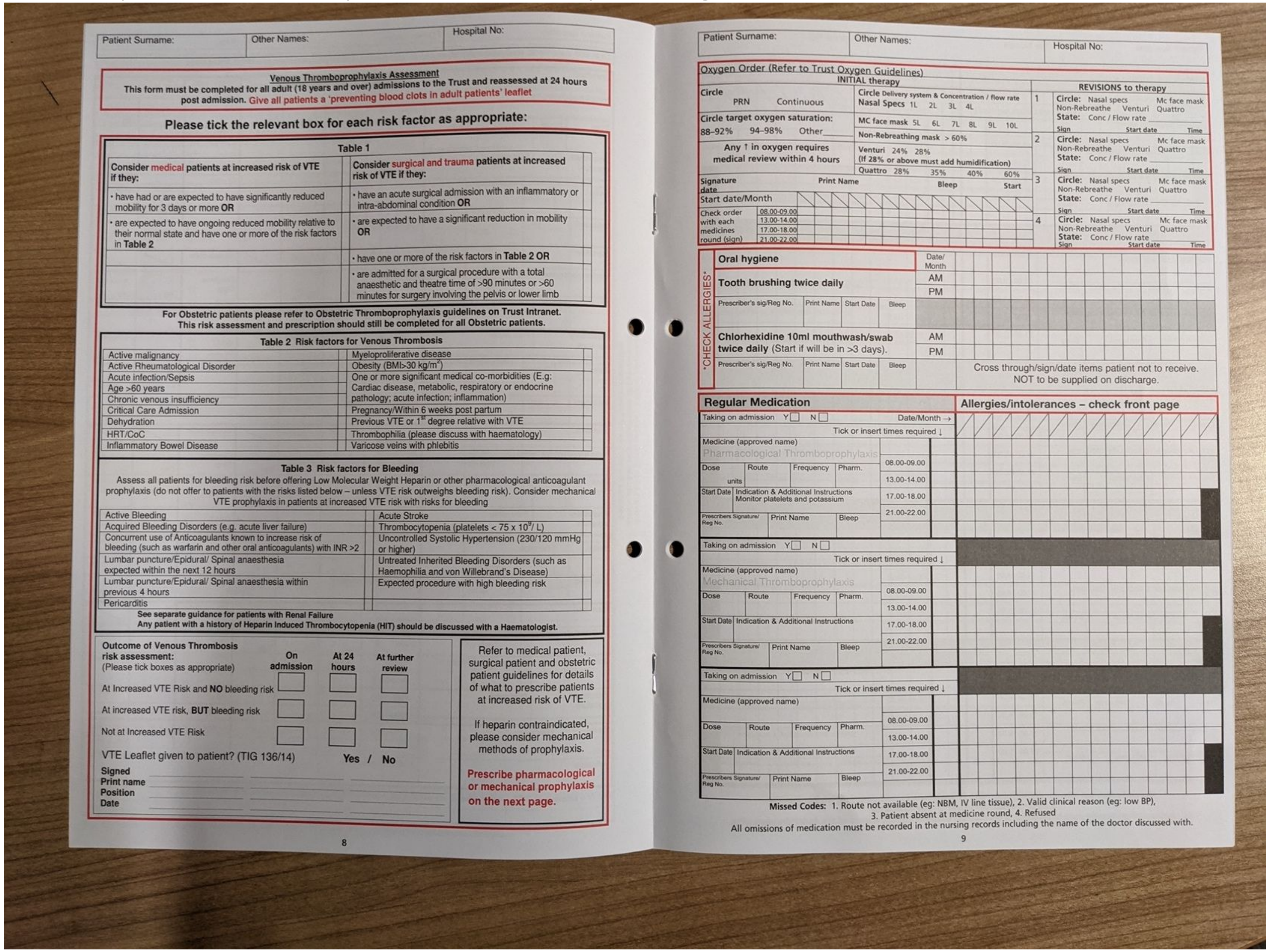

Patient Surname: | Other Names: | Hospital No:

Venous Thromboprophylaxis Assessment

This form must be completed for all adult (18 years and over) admissions to the Trust and reassessed at 24 hours post admission. Give all patients a 'preventing blood clots in adult patients' leaflet

Please tick the relevant box for each risk factor as appropriate:

Table 1

Consider medical patients at increased risk of VTE if they:		Consider surgical and trauma patients at increased risk of VTE if they:	
• have had or are expected to have significantly reduced mobility for 3 days or more **OR**		• have an acute surgical admission with an inflammatory or intra-abdominal condition **OR**	
• are expected to have ongoing reduced mobility relative to their normal state and have one or more of the risk factors in **Table 2**		• are expected to have a significant reduction in mobility **OR**	
		• have one or more of the risk factors in **Table 2 OR**	
		• are admitted for a surgical procedure with a total anaesthetic and theatre time of >90 minutes or >60 minutes for surgery involving the pelvis or lower limb	

For Obstetric patients please refer to Obstetric Thromboprophylaxis guidelines on Trust Intranet. This risk assessment and prescription should still be completed for all Obstetric patients.

Table 2 Risk factors for Venous Thrombosis

Active malignancy		Myeloproliferative disease	
Active Rheumatological Disorder		Obesity (BMI>30 kg/m^2)	
Acute infection/Sepsis		One or more significant medical co-morbidities (E.g: Cardiac disease, metabolic, respiratory or endocrine pathology; acute infection; inflammation)	
Age >60 years			
Chronic venous insufficiency			
Critical Care Admission		Pregnancy/Within 6 weeks post partum	
Dehydration		Previous VTE or 1st degree relative with VTE	
HRT/CoC		Thrombophilia (please discuss with haematology)	
Inflammatory Bowel Disease		Varicose veins with phlebitis	

Table 3 Risk factors for Bleeding

Assess all patients for bleeding risk before offering Low Molecular Weight Heparin or other pharmacological anticoagulant prophylaxis (do not offer to patients with the risks listed below – unless VTE risk outweighs bleeding risk). Consider mechanical VTE prophylaxis in patients at increased VTE risk with risks for bleeding

Active Bleeding		Acute Stroke	
Acquired Bleeding Disorders (e.g. acute liver failure)		Thrombocytopenia (platelets < 75 x 10^9/ L)	
Concurrent use of Anticoagulants known to increase risk of bleeding (such as warfarin and other oral anticoagulants) with INR >2		Uncontrolled Systolic Hypertension (230/120 mmHg or higher)	
Lumbar puncture/Epidural/ Spinal anaesthesia expected within the next 12 hours		Untreated Inherited Bleeding Disorders (such as Haemophilia and von Willebrand's Disease)	
Lumbar puncture/Epidural/ Spinal anaesthesia within previous 4 hours		Expected procedure with high bleeding risk	
Pericarditis			

See separate guidance for patients with Renal Failure
Any patient with a history of Heparin Induced Thrombocytopenia (HIT) should be discussed with a Haematologist.

Outcome of Venous Thrombosis risk assessment: (Please tick boxes as appropriate)	On admission	At 24 hours	At further review
At Increased VTE Risk and **NO** bleeding risk	☐	☐	☐
At increased VTE risk, **BUT** bleeding risk	☐	☐	☐
Not at Increased VTE Risk	☐	☐	☐

VTE Leaflet given to patient? (TIG 136/14) Yes / No

Signed
Print name
Position
Date

Refer to medical patient, surgical patient and obstetric patient guidelines for details of what to prescribe patients at increased risk of VTE.

If heparin contraindicated, please consider mechanical methods of prophylaxis.

Prescribe pharmacological or mechanical prophylaxis on the next page.

8

Patient Surname: | Other Names: | Hospital No:

Oxygen Order (Refer to Trust Oxygen Guidelines)

INITIAL therapy

Circle PRN Continuous

Circle Delivery system & Concentration / flow rate
Nasal Specs 1L 2L 3L 4L
MC face mask 5L 6L 7L 8L 9L 10L
Non-Rebreathing mask > 60%
Venturi 24% 28% (If 28% or above must add humidification)
Quattro 28% 35% 40% 60%

Circle target oxygen saturation: 88–92% 94–98% Other

Any ↑ in oxygen requires medical review within 4 hours

Signature date | Print Name | Bleep | Start

Start date/Month

Check order with each medicines round (sign) 08.00-09.00 13.00-14.00 17.00-18.00 21.00-22.00

REVISIONS to therapy

1 Circle: Nasal specs Mc face mask Non-Rebreathe Venturi Quattro State: Conc / Flow rate Sign Start date Time
2 Circle: Nasal specs Mc face mask Non-Rebreathe Venturi Quattro State: Conc / Flow rate Sign Start date Time
3 Circle: Nasal specs Mc face mask Non-Rebreathe Venturi Quattro State: Conc / Flow rate Sign Start date Time
4 Circle: Nasal specs Mc face mask Non-Rebreathe Venturi Quattro State: Conc / Flow rate Sign Start date Time

CHECK ALLERGIES

Oral hygiene Date/Month

Tooth brushing twice daily AM PM
Prescriber's sig/Reg No. | Print Name | Start Date | Bleep

Chlorhexidine 10ml mouthwash/swab twice daily (Start if will be in >3 days). AM PM
Prescriber's sig/Reg No. | Print Name | Start Date | Bleep

Cross through/sign/date items patient not to receive. NOT to be supplied on discharge.

Regular Medication

Allergies/intolerances – check front page

Taking on admission Y☐ N☐ Date/Month →
Tick or insert times required ↓
Medicine (approved name) Pharmacological Thromboprophylaxis
Dose units | Route | Frequency | Pharm. 08.00-09.00 13.00-14.00
Start Date | Indication & Additional Instructions Monitor platelets and potassium 17.00-18.00 21.00-22.00
Prescribers Signature/ Reg No. | Print Name | Bleep

Taking on admission Y☐ N☐
Tick or insert times required ↓
Medicine (approved name) Mechanical Thromboprophylaxis
Dose | Route | Frequency | Pharm. 08.00-09.00 13.00-14.00
Start Date | Indication & Additional Instructions 17.00-18.00 21.00-22.00
Prescribers Signature/ Reg No. | Print Name | Bleep

Taking on admission Y☐ N☐
Tick or insert times required ↓
Medicine (approved name)
Dose | Route | Frequency | Pharm. 08.00-09.00 13.00-14.00
Start Date | Indication & Additional Instructions 17.00-18.00 21.00-22.00
Prescribers Signature/ Reg No. | Print Name | Bleep

Missed Codes: 1. Route not available (eg: NBM, IV line tissue), 2. Valid clinical reason (eg: low BP), 3. Patient absent at medicine round, 4. Refused
All omissions of medication must be recorded in the nursing records including the name of the doctor discussed with.

9

Supplementary material: Evaluating routine data sources to determine the incidence of hospital acquired thrombosis and major bleeding in medical and surgical inpatients.

Site 3 – Paper risk assessment proforma without linked prescribing (Sheffield Teaching Hospitals NHS Foundation Trust)

Sheffield Teaching Hospitals NHS
NHS Foundation Trust

RISK ASSESSMENT FOR VENOUS THROMBOEMBOLISM (VTE) FOR MEDICAL PATIENTS

Date of admission: ....

Date of assessment: ....

Patients should be risk assessed on admission to hospital, and reassessed whenever their clinical situation changes.

Tick all boxes that apply:

Name:

Date of birth:

Hospital No:

Consultant:

□ Medical patients with a reduction in mobility or acute illness: →	Continue full risk assessment of thrombosis and bleeding risk factors below
□ Medical patients with **normal mobility and without acute illness** (including cancer patients solely admitted for therapy) →	Thromboprophylaxis not usually required. *Risk assessment complete – sign box below*
□ Medical patients already on therapeutic anticoagulation (e.g. heparin, warfarin, dabigatran, rivaroxaban, apixaban e.t.c) *with no intention to change* →	Continue existing anticoagulation *Risk assessment complete – sign box below*

Thrombosis (VTE) Risk Factors

Patient related risk factors
- □ Active cancer or cancer treatment
- □ Age over 60 yrs
- □ Dehydration
- □ One or more significant comorbidities (e.g. heart disease; metabolic, endocrine or respiratory pathologies)
- □ Acute infection or inflammatory condition
- □ Personal history or first-degree relative with a history of VTE
- □ Obesity (BMI greater than $30kg/m^2$)
- □ Use of oestrogen-containing contraceptive therapy
- □ Use of hormone replacement therapy (HRT)
- □ Varicose veins with phlebitis
- □ Known thrombophilia
- □ Pregnant or less than 6 weeks post partum – complete *antenatal (PD7262)* or *postnatal (PD7263) risk assessment for venous thromboembolism in pregnancy* form as appropriate

Admission related risk factors
- □ Significantly reduced mobility for 3 days or more (relative to normal state)
- □ Hip or knee replacement surgery
- □ Hip fracture
- □ Total anaesthetic plus surgical time greater than 90mins
- □ Surgery involving pelvis or lower limb with a total anaesthetic plus surgical time greater than 60mins
- □ Acute surgical admission with inflammatory or intra-abdominal condition
- □ Critical care admission
- □ Surgery with significant reduction in mobility

Bleeding Risk Factors

Patient related risk factors
- □ Active bleeding
- □ Acquired bleeding
- □ Uncontrolled hypertension (greater than or equal to 230mmHg systolic or 120mmHg diastolic)
- □ Acute stroke
- □ Thrombocytopaenia (platelets less than 75 $x10^9/L$)
- □ Untreated inherited bleeding disorders (e.g. haemophilia and von Willebrand's disease)

Admission related risk factors
- □ Neurosurgery, spinal surgery or eye surgery
- □ Other procedure with high bleeding risk
- □ Lumbar puncture/ epidural/ spinal anaesthesia expected within the next 12 hours
- □ Lumbar puncture/ epidural/ spinal anaesthesia within the previous 4 hours

Action taken (for full advice see "STH Guidelines for the prevention of Venous Thromboembolic Disease")
- □ No thrombosis risk factors present so no prophylaxis needed
- □ Thrombosis risk factors present but no prophylaxis prescribed: *state reason why (e.g. already on anticoagulation therapy, bleeding risk outweighs thrombosis risk)*..

Thrombosis risk factors present: □ dalteparin prescribed
□ mechanical prophylaxis prescribed (if dalteparin contraindicated)

If thrombosis risk factors are present, prescribe dalteparin UNLESS bleeding risk outweighs VTE risk, or dalteparin is contraindicated; see below for doses:

**Patient's weight** to nearest kilogram	**eGFR greater than or equal to 20 mL/min/1.73m²**	**eGFR less than 20 mL/min/1.73m²**	**Pregnant women**
**Less than 45kg**	2500 units once daily	2500 units once daily	Refer to "*Risk assessment for VTE in pregnancy and up to 6 weeks postpartum* or "*STH Guidelines for the prevention of Venous Thromboembolic Disease*"
**45-99kg**	5000 units once daily		
**100-149kg**	7500 units once daily		
**150kg and greater**	5000 units twice a day		

8 of 12

Supplementary material: Evaluating routine data sources to determine the incidence of hospital acquired thrombosis and major bleeding in medical and surgical inpatients.

Site 4 – Electronic form within prescribing electronic health record designed to trigger consideration of VTE risk assessment at the point of hospital admission and prompt prescribing in accordance with local guidelines (St Thomas' NHS Foundation Trust).

Rules

VTE Risk Assessment

Policy Requires you to complete the VTE Risk Assessment form by selecting the form at the bottom of this message.

Instructions:

1) Complete the form
2) Click SAVE
3) Select Override
4) Click Continue

The Risk Assessment will be prescribed on the PRN tab of the medication chart indicating that a Risk Assessment has been completed for this patient. This is not a prescription.

Ensure you prescribe the appropriate thromboprophylaxis if indicated. These can be found under the VTE protocols

Rule Indices

Substance prescribed: VTE Risk Assessment

VTE Form (required): VTE Form

Action

Override Remove

Comment

Supplementary data

Figure 2: Workflow diagram

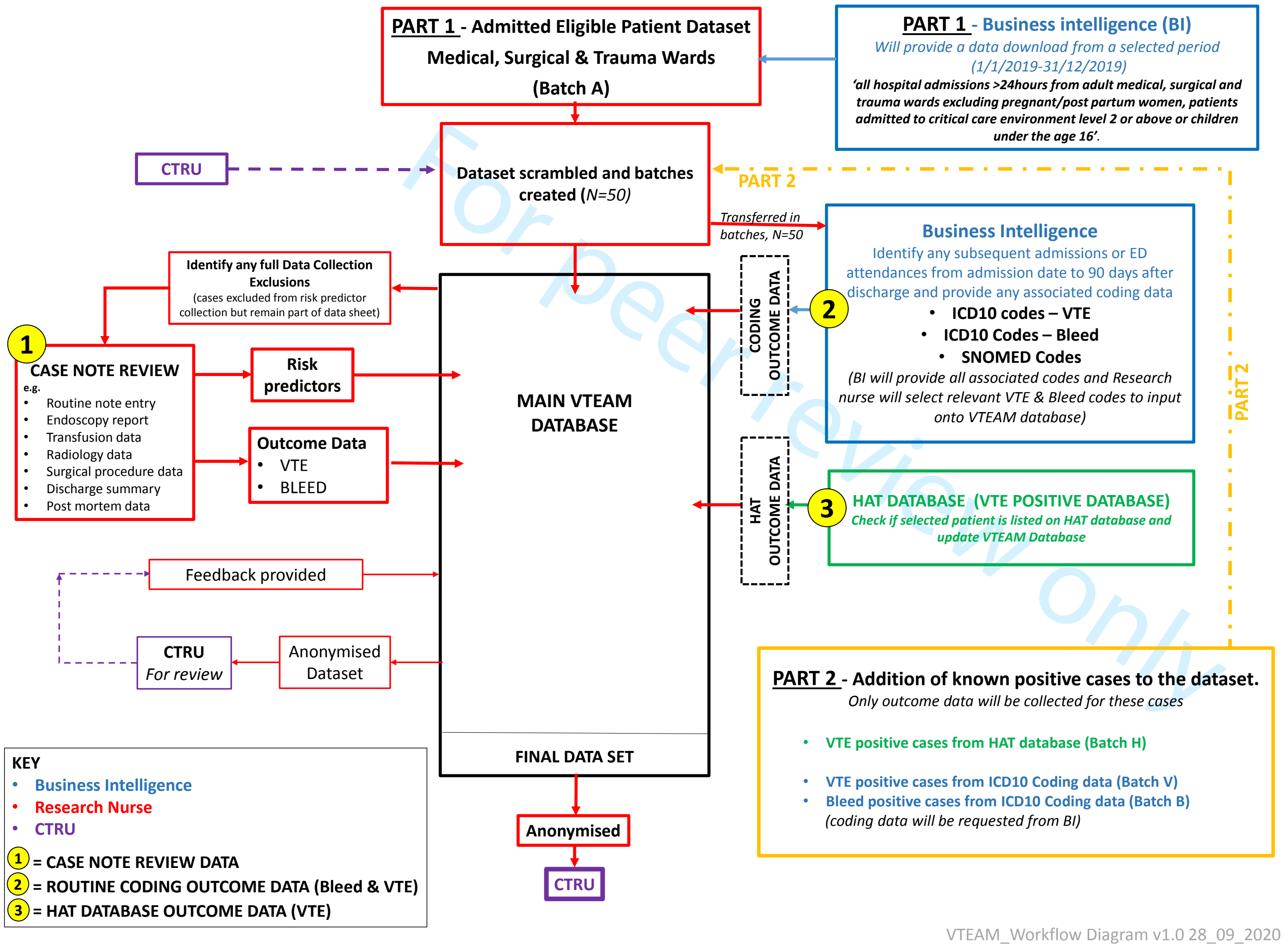

For peer review only

Supplementary data

Table 1: Relevant ICD10 codes for VTE and Bleeding agreed by chief investigators and approved by project management group

ICD10 - 4 digit	3 character description	4 character description	All diagnoses	Main diagnosis	Category	Sub-Category	Final selection
I26.0	Pulmonary embolism	Pulmonary embolism with mention of acute cor pulmonale	4031	2353	VTE		Yes
I26.9	Pulmonary embolism	Pulmonary embolism without mention of acute cor pulmonale	108637	53273	VTE		Yes
I80.1	Phlebitis and thrombophlebitis	Phlebitis and thrombophlebitis of femoral vein	10156	4294	VTE		Yes
I80.2	Phlebitis and thrombophlebitis	Phlebitis and thrombophlebitis of other deep vessels of lower extremities	61647	24297	VTE		Yes
I80.3	Phlebitis and thrombophlebitis	Phlebitis and thrombophlebitis of lower extremities, unspecified	3971	1876	VTE		Yes
I80.9	Phlebitis and thrombophlebitis	Phlebitis and thrombophlebitis of unspecified site	2906	524	VTE		Yes
I82.2	Other venous embolism and thrombosis	Embolism and thrombosis of vena cava	3891	543	VTE		Yes
I82.8	Other venous embolism and thrombosis	Embolism and thrombosis of other specified veins	10001	1870	VTE		Yes
I82.9	Other venous embolism and thrombosis	Embolism and thrombosis of unspecified vein	1124	215	VTE		Yes
I60.0	Subarachnoid haemorrhage	Subarachnoid haemorrhage from carotid siphon and bifurcation	226	212	Bleeding	intracranial bleed	Yes
I60.1	Subarachnoid haemorrhage	Subarachnoid haemorrhage from middle cerebral artery	1125	1014	Bleeding	intracranial bleed	Yes
I60.2	Subarachnoid haemorrhage	Subarachnoid haemorrhage from anterior communicating artery	1731	1599	Bleeding	intracranial bleed	Yes
I60.3	Subarachnoid haemorrhage	Subarachnoid haemorrhage from posterior communicating artery	899	838	Bleeding	intracranial bleed	Yes
I60.4	Subarachnoid haemorrhage	Subarachnoid haemorrhage from basilar artery	384	324	Bleeding	intracranial bleed	Yes
I60.5	Subarachnoid haemorrhage	Subarachnoid haemorrhage from vertebral artery	101	87	Bleeding	intracranial bleed	Yes
I60.6	Subarachnoid haemorrhage	Subarachnoid haemorrhage from other intracranial arteries	564	513	Bleeding	intracranial bleed	Yes

For peer review only

Supplementary data

I60.7	Subarachnoid haemorrhage	Subarachnoid haemorrhage from intracranial artery, unspecified	426	319	Bleeding	intracranial bleed	Yes
I60.8	Subarachnoid haemorrhage	Other subarachnoid haemorrhage	977	737	Bleeding	intracranial bleed	Yes
I60.9	Subarachnoid haemorrhage	Subarachnoid haemorrhage, unspecified	7642	4585	Bleeding	intracranial bleed	Yes
I61.0	Intracerebral haemorrhage	Intracerebral haemorrhage in hemisphere, subcortical	4996	4396	Bleeding	intracranial bleed	Yes
I61.1	Intracerebral haemorrhage	Intracerebral haemorrhage in hemisphere, cortical	5439	4254	Bleeding	intracranial bleed	Yes
I61.2	Intracerebral haemorrhage	Intracerebral haemorrhage in hemisphere, unspecified	1431	1157	Bleeding	intracranial bleed	Yes
I61.3	Intracerebral haemorrhage	Intracerebral haemorrhage in brain stem	1029	866	Bleeding	intracranial bleed	Yes
I61.4	Intracerebral haemorrhage	Intracerebral haemorrhage in cerebellum	1901	1508	Bleeding	intracranial bleed	Yes
I61.5	Intracerebral haemorrhage	Intracerebral haemorrhage, intraventricular	3678	1886	Bleeding	intracranial bleed	Yes
I61.6	Intracerebral haemorrhage	Intracerebral haemorrhage, multiple localized	764	561	Bleeding	intracranial bleed	Yes
I61.8	Intracerebral haemorrhage	Other intracerebral haemorrhage	3854	3103	Bleeding	intracranial bleed	Yes
I61.9	Intracerebral haemorrhage	Intracerebral haemorrhage, unspecified	11863	9028	Bleeding	intracranial bleed	Yes
I62.0	Other nontraumatic intracranial haemorrhage	Subdural haemorrhage (acute)(nontraumatic)	17161	8197	Bleeding	intracranial bleed	Yes
I62.1	Other nontraumatic intracranial haemorrhage	Nontraumatic extradural haemorrhage	318	100	Bleeding	intracranial bleed	Yes
I62.9	Other nontraumatic intracranial haemorrhage	Intracranial haemorrhage (nontraumatic), unspecified	3230	2383	Bleeding	intracranial bleed	Yes
I85.0	Oesophageal varices	Oesophageal varices with bleeding	4074	2876	Bleeding	gastrointestinal	Yes
K22.6	Other diseases of oesophagus	Gastro-oesophageal laceration-haemorrhage syndrome	7232	3237	Bleeding		Yes
K25.0	Gastric ulcer	Gastric ulcer - Acute with haemorrhage	2077	1469	Bleeding	gastrointestinal	Yes
K25.2	Gastric ulcer	Gastric ulcer - Acute with both haemorrhage and perforation	49	20	Bleeding	gastrointestinal	Yes

For peer review only

Supplementary data

K25.4	Gastric ulcer	Gastric ulcer - Chronic or unspecified with haemorrhage	4742	2951	Bleeding	gastrointestinal	Yes
K25.6	Gastric ulcer	Gastric ulcer - Chronic or unspecified with both haemorrhage and perforation	145	74	Bleeding	gastrointestinal	Yes
K26.0	Duodenal ulcer	Duodenal ulcer - Acute with haemorrhage	2955	2161	Bleeding	gastrointestinal	Yes
K26.2	Duodenal ulcer	Duodenal ulcer - Acute with both haemorrhage and perforation	126	96	Bleeding	gastrointestinal	Yes
K26.4	Duodenal ulcer	Duodenal ulcer - Chronic or unspecified with haemorrhage	7607	4972	Bleeding	gastrointestinal	Yes
K26.6	Duodenal ulcer	Duodenal ulcer - Chronic or unspecified with both haemorrhage and perforation	386	263	Bleeding	gastrointestinal	Yes
K27.0	Peptic ulcer, site unspecified	Peptic ulcer, site unspecified - Acute with haemorrhage	78	32	Bleeding	gastrointestinal	Yes
K27.2	Peptic ulcer, site unspecified	Peptic ulcer, site unspecified - Acute with both haemorrhage and perforation	4	1	Bleeding	gastrointestinal	Yes
K27.4	Peptic ulcer, site unspecified	Peptic ulcer, site unspecified - Chronic or unspecified with haemorrhage	231	116	Bleeding	gastrointestinal	Yes
K27.6	Peptic ulcer, site unspecified	Peptic ulcer, site unspecified - Chronic or unspecified with both haemorrhage and perforation	29	9	Bleeding	gastrointestinal	Yes
K28.0	Gastrojejunal ulcer	Gastrojejunal ulcer - Acute with haemorrhage	29	24	Bleeding	gastrointestinal	Yes
K28.2	Gastrojejunal ulcer	Gastrojejunal ulcer - Acute with both haemorrhage and perforation	3	3	Bleeding	gastrointestinal	Yes
K28.4	Gastrojejunal ulcer	Gastrojejunal ulcer - Chronic or unspecified with haemorrhage	149	96	Bleeding	gastrointestinal	Yes
K28.6	Gastrojejunal ulcer	Gastrojejunal ulcer - Chronic or unspecified with both haemorrhage and perforation	11	6	Bleeding	gastrointestinal	Yes
K29.0	Gastritis and duodenitis	Acute haemorrhagic gastritis	3340	1365	Bleeding	gastrointestinal	Yes
K62.5	Other diseases of anus and rectum	Haemorrhage of anus and rectum	37545	21106	Bleeding	gastrointestinal	Yes
K66.1	Other disorders of peritoneum	Haemoperitoneum	3317	642	Bleeding	gastrointestinal	Yes
K92.0	Other diseases of digestive system	Haematemesis	67589	27503	Bleeding	gastrointestinal	Yes
K92.1	Other diseases of digestive system	Melaena	67036	22979	Bleeding	gastrointestinal	Yes
K92.2	Other diseases of digestive system	Gastrointestinal haemorrhage, unspecified	192053	97428	Bleeding	gastrointestinal	Yes

For peer review only

Supplementary data

M25.0	Other joint disorders, not elsewhere classified	Haemarthrosis	3362	1730	Bleeding		Yes
N02.0	Recurrent and persistent haematuria	Recurrent and persistent haematuria - Minor glomerular abnormality	48	21	Bleeding		Yes
N02.1	Recurrent and persistent haematuria	Recurrent and persistent haematuria - Focal and segmental glomerular lesions	286	76	Bleeding		Yes
N02.2	Recurrent and persistent haematuria	Recurrent and persistent haematuria - Diffuse membranous glomerulonephritis	1858	517	Bleeding		Yes
N02.3	Recurrent and persistent haematuria	Recurrent and persistent haematuria - Diffuse mesangial proliferative glomerulonephritis	160	49	Bleeding		Yes
N02.4	Recurrent and persistent haematuria	Recurrent and persistent haematuria - Diffuse endocapillary proliferative glomerulonephritis	10	4	Bleeding		Yes
N02.5	Recurrent and persistent haematuria	Recurrent and persistent haematuria - Diffuse mesangiocapillary glomerulonephritis	47	12	Bleeding		Yes
N02.6	Recurrent and persistent haematuria	Recurrent and persistent haematuria - Dense deposit disease	9	1	Bleeding		Yes
N02.7	Recurrent and persistent haematuria	Recurrent and persistent haematuria - Diffuse crescentic glomerulonephritis	164	48	Bleeding		Yes
N02.8	Recurrent and persistent haematuria	Recurrent and persistent haematuria - Other	10852	1289	Bleeding		Yes
N02.9	Recurrent and persistent haematuria	Recurrent and persistent haematuria - Unspecified	2902	1331	Bleeding		Yes
N93.8	Other abnormal uterine and vaginal bleeding	Other specified abnormal uterine and vaginal bleeding	4801	2899	Bleeding	other bleed	Yes
N93.9	Other abnormal uterine and vaginal bleeding	Abnormal uterine and vaginal bleeding, unspecified	24423	11036	Bleeding	other bleed	Yes
R04.0	Haemorrhage from respiratory passages	Epistaxis	48741	24610	Bleeding	other bleed	Yes
R04.1	Haemorrhage from respiratory passages	Haemorrhage from throat	191	69	Bleeding	other bleed	Yes
R04.2	Haemorrhage from respiratory passages	Haemoptysis	32143	12743	Bleeding	other bleed	Yes
R04.8	Haemorrhage from respiratory passages	Haemorrhage from other sites in respiratory passages	1332	222	Bleeding	other bleed	Yes
R04.9	Haemorrhage from respiratory passages	Haemorrhage from respiratory passages, unspecified	83	23	Bleeding	other bleed	Yes

For peer review only

Supplementary data

R23.3	Other skin changes	Spontaneous ecchymoses	10624	2774	Bleeding		Yes
R58.X	Haemorrhage, not elsewhere classified	Haemorrhage, not elsewhere classified	2747	408	Bleeding		Yes
T81.0	Complications of procedures, not elsewhere classified	Haemorrhage and haematoma complicating a procedure, not elsewhere classified	67338	28601	Bleeding		Yes
T81.7	Complications of procedures, not elsewhere classified	Vascular complications following a procedure, not elsewhere classified	1000	201	Bleeding		Yes

For peer review only

STROBE Statement—Checklist of items that should be included in reports of ***cohort studies***

	Item No	Recommendation	Page No
**Title and abstract**	1	(*a*) Indicate the study's design with a commonly used term in the title or the abstract	1
		(*b*) Provide in the abstract an informative and balanced summary of what was done and what was found	3
**Introduction**			
Background/rationale	2	Explain the scientific background and rationale for the investigation being reported	5
Objectives	3	State specific objectives, including any prespecified hypotheses	5
**Methods**			
Study design	4	Present key elements of study design early in the paper	6-7
Setting	5	Describe the setting, locations, and relevant dates, including periods of recruitment, exposure, follow-up, and data collection	6-7
Participants	6	(*a*) Give the eligibility criteria, and the sources and methods of selection of participants. Describe methods of follow-up	6-7
		(*b*) For matched studies, give matching criteria and number of exposed and unexposed	
Variables	7	Clearly define all outcomes, exposures, predictors, potential confounders, and effect modifiers. Give diagnostic criteria, if applicable	8
Data sources/ measurement	8*	For each variable of interest, give sources of data and details of methods of assessment (measurement). Describe comparability of assessment methods if there is more than one group	7-8
Bias	9	Describe any efforts to address potential sources of bias	8
Study size	10	Explain how the study size was arrived at	8-9
Quantitative variables	11	Explain how quantitative variables were handled in the analyses. If applicable, describe which groupings were chosen and why	8
Statistical methods	12	(*a*) Describe all statistical methods, including those used to control for confounding	8
		(*b*) Describe any methods used to examine subgroups and interactions	
		(*c*) Explain how missing data were addressed	
		(*d*) If applicable, explain how loss to follow-up was addressed	
		(*e*) Describe any sensitivity analyses	
**Results**			
Participants	13*	(a) Report numbers of individuals at each stage of study—eg numbers potentially eligible, examined for eligibility, confirmed eligible, included in the study, completing follow-up, and analysed	11
		(b) Give reasons for non-participation at each stage	
		(c) Consider use of a flow diagram	
Descriptive data	14*	(a) Give characteristics of study participants (eg demographic, clinical, social) and information on exposures and potential confounders	Table 1&2, 11
		(b) Indicate number of participants with missing data for each variable of interest	
		(c) Summarise follow-up time (eg, average and total amount)	
Outcome data	15*	Report numbers of outcome events or summary measures over time	11

For peer review only - http://bmjopen.bmj.com/site/about/guidelines.xhtml
For peer review only

Main results	16	(*a*) Give unadjusted estimates and, if applicable, confounder-adjusted estimates and their precision (eg, 95% confidence interval). Make clear which confounders were adjusted for and why they were included<br>(*b*) Report category boundaries when continuous variables were categorized<br>(*c*) If relevant, consider translating estimates of relative risk into absolute risk for a meaningful time period	11
Other analyses	17	Report other analyses done—eg analyses of subgroups and interactions, and sensitivity analyses	12
**Discussion**			
Key results	18	Summarise key results with reference to study objectives	13
Limitations	19	Discuss limitations of the study, taking into account sources of potential bias or imprecision. Discuss both direction and magnitude of any potential bias	13-14
Interpretation	20	Give a cautious overall interpretation of results considering objectives, limitations, multiplicity of analyses, results from similar studies, and other relevant evidence	15-16
Generalisability	21	Discuss the generalisability (external validity) of the study results	16
**Other information**			
Funding	22	Give the source of funding and the role of the funders for the present study and, if applicable, for the original study on which the present article is based	2

*Give information separately for exposed and unexposed groups.

Note: An Explanation and Elaboration article discusses each checklist item and gives methodological background and published examples of transparent reporting. The STROBE checklist is best used in conjunction with this article (freely available on the Web sites of PLoS Medicine at http://www.plosmedicine.org/, Annals of Internal Medicine at http://www.annals.org/, and Epidemiology at http://www.epidem.com/). Information on the STROBE Initiative is available at http://www.strobe-statement.org.

For peer review only

BMJ Open

The accuracy of efficient data methods to determine the incidence of hospital acquired thrombosis and major bleeding in medical and surgical inpatients: a multicentre observational cohort study in four UK hospitals

Journal:	*BMJ Open*
Manuscript ID	bmjopen-2022-069244.R1
Article Type:	Original research
Date Submitted by the Author:	02-Jan-2023
Complete List of Authors:	Horner, Daniel; Northern Care Alliance NHS Foundation Trust, Emergency Department; The University of Manchester, Division of Infection, Immunity and Respiratory Medicine<br>Rex, Saleema; The University of Sheffield, School of Health and Related Research (ScHARR)<br>Reynard, Charles; The University of Manchester, Division of Cardiovascular sciences<br>Bursnall, Matthew; The University of Sheffield, School of Health and Related Research (ScHARR)<br>Bradburn, Mike; University of Sheffield, ScHARR<br>de Wit, Kerstin; McMaster University Department of Medicine, Department of Medicine; Hamilton General Hospital<br>Goodacre, Steve; University of Sheffield, Medical Care Research Unit<br>Hunt, Beverley; Guy's and St Thomas' NHS Foundation Trust, Kings Healthcare Partners & Thrombosis & Haemophilia Centre
**Primary Subject Heading**:	Haematology (incl blood transfusion)
Secondary Subject Heading:	Cardiovascular medicine, Diagnostics, Health informatics
Keywords:	Anticoagulation < HAEMATOLOGY, Thromboembolism < CARDIOLOGY, Risk management < HEALTH SERVICES ADMINISTRATION & MANAGEMENT

SCHOLARONE™
Manuscripts

I, the Submitting Author has the right to grant and does grant on behalf of all authors of the Work (as defined in the below author licence), an exclusive licence and/or a non-exclusive licence for contributions from authors who are: i) UK Crown employees; ii) where BMJ has agreed a CC-BY licence shall apply, and/or iii) in accordance with the terms applicable for US Federal Government officers or employees acting as part of their official duties; on a worldwide, perpetual, irrevocable, royalty-free basis to BMJ Publishing Group Ltd ("BMJ") its licensees and where the relevant Journal is co-owned by BMJ to the co-owners of the Journal, to publish the Work in this journal and any other BMJ products and to exploit all rights, as set out in our licence.

The Submitting Author accepts and understands that any supply made under these terms is made by BMJ to the Submitting Author unless you are acting as an employee on behalf of your employer or a postgraduate student of an affiliated institution which is paying any applicable article publishing charge ("APC") for Open Access articles. Where the Submitting Author wishes to make the Work available on an Open Access basis (and intends to pay the relevant APC), the terms of reuse of such Open Access shall be governed by a Creative Commons licence – details of these licences and which Creative Commons licence will apply to this Work are set out in our licence referred to above.

Other than as permitted in any relevant BMJ Author's Self Archiving Policies, I confirm this Work has not been accepted for publication elsewhere, is not being considered for publication elsewhere and does not duplicate material already published. I confirm all authors consent to publication of this Work and authorise the granting of this licence.

For peer review only

Title:

The accuracy of efficient data methods to determine the incidence of hospital acquired thrombosis and major bleeding in medical and surgical inpatients: a multicentre observational cohort study in four UK hospitals

Author names and positions:

Daniel Horner [1,2]	Consultant in Emergency and Intensive Care Medicine
Saleema Rex[3]	Research Associate
Charlie Reynard [4]	Postgraduate Research Fellow
Matt Bursnall [3]	Research Associate
Mike Bradburn [3]	Senior Medical Statistician
Kerstin de Wit [5]	Associate Professor
Steve Goodacre [3]	Professor of Emergency Medicine
Beverley J. Hunt [6]	Professor of Thrombosis and Haemostasis

Affiliations:

[1] Emergency Department, Northern Care Alliance NHS Foundation Trust, Salford, UK
[2] Division of Infection, Immunity and Respiratory Medicine, University of Manchester, Oxford Road, UK
[3] Centre for Urgent and Emergency Care Research (CURE), University of Sheffield, Sheffield, UK
[4] Division of Cardiovascular Sciences, University of Manchester, Oxford Road, Manchester, UK
[5] Department of Medicine, McMaster University, Hamilton, Ontario, Canada.
[6] Kings Healthcare Partners & Thrombosis & Haemophilia Centre, Guy's & St Thomas' NHS Foundation Trust, London, UK

Corresponding author:

Daniel Horner daniel.horner@nca.nhs.uk
Mailing address: Emergency Department, Northern Care Alliance NHS Foundation Trust, Stott Lane, Salford, M6 8HD

Word Counts:

Abstract word count: 246
Total word count: 3870
References: 23

Notation of prior abstract publication/presentation:

Sections of this work were presented at the ISTH 2022 conference in London by poster. This work is also accepted for publication by the NIHR in monograph format within their journals library in April 2023. The NIHR strongly encourages wide dissemination of funded work and early submission of manuscripts, as per the embargo policy:

For peer review only - http://bmjopen.bmj.com/site/about/guidelines.xhtml

"In line with our embargo policy, we are prepared to delay publication of your Journals Library manuscript and, where possible, co-ordinate publication of your Journals Library manuscript with any journal articles."

More details on the NIHR Embargo policy can be found at the following address: https://www.journalslibrary.nihr.ac.uk/policies/#Embargo

Keywords:

Venous Thromboembolism

Risk Assessment

Thromboprophylaxis

Routine data

Efficient methods

For peer review only

Abstract

Objectives:

We evaluated the accuracy of using routine health service data to identify hospital acquired thrombosis (HAT) and major bleeding events (MBE) compared to a reference standard of case note review.

Design:

A multi-centre observational cohort study.

Setting:

Four acute hospitals in the United Kingdom.

Participants:

A consecutive unselective cohort of general medical and surgical patients requiring hospitalisation for a period of >24h during the calendar year 2021. We excluded paediatric, obstetric and critical care patients due to differential risk profiles.

Interventions:

We compared routinely collected information using hospital coding data and local contractually mandated thrombosis datasets, to data extracted from case notes using a predesigned workflow methodology.

Primary and Secondary Outcome Measures:

We defined HAT as objectively confirmed venous thromboembolism occurring during hospital stay or within 90 days of discharge and MBE as per international consensus.

Results:

We were able to collect the necessary routine data from 87% of 2008 case episodes examined. The sensitivity of hospital coding data (ICD-10) for the diagnosis of HAT and MBE was 62% (95% CI 54 to 69) and 38% (95% CI 27 to 50) respectively. Sensitivity improved to 81% (95% CI 75 to 87) when using local thrombosis databsets.

Conclusions:

Using routine data instead of case note review will miss a substantial proportion of outcome events. Our study suggests that currently available routine data collection methods in the UK are inadequate to support efficient study designs in venous thromboembolism research.

Trial registration:

This project is registered at https://fundingawards.nihr.ac.uk/award/NIHR127454

For peer review only - http://bmjopen.bmj.com/site/about/guidelines.xhtml

Article Summary:

Strengths and Limitations of this study

- This observational cohort study recruited over 2000 patients across four NHS trusts to evaluate the accuracy of routine data to identify hospital acquired thrombosis and major bleeding events, compared to case note review.
- We worked with patients, clinical experts and researchers to develop a detailed workflow pathway and iterative dataset, conforming to international outcome definitions.
- Data abstractors were blinded to routine data sources throughout extraction, limiting bias in case ascertainment.
- We only evaluated general medical and surgical hospital admissions; we did not assess the accuracy of routine data for outcomes in complex cohorts such as cancer or neurosurgical patients.
- We only evaluated routinely collected and available data among four large urban hospitals and in a UK healthcare setting. Our findings may therefore lack generalisability to other settings.

For peer review only - http://bmjopen.bmj.com/site/about/guidelines.xhtml

For peer review only

BACKGROUND

Venous Thromboembolism (VTE) remains a major global health burden, with significant attributable morbidity and mortality.[1] At least half of all VTE occurs during hospitalisation, or up to 90 days following discharge; such cases are described as Hospital Acquired Thrombosis (HAT).[2] Many of these events are potentially preventable through patient education and provision of thromboprophylaxis to those at risk.

Research into thromboprophylaxis often requires large sample sizes to identify small but important differences in clinically relevant events, such as HAT and/or major bleeding. Study protocols will often necessitate examination of case notes to identify outcome events, which can be time consuming and expensive. This is particularly relevant for external validation of new clinical decision rules or risk assessment models which aim to guide prescribing of thromboprophylaxis for hospital inpatients.[3-5]

Using routine health service data to identify outcome events could markedly improve the efficiency of research and facilitate studies with large sample sizes at acceptable cost. However, this approach requires confirmatory evidence that routine data sources accurately identify outcome events.

Several mechanisms already exist for routine identification of outcomes, including hospital coding, local VTE datasets, and pathology reporting (with thrombosis committee oversight). If such efficient methods could accurately ascertain relevant outcomes, large scale studies would be theoretically deliverable.

We sought to evaluate the accuracy of using routine data to identify HAT and major bleeding events (MBE) compared to case note examination.

For peer review only - http://bmjopen.bmj.com/site/about/guidelines.xhtml

METHODS

We conducted a multi-centre observational cohort study within the context of a wider project examining the overall clinical and cost effectiveness of VTE risk assessment models.[6] The aim of this study was to estimate the accuracy and completeness of available coding data and local registry data to determine clinically relevant VTE and bleeding outcomes against case note review by trained research assistants.

We approached four sites to participate in this study; the Northern Care Alliance NHS Foundation Trust, Manchester University Hospitals NHS Foundation Trust, the Northern General Hospital in Sheffield and Guy's & St Thomas' NHS Foundation Trust.

Study Population

We identified a consecutive, unselected cohort of general medical and surgical patients requiring hospital admission at each site during the calendar year 1st January to 31st December 2019. We chose 2019 because of concern that patients admitted during the subsequent COVID-19 pandemic might represent an atypical cohort. We collated data on all risk assessments that clinical staff performed prospectively at the point of hospital admission, then scrambled episodes into randomly assorted batches of 50 (A batches) to ensure diversity in specialty presentation and mitigate seasonal bias. We collated in batches of 50 to facilitate iterative and incremental case ascertainment; initial hospital downloads were often in excess of 50,000 case episodes. In order to keep workflow manageable and organized, we worked through batches of 50 and reported routinely to a steering committee, who provided guidance on study delivery. We excluded paediatric patients (age <16), anyone requiring critical care admission (defined as level 2 care or above) and pregnant/post-partum patients due to differential VTE and bleeding risks, as outlined in the wider study protocol.[7]

Study design

For each patient episode, we extracted baseline demographics and prospectively collected data on VTE risk assessment (where available) from the electronic health record, with support from business intelligence teams. Risk assessments were captured differently at each site, including the use of a paper proforma, dichotomous output on

For peer review only - http://bmjopen.bmj.com/site/about/guidelines.xhtml

electronic prescribing (low/high risk), or through a detailed structured note within the electronic healthcare record. Example images/screenshots for each site can be found in the supplementary appendix. All four sites used the Department of Health tool to facilitate VTE risk assessment; this Risk Assessment Model (RAM) has been developed by expert consensus and is recommended in national UK guidance.[8] Recent survey data suggests the tool is used by >80% of NHS sites, despite limited available data on external validation.[9] This tool confers a high rate of prescribing in comparison with other RAMs, as highlighted in a recent practice review.[3]

Research assistants at each site undertook retrospective case note review for each patient episode through shared primary and secondary care electronic health records (EHR). We utilised EHR to access primary care data on hospital attendance, diagnoses and investigation within the relevant time periods. Primary care EHR systems varied by trust. We used secondary care EHR to identify hospital reattendance, investigations, diagnostic imaging and confirmed diagnoses (via discharge summary or note entry). Secondary care EHR systems varied by trust, but access to radiology investigations was universal within the patient archiving and communication system (PACS). We extracted descriptive data on relevant clinical outcomes such as the subsequent diagnosis of VTE/HAT, major bleeding and clinically relevant non-major bleeding events as per internationally agreed definitions.[10 11] We defined VTE as any pulmonary embolism or deep vein thrombosis identified in routine care by the treating clinical team, in accordance with ISTH common data elements.[12] Superficial venous thrombosis was specifically excluded from this definition. We defined HAT in accordance with the definition proposed by NHS England (any new episode of VTE occurring during hospitalisation or within 90 days of discharge, following an inpatient stay of ≥2 days or a surgical procedure under general/regional anaesthesia).[13] Data extractors were trained in identification of these outcomes and followed a detailed workflow diagram (supplementary material). Data abstractors were blinded to batch allocation, final ICD-10 coding, local database entries and the final analysis plan.

Following case note review, we collected data elements from multiple data sources for each patient episode to evaluate their combined accuracy against case note review outcomes. Data sources for interrogation were identified a-priori and included the

For peer review only - http://bmjopen.bmj.com/site/about/guidelines.xhtml

following; ICD-10 diagnostic codes judged relevant to thrombosis or bleeding by the project management group (a-priori, shown in the supplementary data); Emergency Care Data Set/SNOMED codes relevant to thrombosis or bleeding; and contractual local Hospital Acquired Thrombosis (HAT) databases. HAT database entries are co-ordinated by local thrombosis committees in accordance with NHS contract standards and include a contemporary register of all patients diagnosed with acute VTE at the hospital site, informed by radiology or identified by pathology at post mortem.[13] All cases are subsequently categorised by the local thrombosis committee as either de-novo VTE or HAT based on case review, local expert opinion and data on any preceding hospital admission (up to 90 days) or VTE diagnosis >24h following hospital admission. This database is maintained contemporaneously and provides an ongoing opportunity for hospitals to identify preventable HAT and conduct root cause analysis (RCA) for each episode, to promote learning and best practice. All data sources were interrogated for the duration of hospital stay and up to 90 days post discharge, for each patient episode. Data sources were obtained through routine local business intelligence requests or direct approach to local coding teams. HAT database entries were obtained where feasible through local site thrombosis committee chairs.

Given the potential for negligible VTE/MBE in the wider study population (leading to limited information on the accuracy of efficient data methods), we augmented the overall sample with positive thrombosis and bleeding cases. We obtained positive cases through ICD-10 coding identification for VTE events (V batches), bleeding events (B batches) and positive VTE cases from local HAT database entries (H batches), identified as above and sourced from local thrombosis committee leads. Positive cases were batched and reviewed in accordance with the general study protocol. Data extractors were blinded to batch allocation.

Outcomes

The following criteria were proposed to determine whether routine data identify outcome events with sufficient accuracy to support efficient methods:

1. Proportion of outcome events identified by routine data sources that are confirmed by record review (target 100%)

2. Proportion of cases with no outcome event identified by routine data sources that have an event identified on record review (target 0%)

For peer review only - http://bmjopen.bmj.com/site/about/guidelines.xhtml

3. Proportion of inpatients with data collected (target 90%)

Statistical analysis

The accuracy of routinely recorded HAT and bleeding events was compared against direct case note review data for the cohort. Case note review determination of events was assumed to be the gold standard. Data are presented in contingency tables with sensitivity, specificity, positive and negative predictive values along with confidence intervals calculated using the Wilson score method.[14]

The primary analysis included patients identified from all sources (A, V, B or H batches) with bleeding and HAT assumed to have not occurred unless coded as such in the relevant data, or detected following case note review. In addition, two pre-planned sensitivity analyses were undertaken:

a. Inclusion was limited to cases identified in routine case review (i.e. "A batch patients" only), with exclusion of all augmented sample cases.
b. Inclusion limited to participants for whom bleeding or HAT was definitively recorded

We took a conservative approach and interpreted missing or unknown endpoints as 'no event' with the exception of the second sensitivity analysis.

We originally planned to identify 3000 inpatients across 4 hospitals during a 12-month study period within the 2-year project plan, dependent on appointment of research assistants and time required for outcome ascertainment. This sample size was designed to allow key parameters to be estimated with a high degree of precision across the whole cohort (standard error <1%). All sites failed to meet their sample target of 750 for reasons mainly related to the SARs COV-2 pandemic, including redeployment of research staff to clinical care, delayed local approvals secondary to prioritised pandemic research and a longer than anticipated time for individual outcome ascertainment per case review

Ethical Aspects

The study received a favourable opinion from the Proportionate Review Sub-committee of the London - West London & GTAC Research Ethics Committee and approval from

For peer review only - http://bmjopen.bmj.com/site/about/guidelines.xhtml

the HRA and Care Research Wales (HCRW) on 18th September 2019 (reference 19/LO/1303, IRAS project ID 262220).

Participating sites identified members of the clinical care team (research nurses or assistants predominately) to access patient records and extract clinical data using a predesigned and protected Microsoft Excel© database with embedded macro function, hosted at site. All data subsequently underwent local de-identification following completion and were exported to an independent team of statisticians at the Clinical Trials Research Unit (CTRU) in Sheffield, for collation and analysis.

All aspects of the data collection process, export, analysis and oversight were regularly reviewed by the internal Project Management Group (PMG) including CTRU representation, and an external Trial Steering Committee (TSC), throughout the duration of the project.

We conducted this study in accordance with international EQUATOR guidelines. A STROBE reporting checklist was used throughout to inform design, conduct and analysis of this observational cohort study and is included as supplementary information.

Patient and Public Involvement

Representatives of two Patient and Public Involvement groups (PPI), thrombosis UK and Sheffield Emergency Care Forum joined the research team and were involved in developing the initial proposal and undertaking the wider study.

The Sheffield Emergency Care Forum (SECF) is a patient and public representative group with an interest in emergency care research. The forum has provided PPI for many emergency care research projects over then last ten years.[15] Thrombosis UK is a charity that aims to identify, inform and partner the NHS, healthcare providers and individuals to work to improve prevention of VTE and the management and care of VTE events (see https://www.thrombosisuk.org/).

For peer review only - http://bmjopen.bmj.com/site/about/guidelines.xhtml

The PPI members were involved in determining the study design and ensuring that the proposal addressed the needs of patients and the NHS, while respecting the needs of potential participants. Their input regarding the importance of providing thromboprophylaxis for potential participants of any prospective cohort study and the need for such a study to yield reliable findings was instrumental in determining our approach to answering the research question. The PPI members also provided input at project management meetings and, where required, in day-to-day running of the project. The members used meetings and surveys of their wider PPI groups to enhance PPI in the project.

For peer review only

For peer review only - http://bmjopen.bmj.com/site/about/guidelines.xhtml

RESULTS

We identified 2115 patients with an original hospital admission occurring in the calendar year 2019. Of these, 107 patient episodes were ineligible due to being pregnant or post-partum women (n=49); admitted to a critical care environment of level 2 or above (n=38); children aged under 16 (n=13) or for unrecorded reasons (n=7) leaving 2008 episodes for analysis. All episodes were suitable for data extraction and comparison to routine data sources.

Patient episodes showed an even balance of medical and surgical cases, but with a focus on emergency (73.7%) rather than elective (25.8%) admissions. A broad range of subspecialty interests were represented within the cohort. Median length of stay was three days (IQR 3 to 8) and mean length of stay 7.75 days (SD 16.5). Specialty groups with frequencies and cumulative percentages are shown in Table 1. The vast majority of patient episodes (1809, 90.1%) were taken from 'A' batches. The total sample was augmented by 45 patients (2.2%) with potential bleeding events and 154 (7.7%) patients with potential VTE events. All sites contributed evenly to the sample with one exception; reduced numbers at this site reflect a delay to institutional approval during the pandemic, arising from a high burden of other clinical research studies and high staff turnover. Site and batch numbers are shown in Table 2

Main Findings

Contingency tables for the accuracy of routine data sources compared to case note review for both HAT and major bleeding events are shown in Table 3. Sensitivity was 62% (95% CI 54 to 69) for the use of ICD-10/SNOMED coding data to detect HAT events and 81% (95% CI 75 to 87) for local HAT database entries. Sensitivity by individual site ranged from 45% (95% CI, 28 to 63) to 72% (95% CI, 61 to 82) using ICD-10/SNOMED coding and 68% (95% CI, 51 to 84) to 94% (95% CI 87 to 100) using local HAT database entries.

The sensitivity of ICD10/SNOMED coding to detect major bleeding events identified by case note review was 38% (95% CI 27 to 50). Sensitivity by individual site ranged from 22% (95% CI 0 to 49) to 56% (95% CI 37 to 75).

For peer review only - http://bmjopen.bmj.com/site/about/guidelines.xhtml

Table 1: Clinical category and admission type, with frequency and cumulative percentage

	Frequency	Percentage	Cumulative
**Admission type**			
Missing	1	0.05	0.05
Elective	518	25.80	25.85
Emergency	1,480	73.71	99.55
Unknown	9	0.45	100
Total	2,008	100	
**Specialty Group**			
Missing	9	0.45	0.45
Medical	902	44.92	45.37
Surgical	951	47.36	92.73
Tertiary specialty	146	7.27	100
Total	2,008	100	
**Clinical Category**			
Missing	9	0.45	0.45
Acute medicine	340	16.93	17.38
Ageing and complex medicine	133	6.62	24.0
Cardiology	41	2.04	26.04
Cardiothoracic Surgery	87	4.33	30.38
Dermatology	2	0.1	30.48
Emergency Medicine	87	4.33	34.81
Gastroenterology	61	3.04	37.85
General Surgery	285	14.19	52.04
Medical: Other	169	8.42	60.46
Neurology	10	0.5	60.96
Neurorehabilitation	2	0.1	61.06
Neurosurgery	39	1.94	63.0
Gynaecology	57	2.84	65.84
Renal Medicine	26	1.29	67.13
Respiratory	63	3.14	70.27
Rheumatology	2	0.1	70.37
Trauma and Orthopaedics	158	7.87	78.24
Upper GI Surgery	13	0.65	78.89
Urology	107	5.33	84.22
Surgery: Other	170	8.47	92.69
Tertiary specialty: Other	147	7.32	100
Total	2,008	100	

For peer review only - http://bmjopen.bmj.com/site/about/guidelines.xhtml

Table 2: Number of cases submitted by site and batch type

A – Patient admissions requiring routine risk assessment
B – Potential cases of bleeding (selected from relevant ICD-10 codes)
H – Cases of Hospital Acquired Thrombosis (HAT) identified through local thrombosis committee infrastructure
V - Potential cases of venous thromboembolic disease (selected from relevant ICD-10 codes)

	Batch				Total
	A	B	H	V	
GSTT	504	0	21	0	525
Manchester	241	0	0	0	241
Salford	570	45	44	46	705
Sheffield	494	0	43	0	537
Total	1809	45	108	46	2008

For peer review only

Table 3: Contingency tables for main outcomes

HAT = Hospital Acquired Thrombosis
*Manchester site excluded from this analysis as unable to access local HAT database.

		HAT from case note review		
		Yes	No	
**HAT from ICD10/SNOMED codes**	Yes	95	39	71% (63%, 79%) True positive rate and 95% CI
	No	59	1815	3% (2%, 4%) False negative rate and 95% CI
		62% (54%, 69%) Sensitivity and 95% CI	98% (97%, 99%) Specificity and 95% CI	(N=2008)
		Yes	No	
**HAT from HAT RCA database**	Yes	122	0	100% (100%, 100%) True positive rate and 95% CI
	No	29	1616	2% (1%, 2%) False negative rate and 95% CI
		81% (75%, 87%) Sensitivity and 95% CI	100% (100%, 100%) Specificity and 95% CI	(N=1767)*
		**Major bleed from case note review**		
		Yes	No	
**Major Bleed from ICD10/SNOMED codes**	Yes	25	98	20% (13%, 27%) True positive rate and 95% CI
	No	40	1845	2% (1%, 3%) False negative rate and 95% CI
		38% (27%, 50%) Sensitivity and 95% CI	95% (94%, 96%) Specificity and 95% CI	(N=2008)

For peer review only - http://bmjopen.bmj.com/site/about/guidelines.xhtml

Pre-planned Sensitivity Analysis

A sensitivity analysis was conducted using only patient episodes obtained through 'A' batches, to remove augmentation of the sample and mitigate bias. The sensitivity of efficient data methods to detect key outcomes identified at case note review remained poor. These results are summarised in Table 4.

We found the HAT event rate on case note review to be 29/1809 (1.6%, 95% CI 1.0 to 2.2) and the major bleeding event rate to be 45/1809 (2.5%, 95% CI 1.8 to 3.2) within this large cohort of hospitalised patients receiving risk assessment and thromboprophylaxis in the context of routine care.

The proportion of outcome HAT events identified by routine data sources that were confirmed by record review (target 100%) was 71% (95% CI 63 to 79) for ICD-10/SNOMED coding and 100% (95% CI 97 to 100) for local HAT database entries. The proportion of cases with no HAT outcome event identified by routine data sources that had an event identified on record review (target 0%) was 3% (95% CI 2 to 4) for ICD-10/SNOMED coding and 2% (95% CI 1 to 2) for local HAT database entries. The proportion of major bleeding events identified by routine data sources that were confirmed by record review (target 100%) was 20% (95% CI 13 to 27) for ICD-10/SNOMED coding. The proportion of cases with no major bleeding outcome event identified by routine data sources that have an event identified on record review (target 0%) was 2% (95% CI 1 to 3) for ICD-10/SNOMED coding. We were able to collect outcome data for 1745/2008 (87%) inpatients (target 90%). This was <100% due to difficulty accessing the local HAT database at a single site. Excluding this issue, the other three sites all managed to collect relevant outcome data for at least 98% of patients.

For peer review only - http://bmjopen.bmj.com/site/about/guidelines.xhtml

Table 4: Sensitivity analysis using only A batch cases. N=1809 following removal of H/B/V batch patients. *Manchester unable to access HAT database

		**HAT from case note review**		
		Yes	No	
**HAT from ICD10/SNOMED codes**	Yes	18	18	50% (34%, 66%) True positive rate and 95% CI
	No	11	1762	1% (0%, 1%) False negative rate and 95% CI
		62% (44%, 80%) Sensitivity and 95% CI	99% (99%, 99%) Specificity and 95% CI	(N=1809)
		Yes	No	
**HAT from HAT RCA database**	Yes	7	0	100% (100%, 100%) True positive rate and 95% CI
	No	19	1542	1% (1%, 2%) False negative rate and 95% CI
		27% (10%, 44%) Sensitivity and 95% CI	100% (100%, 100%) Specificity and 95% CI	(N=1568)*
		**Major bleed from case note review**		
		Yes	No	
**Bleed from ICD10/SNOMED codes**	Yes	14	68	17% (9%, 25%) True positive rate and 95% CI
	No	31	1696	2% (1%, 2%) False negative rate and 95% CI
		31% (18%, 45%) Sensitivity and 95% CI	96% (95%, 97%) Specificity and 95% CI	(N=1809)

For peer review only - http://bmjopen.bmj.com/site/about/guidelines.xhtml

DISCUSSION

Statement of principal findings

Our findings suggest that using currently available routine data for identification of HAT and MBE during hospital admission or within 90 days of discharge is not sufficiently sensitive to support a large data-enabled study. We failed to demonstrate feasibility for a number of predefined metrics and conclude that use of routine data to identify outcomes would be highly likely to miss important events, and may erroneously identify false positive events.

Strengths and weaknesses of the study

We engaged a combination of digitally mature and paper-based UK NHS sites in this study, used strict consensus definitions for VTE/bleeding events and evaluated only predefined efficient data sources. We also used topic experts and research staff to iteratively develop our data collection tool and workflow diagram, to limit subjective interpretation of case note data. However, there are limitations to this work. We evaluated patient episodes from large urban hospital sites, two of which are VTE exemplar centres and three of which are tertiary centres, which may limit external validity. Research assistants across sites varied in seniority and clinical experience; although all sites had a principal investigator and strict working definitions for outcome events, this may have introduced variation in reporting. We did not achieve our intended target of 3000 patients. However, it is important to note that the overall results within our cohort of 2008 patients are well outside of feasibility targets and sensitivity values were universally poor. We do not envisage that adding further cases would have significantly affected these values. Finally, we did not routinely collect individual patient characteristics so do not report HAT or MBE stratified by relevant variables (such as the use of thromboprophylaxis).

Strengths and weaknesses in relation to other studies, discussing important differences in results

Previous international work in this area is conflicting. A comparison of hospital episode statistics (HES) data to general practice records in England reported in 2012, initially concluded reliable identification of vascular disease (derived from ICD-10 coding data).[16] However, this analysis was restricted to pulmonary embolism from a VTE

For peer review only - http://bmjopen.bmj.com/site/about/guidelines.xhtml

perspective and sought only to correlate disease states, rather than identify new case episodes. Several authors have used primary care research datasets correlated to evidence of anticoagulation or other secondary care data to identify VTE events, with reported reliable capture. This work does not seek to discriminate between index presentation of VTE and downstream development of hospital acquired thrombosis.[17 18]

A systematic review, with searches run in July 2010 and published in 2012, summarised findings on this topic from nineteen studies. The positive predictive value (PPV) for pulmonary embolism ICD-10 codes ranged from 24% to 92%, with higher values from certain combinations of codes. PPV values for DVT codes ranged from 31% to 97%. More recently, a cross sectional North American study compared ICD-10 codes for VTE in hospitalised medical patients to a 'gold standard' manual review of clinical data in 4000 patients.[19] The authors report a sensitivity of 63% for any DVT and a sensitivity of 83% for PE, implying further discrepancy between types of VTE. Our findings align with these latter reports but offer additional validation of HAT states (in addition to VTE diagnosis) compared with routine data.

Several authors have experimented with composite data sets and diagnostic/procedural/disease coding combinations, similar to our work. One study combined ICD-10 codes for VTE with a common procedural terminology code for a VTE Diagnostic Study plus at least one of the following within 30 days of diagnosis; pharmacy script for anticoagulation, placement of an inferior vena cava filter, or death.[20] This algorithm still lacked sensitivity, reporting a value of 0.67 (0.60, 0.73) although corresponding specificity was high at 0.99 (0.98, 0.99). Alotaibi *et al* subsequently combined routinely collected ICD-10 coding data with imaging procedure codes to identify VTE events over a ten-year period, compared to case note review. Again, they report highly specific results but limited sensitivity, in line with our findings (74.83% (95% CI 67.01-81.62) and 75.24% (95% CI 65.86 to 83.14) for PE and DVT, respectively).[21] Verma et al report using natural language processing (NLP) algorithms for digital interrogation of radiology reports in a large cohort of hospitalised medical patients to identify VTE outcomes.[19] The authors conclude a high level of accuracy, reporting sensitivities of 94% / 91% and PPVs of 90% / 89% for DVT and PE, respectively. Finally, Klil-Drori et al have recently validated an algorithm for confirmation of suspected PE, combining emergency department diagnosis coding, imaging coding and dispensed prescription or hospital treatment.[22] The authors report

For peer review only - http://bmjopen.bmj.com/site/about/guidelines.xhtml

overall agreement of their algorithm with confirmed PE (adjudicated through chart review) in 92.2% cases. Again, such an algorithm would not discriminate between index diagnosis of VTE and subsequent development of HAT. Such algorithms also require external validation in a UK setting.

In 2017, Baumgartner et al highlighted further issues through interrogation of an administrative coding database, looking to determine the accuracy of ICD-10 coding for new episodes of recurrent VTE in patients with a prior history.[23] Only 31.1% of coded encounters were verified by reviewers as true recurrent VTE. More recently, Pellathy *et al* have conducted similar work within the United States, comparing accuracy of HAT diagnoses made through administrative coding to manual case note and radiology review.[24] The authors report only 40% of HAT cases identified through routine coding were confirmed by case note review and 45% of HAT confirmed through diagnostic test records lacked corresponding ICD codes.

Meaning of the study

There are multiple potential explanations for the limited performance of routine data to identify HAT. The condition is a temporal phenomenon and routine coding data can therefore mistake index presentation with VTE as HAT (false positive); patients who present with symptoms but wait >48h for CTPA confirmation of diagnosis would erroneously fit the conventional definition of HAT (VTE occurring >24h from hospital admission). International guidelines also now support outpatient diagnosis and management of VTE, so genuine cases of HAT may not require hospital admission or receive appropriate coding (false negative). These two factors are the most important contributors to poor internal validity of efficient data methods, reflected in several studies across different countries.[19 23 24] In particular, Fang et al highlight the poor performance of outpatient coding to predict VTE in a separate cohort of 4642 adult patients.[25] Finally, coding teams may fail to document subsequent HAT (false negative) following index admission with alternative pathology (such as pneumonia) and prior diagnosis of VTE can often be coded during repeat hospital attendance, mistaken for HAT (false positive). In the case of major bleeding, we found that coding of disease states with potential for bleeding (but without actual bleeding) was the biggest contributing factor to the high rate of false positive results. This issue arose due to strict definitions of major bleeding as per ISTH definition which are not mirrored by an ICD coding structure.[10]

For peer review only - http://bmjopen.bmj.com/site/about/guidelines.xhtml

Most UK hospitals conducting root cause analysis of HAT cases in line with NHS contract standards have developed pathways to mitigate these issues, through local reporting arrangements with radiology and pathology. Local leads extract all cases of DVT and PE identified by their Radiology and Ultrasound services and assess whether there was a hospital admission within 90 days prior to the VTE; if so they conduct root cause analysis by reviewing the patients notes to assess whether the VTE was potentially preventable. Such arrangements often work well, but are reliant on individuals and reporting systems subject to human error. These issues are reflected in our findings, which report a positive predictive value of 100% for HAT RCA database findings, but limited sensitivity (implying local identification of positive cases is accurate, but missed cases still occur despite a systematic approach).

Possible explanations and implications for clinicians and policymakers

More generally, these findings raise questions about the current enthusiasm for data enabled trials when outcomes are complex.[26] Such concepts are inherently attractive to researchers and patients, particularly in topic areas with low event rates. However, complex outcome measures which require temporal evaluation and qualification against prior disease states are unlikely to be reliably delivered through use of routinely collected data in isolation. For example, relevant data may contain coding errors arising from ambiguous documentation by physicians and inconsistent definitions.[27 28] Recent case studies have reported significant amounts of missing data and poor interobserver agreement between routinely collected EHR data accessible through HES and case report form evaluation.[29] Electronic records contain an abundance of free text, but often lack necessary intelligence to classify patient episodes appropriately, or allow processing and comparison of routinely collected data.[30] Increasing complexity in outcome is also likely to correspond with decreasing accuracy of routine data. A registry study of Medicare claims following mitral valve repair compared to formal adjudication, reported a positive predictive value for mortality of 97%, heart failure requiring hospitalisation of 69%, bleeding of 40% and renal failure of 19%.[31]

In addition, the time and effort needed to acquire necessary permissions for national routine coding data or to orchestrate data linkage can be substantial. A UK clinical trials unit recently reported a digital request in the context of a randomised controlled trial, highlighting a negotiation process over consent that took several years. Even after consent, the study team were in receipt of data 15 months following application.[32] Such

For peer review only - http://bmjopen.bmj.com/site/about/guidelines.xhtml

timeframes may only be realistic within the context of continually adaptive design trials.

Unanswered questions and future research

This work is restricted primarily to medical, surgical and orthopaedic patients. We did not evaluate efficient data methods for VTE or bleeding events in specific patient subgroups, such as cancer or neurosurgery. In addition, our work is UK based; other countries may be able to demonstrate more confidence in the accuracy of routinely collected data, although our review of the literature does not support this theory.

In their call to action, Sydes et al discuss supplementation of trial specific follow up as an option to realise the full potential of data-enabled research.[26] Such an approach has potential merit to attempt identification of potential HAT, given the high positive predictive value and high specificity of routine data sources. In addition, routine data sources may have a role in other research contexts, such as identification of cases for qualitative work, case control studies, targeted individual follow up or downstream survey work.

CONCLUSIONS

Our study highlights the potential limitations of using routine data methods in the context of future research on VTE risk assessment. Such methods identify both false negative and false positive VTE cases, through failure to identify ambulatory cases without formal hospital coding and overdiagnosis of prior disease. Our findings were similar with regard to bleeding events, showing poor sensitivity of ICD-10 coding data and multiple false positive events identified across four sites. These findings have implications for funders looking to support further work in this area and suggest large studies reliant on routine data collection methods in isolation are likely to be inaccurate and therefore unfeasible.

For peer review only - http://bmjopen.bmj.com/site/about/guidelines.xhtml

Contributorship statement

The authors were involved as follows: SG and DH (conception), RD, CR, SG, BH and DH (execution, analysis and drafting manuscript). SR designed and developed the iterative database. MBu and MBr conducted statistical evaluation of the dataset on behalf of the CTRU. KdW and BH attended PMG meetings and contributed to drafting of the final manuscript. All authors were involved in critical discussion, revision and final approval of the manuscript. DH acts as guarantor. The corresponding author attests that all listed authors meet authorship criteria and that no others meeting the criteria have been omitted.

Competing Interests Statement

On behalf of all authors, I declare the following competing interests:
During completion of this study, SG, DH, CR, BH, MBu and MB received funding from the National Institute of Health Research for academic work in this area, through competitive grant application and appoint to a doctoral research fellow position (CR).

Following completion of the study, CR has been employed by Pfizer limited. Pfizer did not fund nor support this study and were not involved in drafting or revising this manuscript.

Funding Information

This study was funded by the United Kingdom National Institute for Health Research (NIHR) Health Technology Assessment (HTA) Programme (project number 127454). The views expressed in this report are those of the authors and not necessarily those of the NIHR HTA Programme. Any errors are the responsibility of the authors. The funders had no role in the study design, in the collection, analysis and interpretation of data; in the writing of the manuscript; and in the decision to submit the manuscript for publication.

Data Sharing Statement

Data are not publicly available but may be obtained or interrogated via written request to the Clinical Trials Research Unit at the University of Sheffield.

For peer review only - http://bmjopen.bmj.com/site/about/guidelines.xhtml

Ethics Statement

The study received a favourable opinion from the Proportionate Review Sub-committee of the London - West London & GTAC Research Ethics Committee and approval from the HRA and Care Research Wales (HCRW) on 18th September 2019 (reference 19/LO/1303, IRAS project ID 262220).

Acknowledgements

We would like to acknowledge the support of the research nurses and assistants involved in chart review, data extraction and entry across the four hospital sites, including Reece Doonan, Efia Mainoo, Linda Debattista, Sarah Bird and Anna Wilson. We would also like to acknowledge the wider group directly conducting the VTEAM project (NIHR 127454), including project manager Helen Shulver, literature expert Abdullah Pandor, clerical assistant Heather Dakin, topic expert Xavier Griffin and clinical expert Mark Holland. We would also like to acknowledge the valuable input from the patient and public representatives, Robin Pierce-Williams, Chris Tweedy, Ben Langsdale, Deb Smith (Thrombosis UK), Shan Bennett and Enid Hirst (Sheffield Emergency Care Forum).

Study Registration:

This project is registered at https://fundingawards.nihr.ac.uk/award/NIHR127454

For peer review only

REFERENCES

1. Raskob GE, Angchaisuksiri P, Blanco AN, et al. Thrombosis: a major contributor to global disease burden. *Semin Thromb Hemost* 2014;40(7):724-35. doi: 10.1055/s-0034-1390325 [published Online First: 2014/10/11]
2. Heit JA, O'Fallon WM, Petterson TM, et al. Relative impact of risk factors for deep vein thrombosis and pulmonary embolism: a population-based study. *Arch Intern Med* 2002;162(11):1245-8. doi: 10.1001/archinte.162.11.1245 [published Online First: 2002/06/01]
3. Horner D, Goodacre S, Davis S, et al. Which is the best model to assess risk for venous thromboembolism in hospitalised patients? *BMJ* 2021;373:n1106. doi: 10.1136/bmj.n1106 [published Online First: 2021/05/29]
4. Riley RD, Ensor J, Snell KI, et al. External validation of clinical prediction models using big datasets from e-health records or IPD meta-analysis: opportunities and challenges. *BMJ* 2016;353:i3140. doi: 10.1136/bmj.i3140 [published Online First: 2016/06/24]
5. Steyerberg EW, Vergouwe Y. Towards better clinical prediction models: seven steps for development and an ABCD for validation. *Eur Heart J* 2014;35(29):1925-31. doi: 10.1093/eurheartj/ehu207 [published Online First: 2014/06/06]
6. Goodacre S, Hogg K, Holland M, et al. The cost-effectiveness of venous thromboembolism risk assessment tools for hospital inpatients https://fundingawards.nihr.ac.uk/: NIHR; 2019 [Available from: https://fundingawards.nihr.ac.uk/award/NIHR127454 accessed 19/10/2021 2021.
7. Goodacre SG, Horner D, Hogg K, et al. The cost-effectiveness of venous thromboembolism risk assessment tools for hospital inpatients fundingawards.nihr.ac.uk: NIHR; 2020 [Available from: https://fundingawards.nihr.ac.uk/award/NIHR127454 accessed 1/7 2020.
8. National Institute for Health and Care Excellence. Venous thromboembolism in over 16s: reducing the risk of hospital-acquired deep vein thrombosis or pulmonary embolism (NG89) nice.org.uk: NICE; 2018 [updated 13th August 2019; cited 2020 1/7]. Available from: https://www.nice.org.uk/guidance/ng89 accessed 22/07/2022 2020.
9. Roberts LN, Hunt B, Arya R. National Thrombosis Survey. In: GIRFT, ed. Thrombosisuk.org, 2021.
10. Schulman S, Angeras U, Bergqvist D, et al. Definition of major bleeding in clinical investigations of antihemostatic medicinal products in surgical patients. *J Thromb Haemost* 2010;8(1):202-4. doi: 10.1111/j.1538-7836.2009.03678.x [published Online First: 2009/11/03]
11. Schulman S, Kearon C, Subcommittee on Control of Anticoagulation of the S, et al. Definition of major bleeding in clinical investigations of antihemostatic medicinal products in non-surgical patients. *J Thromb Haemost* 2005;3(4):692-4. doi: 10.1111/j.1538-7836.2005.01204.x [published Online First: 2005/04/22]
12. Le Gal G, Carrier M, Castellucci LA, et al. Development and implementation of common data elements for venous thromboembolism research: on behalf of SSC Subcommittee on official Communication from the SSC of the ISTH. *J Thromb Haemost* 2021;19(1):297-303. doi: 10.1111/jth.15138 [published Online First: 2021/01/07]

For peer review only - http://bmjopen.bmj.com/site/about/guidelines.xhtml

13. Roberts LN, Porter G, Barker RD, et al. Comprehensive VTE prevention program incorporating mandatory risk assessment reduces the incidence of hospital-associated thrombosis. *Chest* 2013;144(4):1276-81. doi: 10.1378/chest.13-0267 [published Online First: 2013/05/18]
14. Newcombe RG. Two-sided confidence intervals for the single proportion: comparison of seven methods. *Stat Med* 1998;17(8):857-72. doi: 10.1002/(sici)1097-0258(19980430)17:8<857::aid-sim777>3.0.co;2-e [published Online First: 1998/05/22]
15. Hirst E, Irving A, Goodacre S. Patient and public involvement in emergency care research. *Emerg Med J* 2016;33(9):665-70. doi: 10.1136/emermed-2016-205700 [published Online First: 2016/04/06]
16. Wright FL, Green J, Canoy D, et al. Vascular disease in women: comparison of diagnoses in hospital episode statistics and general practice records in England. *BMC Med Res Methodol* 2012;12:161. doi: 10.1186/1471-2288-12-161 [published Online First: 2012/11/01]
17. Abdul Sultan A, Tata LJ, Grainge MJ, et al. The incidence of first venous thromboembolism in and around pregnancy using linked primary and secondary care data: a population based cohort study from England and comparative meta-analysis. *PLoS One* 2013;8(7):e70310. doi: 10.1371/journal.pone.0070310 [published Online First: 2013/08/08]
18. Lawrenson R, Todd JC, Leydon GM, et al. Validation of the diagnosis of venous thromboembolism in general practice database studies. *Br J Clin Pharmacol* 2000;49(6):591-6. doi: 10.1046/j.1365-2125.2000.00199.x [published Online First: 2000/06/10]
19. Verma AA, Masoom H, Pou-Prom C, et al. Developing and validating natural language processing algorithms for radiology reports compared to ICD-10 codes for identifying venous thromboembolism in hospitalized medical patients. *Thromb Res* 2021;209:51-58. doi: 10.1016/j.thromres.2021.11.020 [published Online First: 2021/12/07]
20. Sanfilippo KM, Wang TF, Gage BF, et al. Improving accuracy of International Classification of Diseases codes for venous thromboembolism in administrative data. *Thromb Res* 2015;135(4):616-20. doi: 10.1016/j.thromres.2015.01.012 [published Online First: 2015/01/24]
21. Alotaibi GS, Wu C, Senthilselvan A, et al. The validity of ICD codes coupled with imaging procedure codes for identifying acute venous thromboembolism using administrative data. *Vasc Med* 2015;20(4):364-8. doi: 10.1177/1358863X15573839 [published Online First: 2015/04/03]
22. Klil-Drori AJ, Prajapati D, Liang Z, et al. External Validation of ASPECT (Algorithm for Suspected Pulmonary Embolism Confirmation and Treatment). *Med Care* 2019;57(8):e47-e52. doi: 10.1097/MLR.0000000000001055 [published Online First: 2019/01/05]
23. Baumgartner C, Go AS, Fan D, et al. Administrative codes inaccurately identify recurrent venous thromboembolism: The CVRN VTE study. *Thromb Res* 2020;189:112-18. doi: 10.1016/j.thromres.2020.02.023 [published Online First: 2020/03/22]
24. Pellathy T, Saul M, Clermont G, et al. Accuracy of identifying hospital acquired venous thromboembolism by administrative coding: implications for big data and machine learning research. *J Clin Monit Comput* 2021 doi: 10.1007/s10877-021-00664-6 [published Online First: 2021/02/10]

For peer review only - http://bmjopen.bmj.com/site/about/guidelines.xhtml

25. Fang MC, Fan D, Sung SH, et al. Validity of Using Inpatient and Outpatient Administrative Codes to Identify Acute Venous Thromboembolism: The CVRN VTE Study. *Med Care* 2017;55(12):e137-e43. doi: 10.1097/MLR.0000000000000524 [published Online First: 2017/11/15]
26. Sydes MR, Barbachano Y, Bowman L, et al. Realising the full potential of data-enabled trials in the UK: a call for action. *BMJ Open* 2021;11(6):e043906. doi: 10.1136/bmjopen-2020-043906 [published Online First: 2021/06/18]
27. Spencer FA, Emery C, Lessard D, et al. The Worcester Venous Thromboembolism study: a population-based study of the clinical epidemiology of venous thromboembolism. *J Gen Intern Med* 2006;21(7):722-7. doi: 10.1111/j.1525-1497.2006.00458.x [published Online First: 2006/07/01]
28. Burles K, Innes G, Senior K, et al. Limitations of pulmonary embolism ICD-10 codes in emergency department administrative data: let the buyer beware. *BMC Med Res Methodol* 2017;17(1):89. doi: 10.1186/s12874-017-0361-1 [published Online First: 2017/06/10]
29. Powell GA, Bonnett LJ, Smith CT, et al. Using routinely recorded data in a UK RCT: a comparison to standard prospective data collection methods. *Trials* 2021;22(1):429. doi: 10.1186/s13063-021-05294-6 [published Online First: 2021/07/07]
30. Liaw ST, Chen HY, Maneze D, et al. Health reform: is routinely collected electronic information fit for purpose? *Emerg Med Australas* 2012;24(1):57-63. doi: 10.1111/j.1742-6723.2011.01486.x [published Online First: 2012/02/09]
31. Lowenstern A, Lippmann SJ, Brennan JM, et al. Use of Medicare Claims to Identify Adverse Clinical Outcomes After Mitral Valve Repair. *Circ Cardiovasc Interv* 2019;12(5):e007451. doi: 10.1161/CIRCINTERVENTIONS.118.007451 [published Online First: 2019/05/16]
32. Macnair A, Love SB, Murray ML, et al. Accessing routinely collected health data to improve clinical trials: recent experience of access. *Trials* 2021;22(1):340. doi: 10.1186/s13063-021-05295-5 [published Online First: 2021/05/12]

For peer review only - http://bmjopen.bmj.com/site/about/guidelines.xhtml

Supplementary material: Evaluating routine data sources to determine the incidence of hospital acquired thrombosis and major bleeding in medical and surgical inpatients.

Figure 1: Examples of electronic and paper documentation aids for RAM completion

Site 1- Structured note completed through Electronic Health Record and designed to aid VTE risk assessment at the point of hospital admission, using the Department of Health RAM (Salford Royal NHS Foundation Trust).

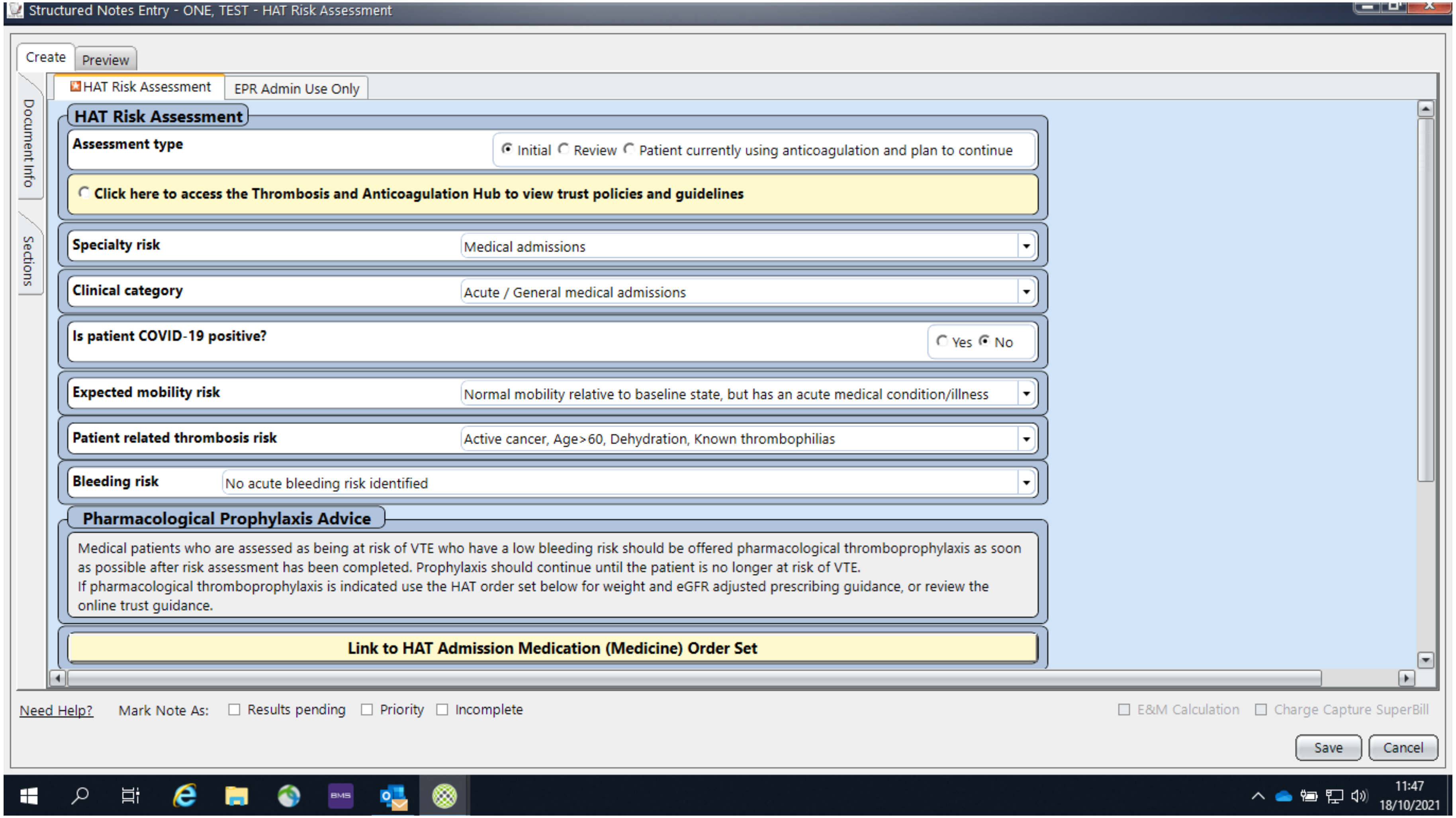

Supplementary material: Evaluating routine data sources to determine the incidence of hospital acquired thrombosis and major bleeding in medical and surgical inpatients.

Site 2 – Paper risk assessment proforma with linked prescribing (Manchester NHS Foundation Trust)

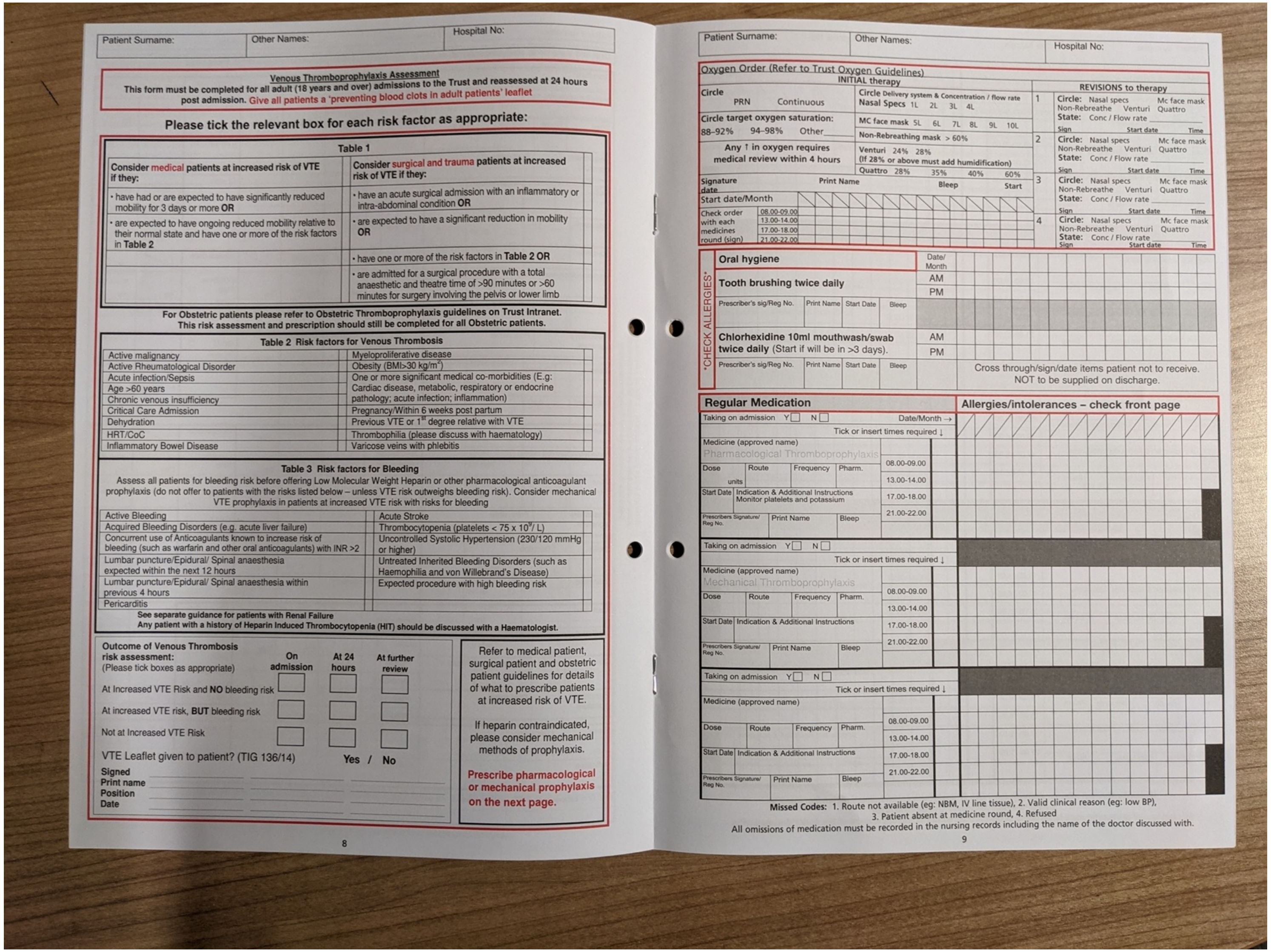

Patient Surname: | Other Names: | Hospital No:

Venous Thromboprophylaxis Assessment
This form must be completed for all adult (18 years and over) admissions to the Trust and reassessed at 24 hours post admission. Give all patients a 'preventing blood clots in adult patients' leaflet

Please tick the relevant box for each risk factor as appropriate:

Table 1	
Consider medical patients at increased risk of VTE if they:	Consider surgical and trauma patients at increased risk of VTE if they:
• have had or are expected to have significantly reduced mobility for 3 days or more **OR**	• have an acute surgical admission with an inflammatory or intra-abdominal condition **OR**
• are expected to have ongoing reduced mobility relative to their normal state and have one or more of the risk factors in **Table 2**	• are expected to have a significant reduction in mobility **OR**
	• have one or more of the risk factors in **Table 2 OR**
	• are admitted for a surgical procedure with a total anaesthetic and theatre time of >90 minutes or >60 minutes for surgery involving the pelvis or lower limb

For Obstetric patients please refer to Obstetric Thromboprophylaxis guidelines on Trust Intranet. This risk assessment and prescription should still be completed for all Obstetric patients.

Table 2 Risk factors for Venous Thrombosis	
Active malignancy	Myeloproliferative disease
Active Rheumatological Disorder	Obesity (BMI>30 kg/m²)
Acute infection/Sepsis	One or more significant medical co-morbidities (E.g: Cardiac disease, metabolic, respiratory or endocrine pathology; acute infection; inflammation)
Age >60 years	
Chronic venous insufficiency	
Critical Care Admission	Pregnancy/Within 6 weeks post partum
Dehydration	Previous VTE or 1st degree relative with VTE
HRT/CoC	Thrombophilia (please discuss with haematology)
Inflammatory Bowel Disease	Varicose veins with phlebitis

Table 3 Risk factors for Bleeding
Assess all patients for bleeding risk before offering Low Molecular Weight Heparin or other pharmacological anticoagulant prophylaxis (do not offer to patients with the risks listed below – unless VTE risk outweighs bleeding risk). Consider mechanical VTE prophylaxis in patients at increased VTE risk with risks for bleeding

Active Bleeding	Acute Stroke
Acquired Bleeding Disorders (e.g. acute liver failure)	Thrombocytopenia (platelets < 75 x 10⁹/ L)
Concurrent use of Anticoagulants known to increase risk of bleeding (such as warfarin and other oral anticoagulants) with INR >2	Uncontrolled Systolic Hypertension (230/120 mmHg or higher)
Lumbar puncture/Epidural/ Spinal anaesthesia expected within the next 12 hours	Untreated Inherited Bleeding Disorders (such as Haemophilia and von Willebrand's Disease)
Lumbar puncture/Epidural/ Spinal anaesthesia within previous 4 hours	Expected procedure with high bleeding risk
Pericarditis	

See separate guidance for patients with Renal Failure
Any patient with a history of Heparin Induced Thrombocytopenia (HIT) should be discussed with a Haematologist.

Outcome of Venous Thrombosis risk assessment: (Please tick boxes as appropriate)	On admission	At 24 hours	At further review
At Increased VTE Risk and **NO** bleeding risk			
At increased VTE risk, **BUT** bleeding risk			
Not at Increased VTE Risk			

VTE Leaflet given to patient? (TIG 136/14) Yes / No
Signed
Print name
Position
Date

Refer to medical patient, surgical patient and obstetric patient guidelines for details of what to prescribe patients at increased risk of VTE.

If heparin contraindicated, please consider mechanical methods of prophylaxis.

Prescribe pharmacological or mechanical prophylaxis on the next page.

8

Patient Surname: | Other Names: | Hospital No:

Oxygen Order (Refer to Trust Oxygen Guidelines)
INITIAL therapy | REVISIONS to therapy

Oral hygiene
Tooth brushing twice daily
Chlorhexidine 10ml mouthwash/swab twice daily (Start if will be in >3 days).
Cross through/sign/date items patient not to receive. NOT to be supplied on discharge.

Regular Medication | Allergies/intolerances – check front page
Medicine (approved name): Pharmacological Thromboprophylaxis
Medicine (approved name): Mechanical Thromboprophylaxis
Medicine (approved name)

Missed Codes: 1. Route not available (eg: NBM, IV line tissue), 2. Valid clinical reason (eg: low BP), 3. Patient absent at medicine round, 4. Refused
All omissions of medication must be recorded in the nursing records including the name of the doctor discussed with.

9

Supplementary material: Evaluating routine data sources to determine the incidence of hospital acquired thrombosis and major bleeding in medical and surgical inpatients.

Site 3 – Paper risk assessment proforma without linked prescribing (Sheffield Teaching Hospitals NHS Foundation Trust)

Sheffield Teaching Hospitals NHS
NHS Foundation Trust

RISK ASSESSMENT FOR VENOUS THROMBOEMBOLISM (VTE) FOR MEDICAL PATIENTS

Date of admission: ....

Date of assessment: ....

Name:

Date of birth:

Hospital No:

Consultant:

Patients should be risk assessed on admission to hospital, and reassessed whenever their clinical situation changes.

Tick all boxes that apply:

- ☐ Medical patients with a reduction in mobility or acute illness: → Continue full risk assessment of thrombosis and bleeding risk factors below
- ☐ Medical patients with **normal mobility and without acute illness** (including cancer patients solely admitted for therapy) → Thromboprophylaxis not usually required. *Risk assessment complete – sign box below*
- ☐ Medical patients already on therapeutic anticoagulation (e.g. heparin, warfarin, dabigatran, rivaroxaban, apixaban e.t.c) *with no intention to change* → Continue existing anticoagulation *Risk assessment complete – sign box below*

Thrombosis (VTE) Risk Factors

Patient related risk factors
- ☐ Active cancer or cancer treatment
- ☐ Age over 60 yrs
- ☐ Dehydration
- ☐ One or more significant comorbidities (e.g. heart disease; metabolic, endocrine or respiratory pathologies)
- ☐ Acute infection or inflammatory condition
- ☐ Personal history or first-degree relative with a history of VTE
- ☐ Obesity (BMI greater than 30kg/m^2)
- ☐ Use of oestrogen-containing contraceptive therapy
- ☐ Use of hormone replacement therapy (HRT)
- ☐ Varicose veins with phlebitis
- ☐ Known thrombophilia
- ☐ Pregnant or less than 6 weeks post partum – complete *antenatal (PD7262) or postnatal (PD7263) risk assessment for venous thromboembolism in pregnancy* form as appropriate

Admission related risk factors
- ☐ Significantly reduced mobility for 3 days or more (relative to normal state)
- ☐ Hip or knee replacement surgery
- ☐ Hip fracture
- ☐ Total anaesthetic plus surgical time greater than 90mins
- ☐ Surgery involving pelvis or lower limb with a total anaesthetic plus surgical time greater than 60mins
- ☐ Acute surgical admission with inflammatory or intra-abdominal condition
- ☐ Critical care admission
- ☐ Surgery with significant reduction in mobility

Bleeding Risk Factors

Patient related risk factors
- ☐ Active bleeding
- ☐ Acquired bleeding
- ☐ Uncontrolled hypertension (greater than or equal to 230mmHg systolic or 120mmHg diastolic)
- ☐ Acute stroke
- ☐ Thrombocytopaenia (platelets less than 75 x10^9/L)
- ☐ Untreated inherited bleeding disorders (e.g. haemophilia and von Willebrand's disease)

Admission related risk factors
- ☐ Neurosurgery, spinal surgery or eye surgery
- ☐ Other procedure with high bleeding risk
- ☐ Lumbar puncture/ epidural/ spinal anaesthesia expected within the next 12 hours
- ☐ Lumbar puncture/ epidural/ spinal anaesthesia within the previous 4 hours

Action taken (for full advice see "STH Guidelines for the prevention of Venous Thromboembolic Disease")
- ☐ No thrombosis risk factors present so no prophylaxis needed
- ☐ Thrombosis risk factors present but no prophylaxis prescribed: *state reason why (e.g. already on anticoagulation therapy, bleeding risk outweighs thrombosis risk)*..

Thrombosis risk factors present: ☐ dalteparin prescribed
☐ mechanical prophylaxis prescribed (if dalteparin contraindicated)

If thrombosis risk factors are present, prescribe dalteparin UNLESS bleeding risk outweighs VTE risk, or dalteparin is contraindicated; see below for doses:

**Patient's weight** to nearest kilogram	**eGFR greater than or equal to 20 mL/min/1.73m^2**	**eGFR less than 20 mL/min/1.73m^2**	**Pregnant women**
**Less than 45kg**	2500 units once daily	2500 units once daily	Refer to "*Risk assessment for VTE in pregnancy and up to 6 weeks postpartum* or "*STH Guidelines for the prevention of Venous Thromboembolic Disease*"
**45-99kg**	5000 units once daily		
**100-149kg**	7500 units once daily		
**150kg and greater**	5000 units twice a day		

[illegible]

8 of 12

Supplementary material: Evaluating routine data sources to determine the incidence of hospital acquired thrombosis and major bleeding in medical and surgical inpatients.

Site 4 – Electronic form within prescribing electronic health record designed to trigger consideration of VTE risk assessment at the point of hospital admission and prompt prescribing in accordance with local guidelines (St Thomas' NHS Foundation Trust).

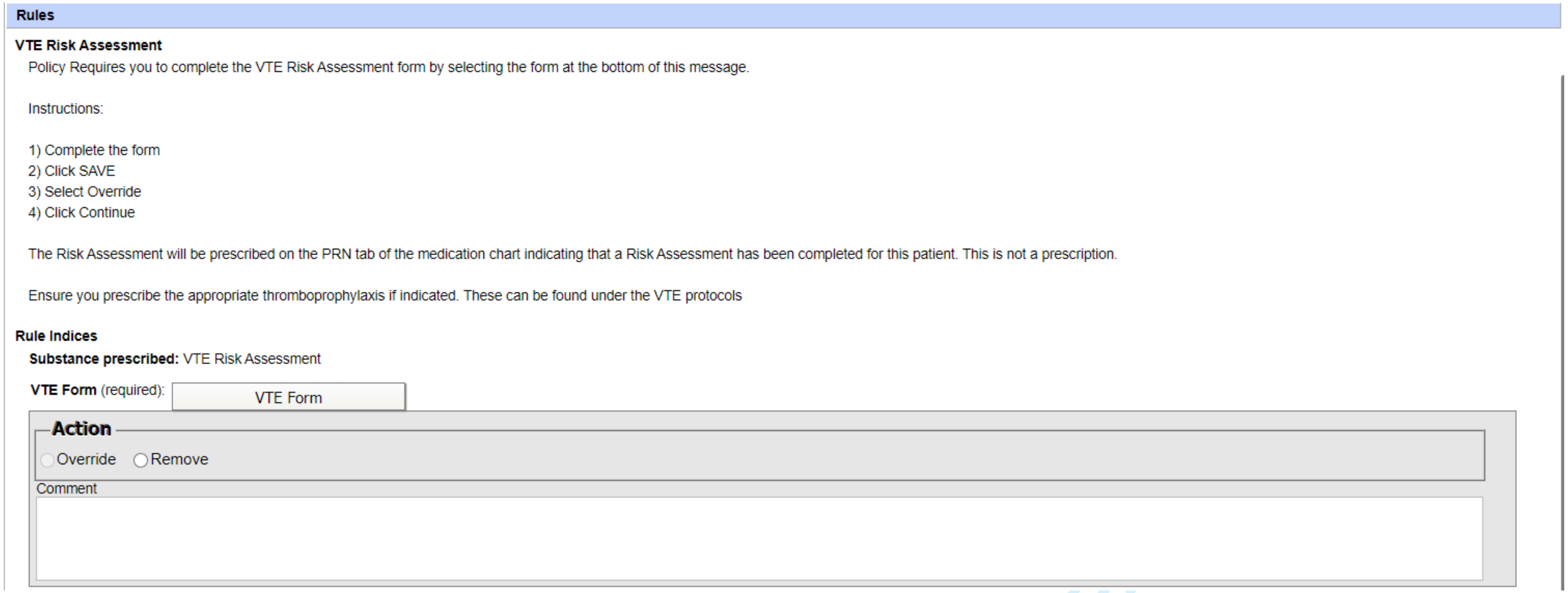

Rules

VTE Risk Assessment

Policy Requires you to complete the VTE Risk Assessment form by selecting the form at the bottom of this message.

Instructions:

1) Complete the form
2) Click SAVE
3) Select Override
4) Click Continue

The Risk Assessment will be prescribed on the PRN tab of the medication chart indicating that a Risk Assessment has been completed for this patient. This is not a prescription.

Ensure you prescribe the appropriate thromboprophylaxis if indicated. These can be found under the VTE protocols

Rule Indices

Substance prescribed: VTE Risk Assessment

VTE Form (required): VTE Form

Action

Override Remove

Comment

Supplementary data

Figure 2: Workflow diagram. CTRU – Clinical Trials Research Unit. VTEAM – Venous Thromboembolism Assessment Models (VTEAM) Study

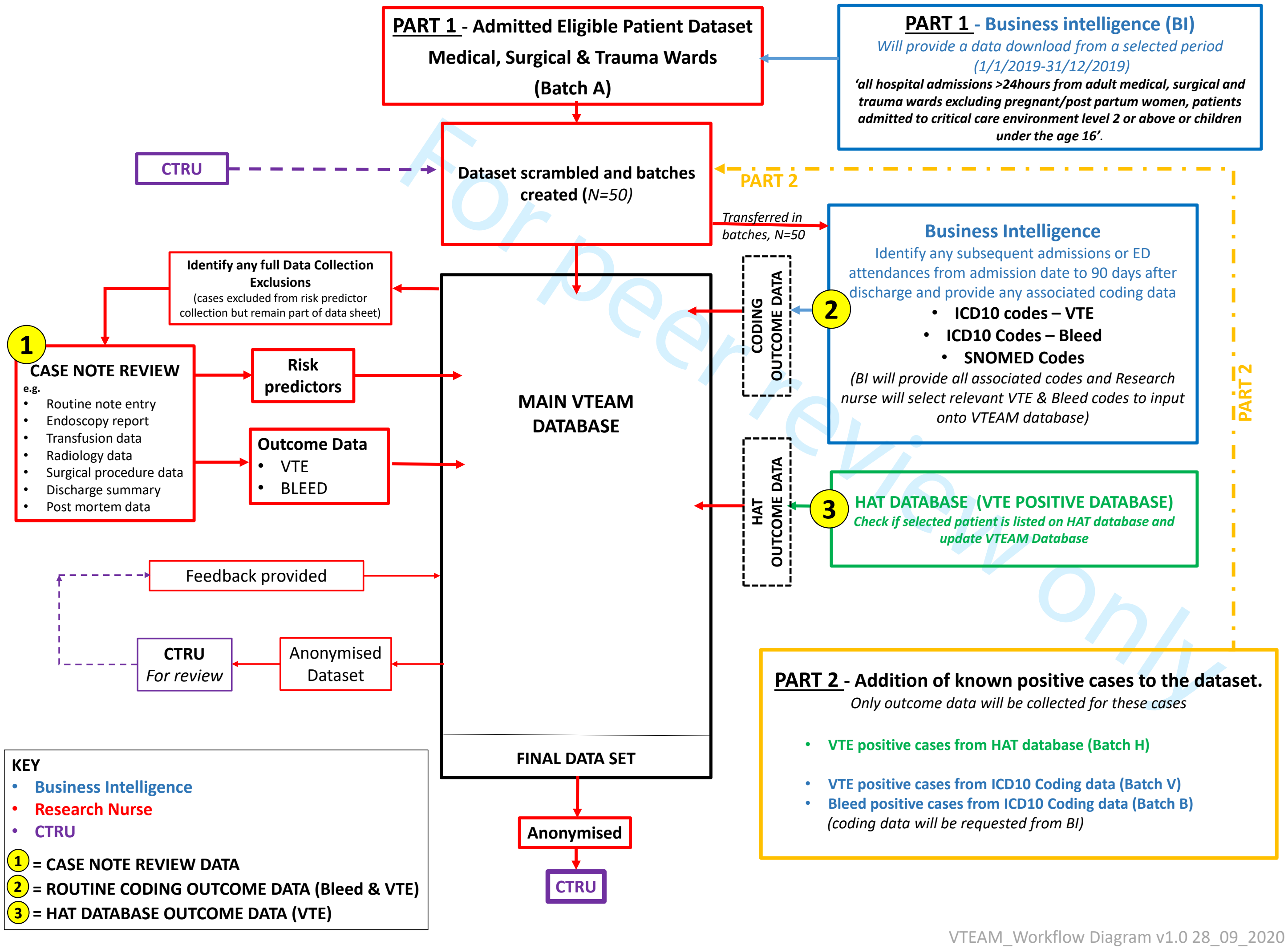

For peer review only - http://bmjopen.bmj.com/site/about/guidelines.xhtml

Supplementary data

Table 1: Relevant ICD10 codes for VTE and Bleeding agreed by chief investigators and approved by project management group

ICD10 - 4 digit	3 character description	4 character description	All diagnoses	Main diagnosis	Category	Sub-Category	Final selection
I26.0	Pulmonary embolism	Pulmonary embolism with mention of acute cor pulmonale	4031	2353	VTE		Yes
I26.9	Pulmonary embolism	Pulmonary embolism without mention of acute cor pulmonale	108637	53273	VTE		Yes
I80.1	Phlebitis and thrombophlebitis	Phlebitis and thrombophlebitis of femoral vein	10156	4294	VTE		Yes
I80.2	Phlebitis and thrombophlebitis	Phlebitis and thrombophlebitis of other deep vessels of lower extremities	61647	24297	VTE		Yes
I80.3	Phlebitis and thrombophlebitis	Phlebitis and thrombophlebitis of lower extremities, unspecified	3971	1876	VTE		Yes
I80.9	Phlebitis and thrombophlebitis	Phlebitis and thrombophlebitis of unspecified site	2906	524	VTE		Yes
I82.2	Other venous embolism and thrombosis	Embolism and thrombosis of vena cava	3891	543	VTE		Yes
I82.8	Other venous embolism and thrombosis	Embolism and thrombosis of other specified veins	10001	1870	VTE		Yes
I82.9	Other venous embolism and thrombosis	Embolism and thrombosis of unspecified vein	1124	215	VTE		Yes
I60.0	Subarachnoid haemorrhage	Subarachnoid haemorrhage from carotid siphon and bifurcation	226	212	Bleeding	intracranial bleed	Yes
I60.1	Subarachnoid haemorrhage	Subarachnoid haemorrhage from middle cerebral artery	1125	1014	Bleeding	intracranial bleed	Yes
I60.2	Subarachnoid haemorrhage	Subarachnoid haemorrhage from anterior communicating artery	1731	1599	Bleeding	intracranial bleed	Yes
I60.3	Subarachnoid haemorrhage	Subarachnoid haemorrhage from posterior communicating artery	899	838	Bleeding	intracranial bleed	Yes
I60.4	Subarachnoid haemorrhage	Subarachnoid haemorrhage from basilar artery	384	324	Bleeding	intracranial bleed	Yes
I60.5	Subarachnoid haemorrhage	Subarachnoid haemorrhage from vertebral artery	101	87	Bleeding	intracranial bleed	Yes
I60.6	Subarachnoid haemorrhage	Subarachnoid haemorrhage from other intracranial arteries	564	513	Bleeding	intracranial bleed	Yes

Supplementary data

I60.7	Subarachnoid haemorrhage	Subarachnoid haemorrhage from intracranial artery, unspecified	426	319	Bleeding	intracranial bleed	Yes
I60.8	Subarachnoid haemorrhage	Other subarachnoid haemorrhage	977	737	Bleeding	intracranial bleed	Yes
I60.9	Subarachnoid haemorrhage	Subarachnoid haemorrhage, unspecified	7642	4585	Bleeding	intracranial bleed	Yes
I61.0	Intracerebral haemorrhage	Intracerebral haemorrhage in hemisphere, subcortical	4996	4396	Bleeding	intracranial bleed	Yes
I61.1	Intracerebral haemorrhage	Intracerebral haemorrhage in hemisphere, cortical	5439	4254	Bleeding	intracranial bleed	Yes
I61.2	Intracerebral haemorrhage	Intracerebral haemorrhage in hemisphere, unspecified	1431	1157	Bleeding	intracranial bleed	Yes
I61.3	Intracerebral haemorrhage	Intracerebral haemorrhage in brain stem	1029	866	Bleeding	intracranial bleed	Yes
I61.4	Intracerebral haemorrhage	Intracerebral haemorrhage in cerebellum	1901	1508	Bleeding	intracranial bleed	Yes
I61.5	Intracerebral haemorrhage	Intracerebral haemorrhage, intraventricular	3678	1886	Bleeding	intracranial bleed	Yes
I61.6	Intracerebral haemorrhage	Intracerebral haemorrhage, multiple localized	764	561	Bleeding	intracranial bleed	Yes
I61.8	Intracerebral haemorrhage	Other intracerebral haemorrhage	3854	3103	Bleeding	intracranial bleed	Yes
I61.9	Intracerebral haemorrhage	Intracerebral haemorrhage, unspecified	11863	9028	Bleeding	intracranial bleed	Yes
I62.0	Other nontraumatic intracranial haemorrhage	Subdural haemorrhage (acute)(nontraumatic)	17161	8197	Bleeding	intracranial bleed	Yes
I62.1	Other nontraumatic intracranial haemorrhage	Nontraumatic extradural haemorrhage	318	100	Bleeding	intracranial bleed	Yes
I62.9	Other nontraumatic intracranial haemorrhage	Intracranial haemorrhage (nontraumatic), unspecified	3230	2383	Bleeding	intracranial bleed	Yes
I85.0	Oesophageal varices	Oesophageal varices with bleeding	4074	2876	Bleeding	gastrointestinal	Yes
K22.6	Other diseases of oesophagus	Gastro-oesophageal laceration-haemorrhage syndrome	7232	3237	Bleeding		Yes
K25.0	Gastric ulcer	Gastric ulcer - Acute with haemorrhage	2077	1469	Bleeding	gastrointestinal	Yes
K25.2	Gastric ulcer	Gastric ulcer - Acute with both haemorrhage and perforation	49	20	Bleeding	gastrointestinal	Yes

For peer review only

Supplementary data

K25.4	Gastric ulcer	Gastric ulcer - Chronic or unspecified with haemorrhage	4742	2951	Bleeding	gastrointestinal	Yes
K25.6	Gastric ulcer	Gastric ulcer - Chronic or unspecified with both haemorrhage and perforation	145	74	Bleeding	gastrointestinal	Yes
K26.0	Duodenal ulcer	Duodenal ulcer - Acute with haemorrhage	2955	2161	Bleeding	gastrointestinal	Yes
K26.2	Duodenal ulcer	Duodenal ulcer - Acute with both haemorrhage and perforation	126	96	Bleeding	gastrointestinal	Yes
K26.4	Duodenal ulcer	Duodenal ulcer - Chronic or unspecified with haemorrhage	7607	4972	Bleeding	gastrointestinal	Yes
K26.6	Duodenal ulcer	Duodenal ulcer - Chronic or unspecified with both haemorrhage and perforation	386	263	Bleeding	gastrointestinal	Yes
K27.0	Peptic ulcer, site unspecified	Peptic ulcer, site unspecified - Acute with haemorrhage	78	32	Bleeding	gastrointestinal	Yes
K27.2	Peptic ulcer, site unspecified	Peptic ulcer, site unspecified - Acute with both haemorrhage and perforation	4	1	Bleeding	gastrointestinal	Yes
K27.4	Peptic ulcer, site unspecified	Peptic ulcer, site unspecified - Chronic or unspecified with haemorrhage	231	116	Bleeding	gastrointestinal	Yes
K27.6	Peptic ulcer, site unspecified	Peptic ulcer, site unspecified - Chronic or unspecified with both haemorrhage and perforation	29	9	Bleeding	gastrointestinal	Yes
K28.0	Gastrojejunal ulcer	Gastrojejunal ulcer - Acute with haemorrhage	29	24	Bleeding	gastrointestinal	Yes
K28.2	Gastrojejunal ulcer	Gastrojejunal ulcer - Acute with both haemorrhage and perforation	3	3	Bleeding	gastrointestinal	Yes
K28.4	Gastrojejunal ulcer	Gastrojejunal ulcer - Chronic or unspecified with haemorrhage	149	96	Bleeding	gastrointestinal	Yes
K28.6	Gastrojejunal ulcer	Gastrojejunal ulcer - Chronic or unspecified with both haemorrhage and perforation	11	6	Bleeding	gastrointestinal	Yes
K29.0	Gastritis and duodenitis	Acute haemorrhagic gastritis	3340	1365	Bleeding	gastrointestinal	Yes
K62.5	Other diseases of anus and rectum	Haemorrhage of anus and rectum	37545	21106	Bleeding	gastrointestinal	Yes
K66.1	Other disorders of peritoneum	Haemoperitoneum	3317	642	Bleeding	gastrointestinal	Yes
K92.0	Other diseases of digestive system	Haematemesis	67589	27503	Bleeding	gastrointestinal	Yes
K92.1	Other diseases of digestive system	Melaena	67036	22979	Bleeding	gastrointestinal	Yes
K92.2	Other diseases of digestive system	Gastrointestinal haemorrhage, unspecified	192053	97428	Bleeding	gastrointestinal	Yes

Supplementary data

M25.0	Other joint disorders, not elsewhere classified	Haemarthrosis	3362	1730	Bleeding		Yes
N02.0	Recurrent and persistent haematuria	Recurrent and persistent haematuria - Minor glomerular abnormality	48	21	Bleeding		Yes
N02.1	Recurrent and persistent haematuria	Recurrent and persistent haematuria - Focal and segmental glomerular lesions	286	76	Bleeding		Yes
N02.2	Recurrent and persistent haematuria	Recurrent and persistent haematuria - Diffuse membranous glomerulonephritis	1858	517	Bleeding		Yes
N02.3	Recurrent and persistent haematuria	Recurrent and persistent haematuria - Diffuse mesangial proliferative glomerulonephritis	160	49	Bleeding		Yes
N02.4	Recurrent and persistent haematuria	Recurrent and persistent haematuria - Diffuse endocapillary proliferative glomerulonephritis	10	4	Bleeding		Yes
N02.5	Recurrent and persistent haematuria	Recurrent and persistent haematuria - Diffuse mesangiocapillary glomerulonephritis	47	12	Bleeding		Yes
N02.6	Recurrent and persistent haematuria	Recurrent and persistent haematuria - Dense deposit disease	9	1	Bleeding		Yes
N02.7	Recurrent and persistent haematuria	Recurrent and persistent haematuria - Diffuse crescentic glomerulonephritis	164	48	Bleeding		Yes
N02.8	Recurrent and persistent haematuria	Recurrent and persistent haematuria - Other	10852	1289	Bleeding		Yes
N02.9	Recurrent and persistent haematuria	Recurrent and persistent haematuria - Unspecified	2902	1331	Bleeding		Yes
N93.8	Other abnormal uterine and vaginal bleeding	Other specified abnormal uterine and vaginal bleeding	4801	2899	Bleeding	other bleed	Yes
N93.9	Other abnormal uterine and vaginal bleeding	Abnormal uterine and vaginal bleeding, unspecified	24423	11036	Bleeding	other bleed	Yes
R04.0	Haemorrhage from respiratory passages	Epistaxis	48741	24610	Bleeding	other bleed	Yes
R04.1	Haemorrhage from respiratory passages	Haemorrhage from throat	191	69	Bleeding	other bleed	Yes
R04.2	Haemorrhage from respiratory passages	Haemoptysis	32143	12743	Bleeding	other bleed	Yes
R04.8	Haemorrhage from respiratory passages	Haemorrhage from other sites in respiratory passages	1332	222	Bleeding	other bleed	Yes
R04.9	Haemorrhage from respiratory passages	Haemorrhage from respiratory passages, unspecified	83	23	Bleeding	other bleed	Yes

For peer review only

Supplementary data

R23.3	Other skin changes	Spontaneous ecchymoses	10624	2774	Bleeding		Yes
R58.X	Haemorrhage, not elsewhere classified	Haemorrhage, not elsewhere classified	2747	408	Bleeding		Yes
T81.0	Complications of procedures, not elsewhere classified	Haemorrhage and haematoma complicating a procedure, not elsewhere classified	67338	28601	Bleeding		Yes
T81.7	Complications of procedures, not elsewhere classified	Vascular complications following a procedure, not elsewhere classified	1000	201	Bleeding		Yes

For peer review only

STROBE Statement—Checklist of items that should be included in reports of ***cohort studies***

	Item No	Recommendation	Page No
**Title and abstract**	1	(*a*) Indicate the study's design with a commonly used term in the title or the abstract	1
		(*b*) Provide in the abstract an informative and balanced summary of what was done and what was found	3
**Introduction**			
Background/rationale	2	Explain the scientific background and rationale for the investigation being reported	5
Objectives	3	State specific objectives, including any prespecified hypotheses	5
**Methods**			
Study design	4	Present key elements of study design early in the paper	6-7
Setting	5	Describe the setting, locations, and relevant dates, including periods of recruitment, exposure, follow-up, and data collection	6-7
Participants	6	(*a*) Give the eligibility criteria, and the sources and methods of selection of participants. Describe methods of follow-up	6-7
		(*b*) For matched studies, give matching criteria and number of exposed and unexposed	
Variables	7	Clearly define all outcomes, exposures, predictors, potential confounders, and effect modifiers. Give diagnostic criteria, if applicable	8
Data sources/ measurement	8*	For each variable of interest, give sources of data and details of methods of assessment (measurement). Describe comparability of assessment methods if there is more than one group	7-8
Bias	9	Describe any efforts to address potential sources of bias	8
Study size	10	Explain how the study size was arrived at	8-9
Quantitative variables	11	Explain how quantitative variables were handled in the analyses. If applicable, describe which groupings were chosen and why	8
Statistical methods	12	(*a*) Describe all statistical methods, including those used to control for confounding	8
		(*b*) Describe any methods used to examine subgroups and interactions	
		(*c*) Explain how missing data were addressed	
		(*d*) If applicable, explain how loss to follow-up was addressed	
		(*e*) Describe any sensitivity analyses	
**Results**			
Participants	13*	(a) Report numbers of individuals at each stage of study—eg numbers potentially eligible, examined for eligibility, confirmed eligible, included in the study, completing follow-up, and analysed	11
		(b) Give reasons for non-participation at each stage	
		(c) Consider use of a flow diagram	
Descriptive data	14*	(a) Give characteristics of study participants (eg demographic, clinical, social) and information on exposures and potential confounders	Table 1&2, 11
		(b) Indicate number of participants with missing data for each variable of interest	
		(c) Summarise follow-up time (eg, average and total amount)	
Outcome data	15*	Report numbers of outcome events or summary measures over time	11

For peer review only - http://bmjopen.bmj.com/site/about/guidelines.xhtml

Main results	16	(*a*) Give unadjusted estimates and, if applicable, confounder-adjusted estimates and their precision (eg, 95% confidence interval). Make clear which confounders were adjusted for and why they were included<br>(*b*) Report category boundaries when continuous variables were categorized<br>(*c*) If relevant, consider translating estimates of relative risk into absolute risk for a meaningful time period	11
Other analyses	17	Report other analyses done—eg analyses of subgroups and interactions, and sensitivity analyses	12
**Discussion**			
Key results	18	Summarise key results with reference to study objectives	13
Limitations	19	Discuss limitations of the study, taking into account sources of potential bias or imprecision. Discuss both direction and magnitude of any potential bias	13-14
Interpretation	20	Give a cautious overall interpretation of results considering objectives, limitations, multiplicity of analyses, results from similar studies, and other relevant evidence	15-16
Generalisability	21	Discuss the generalisability (external validity) of the study results	16
**Other information**			
Funding	22	Give the source of funding and the role of the funders for the present study and, if applicable, for the original study on which the present article is based	2

*Give information separately for exposed and unexposed groups.

Note: An Explanation and Elaboration article discusses each checklist item and gives methodological background and published examples of transparent reporting. The STROBE checklist is best used in conjunction with this article (freely available on the Web sites of PLoS Medicine at http://www.plosmedicine.org/, Annals of Internal Medicine at http://www.annals.org/, and Epidemiology at http://www.epidem.com/). Information on the STROBE Initiative is available at http://www.strobe-statement.org.

For peer review only

BMJ Open

The accuracy of efficient data methods to determine the incidence of hospital acquired thrombosis and major bleeding in medical and surgical inpatients: a multicentre observational cohort study in four UK hospitals

Journal:	*BMJ Open*
Manuscript ID	bmjopen-2022-069244.R2
Article Type:	Original research
Date Submitted by the Author:	23-Jan-2023
Complete List of Authors:	Horner, Daniel; Northern Care Alliance NHS Foundation Trust, Emergency Department; The University of Manchester, Division of Infection, Immunity and Respiratory Medicine<br>Rex, Saleema; The University of Sheffield, School of Health and Related Research (ScHARR)<br>Reynard, Charles; The University of Manchester, Division of Cardiovascular sciences<br>Bursnall, Matthew; The University of Sheffield, School of Health and Related Research (ScHARR)<br>Bradburn, Mike; University of Sheffield, ScHARR<br>de Wit, Kerstin; McMaster University Department of Medicine, Department of Medicine; Hamilton General Hospital<br>Goodacre, Steve; University of Sheffield, Medical Care Research Unit<br>Hunt, Beverley; Guy's and St Thomas' NHS Foundation Trust, Kings Healthcare Partners & Thrombosis & Haemophilia Centre
**Primary Subject Heading**:	Haematology (incl blood transfusion)
Secondary Subject Heading:	Cardiovascular medicine, Diagnostics, Health informatics
Keywords:	Anticoagulation < HAEMATOLOGY, Thromboembolism < CARDIOLOGY, Risk management < HEALTH SERVICES ADMINISTRATION & MANAGEMENT

SCHOLARONE™
Manuscripts

I, the Submitting Author has the right to grant and does grant on behalf of all authors of the Work (as defined in the below author licence), an exclusive licence and/or a non-exclusive licence for contributions from authors who are: i) UK Crown employees; ii) where BMJ has agreed a CC-BY licence shall apply, and/or iii) in accordance with the terms applicable for US Federal Government officers or employees acting as part of their official duties; on a worldwide, perpetual, irrevocable, royalty-free basis to BMJ Publishing Group Ltd ("BMJ") its licensees and where the relevant Journal is co-owned by BMJ to the co-owners of the Journal, to publish the Work in this journal and any other BMJ products and to exploit all rights, as set out in our licence.

The Submitting Author accepts and understands that any supply made under these terms is made by BMJ to the Submitting Author unless you are acting as an employee on behalf of your employer or a postgraduate student of an affiliated institution which is paying any applicable article publishing charge ("APC") for Open Access articles. Where the Submitting Author wishes to make the Work available on an Open Access basis (and intends to pay the relevant APC), the terms of reuse of such Open Access shall be governed by a Creative Commons licence – details of these licences and which Creative Commons licence will apply to this Work are set out in our licence referred to above.

Other than as permitted in any relevant BMJ Author's Self Archiving Policies, I confirm this Work has not been accepted for publication elsewhere, is not being considered for publication elsewhere and does not duplicate material already published. I confirm all authors consent to publication of this Work and authorise the granting of this licence.

For peer review only

Title:

The accuracy of efficient data methods to determine the incidence of hospital acquired thrombosis and major bleeding in medical and surgical inpatients: a multicentre observational cohort study in four UK hospitals

Author names and positions:

Daniel Horner [1, 2]	Consultant in Emergency and Intensive Care Medicine
Saleema Rex[3]	Research Associate
Charlie Reynard [4]	Postgraduate Research Fellow
Matt Bursnall [3]	Research Associate
Mike Bradburn [3]	Senior Medical Statistician
Kerstin de Wit [5]	Associate Professor
Steve Goodacre [3]	Professor of Emergency Medicine
Beverley J. Hunt [6]	Professor of Thrombosis and Haemostasis

Affiliations:

[1] Emergency Department, Northern Care Alliance NHS Foundation Trust, Salford, UK
[2] Division of Infection, Immunity and Respiratory Medicine, University of Manchester, Oxford Road, UK
[3] Centre for Urgent and Emergency Care Research (CURE), University of Sheffield, Sheffield, UK
[4] Division of Cardiovascular Sciences, University of Manchester, Oxford Road, Manchester, UK
[5] Department of Medicine, McMaster University, Hamilton, Ontario, Canada.
[6] Kings Healthcare Partners & Thrombosis & Haemophilia Centre, Guy's & St Thomas' NHS Foundation Trust, London, UK

Corresponding author:

Daniel Horner daniel.horner@nca.nhs.uk
Mailing address: Emergency Department, Northern Care Alliance NHS Foundation Trust, Stott Lane, Salford, M6 8HD

Word Counts:

Abstract word count: 246
Total word count: 3870
References: 23

Notation of prior abstract publication/presentation:

Sections of this work were presented at the ISTH 2022 conference in London by poster. This work is also accepted for publication by the NIHR in monograph format within their journals library in April 2023. The NIHR strongly encourages wide dissemination of funded work and early submission of manuscripts, as per the embargo policy:

For peer review only

For peer review only - http://bmjopen.bmj.com/site/about/guidelines.xhtml

"In line with our embargo policy, we are prepared to delay publication of your Journals Library manuscript and, where possible, co-ordinate publication of your Journals Library manuscript with any journal articles."

More details on the NIHR Embargo policy can be found at the following address: https://www.journalslibrary.nihr.ac.uk/policies/#Embargo

Keywords:

Venous Thromboembolism

Risk Assessment

Thromboprophylaxis

Routine data

Efficient methods

For peer review only

For peer review only - http://bmjopen.bmj.com/site/about/guidelines.xhtml

Abstract

Objectives:

We evaluated the accuracy of using routine health service data to identify hospital acquired thrombosis (HAT) and major bleeding events (MBE) compared to a reference standard of case note review.

Design:

A multi-centre observational cohort study.

Setting:

Four acute hospitals in the United Kingdom.

Participants:

A consecutive unselective cohort of general medical and surgical patients requiring hospitalisation for a period of >24h during the calendar year 2021. We excluded paediatric, obstetric and critical care patients due to differential risk profiles.

Interventions:

We compared preidentified sources of routinely collected information (using hospital coding data and local contractually mandated thrombosis datasets) to data extracted from case notes using a predesigned workflow methodology.

Primary and Secondary Outcome Measures:

We defined HAT as objectively confirmed venous thromboembolism occurring during hospital stay or within 90 days of discharge and MBE as per international consensus.

Results:

We were able to source all necessary routinely collected outcome data for 87% of 2008 case episodes reviewed. The sensitivity of hospital coding data (ICD-10) for the diagnosis of HAT and MBE was 62% (95% CI 54 to 69) and 38% (95% CI 27 to 50) respectively. Sensitivity improved to 81% (95% CI 75 to 87) when using local thrombosis datasets.

Conclusions:

Using routinely collected data appeared to miss a substantial proportion of outcome events, when compared to case note review. Our study suggests that currently available routine data collection methods in the UK are inadequate to support efficient study designs in venous thromboembolism research.

Trial registration:

This project is registered at https://fundingawards.nihr.ac.uk/award/NIHR127454

For peer review only - http://bmjopen.bmj.com/site/about/guidelines.xhtml

For peer review only

Article Summary:

Strengths and Limitations of this study

- This study used predefined outcomes and international consensus definitions to evaluate the accuracy of routinely collected data for identification of hospital acquired thrombosis and major bleeding events, during hospital admission.

- All data abstractors were blinded to routine data sources, limiting bias in case ascertainment.

- Research assistants varied in clinical experience by site, which may introduce variability in outcome reporting.

- Our findings may lack generalisability to other healthcare settings, given the UK context.

For peer review only

For peer review only - http://bmjopen.bmj.com/site/about/guidelines.xhtml

BACKGROUND

Venous Thromboembolism (VTE) remains a major global health burden, with significant attributable morbidity and mortality.[1] At least half of all VTE occurs during hospitalisation, or up to 90 days following discharge; such cases are described as Hospital Acquired Thrombosis (HAT).[2] Many of these events are potentially preventable through patient education and provision of thromboprophylaxis to those at risk.

Research into thromboprophylaxis often requires large sample sizes to identify small but important differences in clinically relevant events, such as HAT and/or major bleeding. Study protocols will often necessitate examination of case notes to identify outcome events, which can be time consuming and expensive. This is particularly relevant for external validation of new clinical decision rules or risk assessment models which aim to guide prescribing of thromboprophylaxis for hospital inpatients.[3-5]

Using routine health service data to identify outcome events could markedly improve the efficiency of research and facilitate studies with large sample sizes at acceptable cost. However, this approach requires confirmatory evidence that routine data sources accurately identify outcome events.

Several mechanisms already exist for routine identification of outcomes, including hospital coding, local VTE datasets, and pathology reporting (with thrombosis committee oversight). If such efficient methods could accurately ascertain relevant outcomes, large scale studies would be theoretically deliverable.

We sought to evaluate the accuracy of using routine data to identify HAT and major bleeding events (MBE) compared to case note examination.

For peer review only

METHODS

We conducted a multi-centre observational cohort study within the context of a wider project examining the overall clinical and cost effectiveness of VTE risk assessment models.[6] The aim of this study was to estimate the accuracy and completeness of available coding data and local registry data to determine clinically relevant VTE and bleeding outcomes against case note review by trained research assistants.

We approached four NHS (National Health Service) sites to participate in this study; the Northern Care Alliance NHS Foundation Trust, Manchester University Hospitals NHS Foundation Trust, the Northern General Hospital in Sheffield and Guy's & St Thomas' NHS Foundation Trust.

Study Population

We identified a consecutive, unselected cohort of general medical and surgical patients requiring hospital admission at each site during the calendar year 1st January to 31st December 2019. We chose 2019 because of concern that patients admitted during the subsequent COVID-19 pandemic might represent an atypical cohort. We collated data on all risk assessments that clinical staff performed prospectively at the point of hospital admission, then scrambled episodes into randomly assorted batches of 50 (referred to as 'A' batches) to ensure diversity in specialty presentation and mitigate seasonal bias. We collated cases (or records) in batches of 50 to facilitate iterative and incremental case ascertainment; initial hospital downloads were often in excess of 50,000 case episodes. In order to keep workflow manageable and organized, we worked through batches of 50 records at a time and reported routinely to a steering committee, who provided guidance on study delivery. We excluded paediatric patients (age <16), anyone requiring critical care admission (defined as level 2 care or above) and pregnant/post-partum patients due to differential VTE and bleeding risks, as outlined in the wider study protocol.[7]

Study design

For each patient episode, we extracted baseline demographics and prospectively collected data on VTE risk assessment (where available) from the electronic health record, with support from business intelligence teams. Risk assessments were captured

For peer review only - http://bmjopen.bmj.com/site/about/guidelines.xhtml

differently at each site, including the use of a paper proforma, dichotomous output on electronic prescribing (low/high risk), or through a detailed structured note within the electronic healthcare record. Example images/screenshots for each site can be found in the supplementary material. All four sites used the Department of Health tool to facilitate VTE risk assessment; this Risk Assessment Model (RAM) has been developed by expert consensus and is recommended in national UK guidance.[8] Recent survey data suggests the tool is used by >80% of NHS sites, despite limited available data on external validation.[9] This tool confers a high rate of prescribing in comparison with other RAMs, as highlighted in a recent practice review.[3]

Research assistants at each site undertook retrospective case note review for each patient episode through shared primary and secondary care electronic health records (EHR). We utilised EHR to access primary care data on hospital attendance, diagnoses and investigation within the relevant time periods. Primary care EHR systems varied by trust. We used secondary care EHR to identify hospital reattendance, investigations, diagnostic imaging and confirmed diagnoses (via discharge summary or note entry). Secondary care EHR systems varied by trust, but access to radiology investigations was universal within the patient archiving and communication system (PACS). We extracted descriptive data on relevant clinical outcomes such as the subsequent diagnosis of VTE/HAT, major bleeding and clinically relevant non-major bleeding events as per internationally agreed definitions.[10 11] We defined VTE as any pulmonary embolism or deep vein thrombosis identified in routine care by the treating clinical team, in accordance with ISTH common data elements.[12] Superficial venous thrombosis was specifically excluded from this definition. We defined HAT in accordance with the definition proposed by NHS England (any new episode of VTE occurring during hospitalisation or within 90 days of discharge, following an inpatient stay of ≥2 days or a surgical procedure under general/regional anaesthesia).[13] Data extractors were trained in identification of these outcomes and followed a detailed workflow diagram (supplementary material). Data abstractors were blinded to batch allocation, final ICD-10 coding, local database entries and the final analysis plan.

Following case note review, we collected data elements from multiple data sources for each patient episode to evaluate their combined accuracy against case note review

For peer review only - http://bmjopen.bmj.com/site/about/guidelines.xhtml

outcomes. Data sources for interrogation were identified a-priori and included the following; ICD-10 diagnostic codes judged relevant to thrombosis or bleeding by the project management group (a-priori, shown in the supplementary material); Emergency Care Data Set/SNOMED codes relevant to thrombosis or bleeding; and contractual local Hospital Acquired Thrombosis (HAT) databases. HAT database entries are co-ordinated by local thrombosis committees in accordance with NHS contract standards and include a contemporary register of all patients diagnosed with acute VTE at the hospital site, informed by radiology or identified by pathology at post mortem.[13] All cases are subsequently categorised by the local thrombosis committee as either de-novo VTE or HAT based on case review, local expert opinion and data on any preceding hospital admission (up to 90 days) or VTE diagnosis >24h following hospital admission. This database is maintained contemporaneously and provides an ongoing opportunity for hospitals to identify preventable HAT and conduct root cause analysis (RCA) for each episode, to promote learning and best practice. All data sources were interrogated for the duration of hospital stay and up to 90 days post discharge, for each patient episode. Data sources were obtained through routine local business intelligence requests or direct approach to local coding teams. HAT database entries were obtained where feasible through local site thrombosis committee chairs.

Given the potential for negligible VTE/MBE in the wider study population (leading to limited information on the accuracy of efficient data methods), we augmented the overall sample with positive thrombosis and bleeding cases. We obtained positive cases through ICD-10 coding identification for VTE events (V batches), bleeding events (B batches) and positive VTE cases from local HAT database entries (H batches), identified as above and sourced from local thrombosis committee leads. Positive cases were batched and reviewed in accordance with the general study protocol. Data extractors were blinded to batch allocation.

Outcomes

The following criteria were proposed to determine whether routine data identify outcome events with sufficient accuracy to support efficient methods:

1. Proportion of outcome events identified by routine data sources that are confirmed by record review (target 100%)

For peer review only - http://bmjopen.bmj.com/site/about/guidelines.xhtml

2. Proportion of cases with no outcome event identified by routine data sources that have an event identified on record review (target 0%)
3. Proportion of inpatients with data collected (target 90%)

Statistical analysis

The accuracy of routinely recorded HAT and bleeding events was compared against direct case note review data for the cohort. Case note review determination of events was assumed to be the gold standard. Data are presented in contingency tables with sensitivity, specificity, positive and negative predictive values along with confidence intervals calculated using the Wilson score method.[14]

The primary analysis included patients identified from all sources (A, V, B or H batches) with bleeding and HAT assumed to have not occurred unless coded as such in the relevant data, or detected following case note review. In addition, two pre-planned sensitivity analyses were undertaken:

a. Inclusion was limited to cases identified in routine case review (i.e. "A batch patients" only), with exclusion of all augmented sample cases.
b. Inclusion limited to participants for whom bleeding or HAT was definitively recorded

We took a conservative approach and interpreted missing or unknown endpoints as 'no event' with the exception of the second sensitivity analysis.

We originally planned to identify 3000 inpatients across 4 hospitals during a 12-month study period within the 2-year project plan, dependent on appointment of research assistants and time required for outcome ascertainment. This sample size was designed to allow key parameters to be estimated with a high degree of precision across the whole cohort (standard error <1%). All sites failed to meet their sample target of 750 for reasons mainly related to the SARs COV-2 pandemic, including redeployment of research staff to clinical care, delayed local approvals secondary to prioritised pandemic research and a longer than anticipated time for individual outcome ascertainment per case review

Ethical Aspects

For peer review only - http://bmjopen.bmj.com/site/about/guidelines.xhtml

The study received a favourable opinion from the Proportionate Review Sub-committee of the London - West London & GTAC Research Ethics Committee and approval from the HRA and Care Research Wales (HCRW) on 18th September 2019 (reference 19/LO/1303, IRAS project ID 262220).

Participating sites identified members of the clinical care team (research nurses or assistants predominately) to access patient records and extract clinical data using a predesigned and protected Microsoft Excel© database with embedded macro function, hosted at site. All data subsequently underwent local de-identification following completion and were exported to an independent team of statisticians at the Clinical Trials Research Unit (CTRU) in Sheffield, for collation and analysis.

All aspects of the data collection process, export, analysis and oversight were regularly reviewed by the internal Project Management Group (PMG) including CTRU representation, and an external Trial Steering Committee (TSC), throughout the duration of the project.

We conducted this study in accordance with international EQUATOR guidelines. A STROBE reporting checklist was used throughout to inform design, conduct and analysis of this observational cohort study and is included as supplementary information.

Patient and Public Involvement

Representatives of two Patient and Public Involvement groups (PPI), thrombosis UK and Sheffield Emergency Care Forum joined the research team and were involved in developing the initial proposal and undertaking the wider study.

The Sheffield Emergency Care Forum (SECF) is a patient and public representative group with an interest in emergency care research. The forum has provided PPI for many emergency care research projects over then last ten years.[15] Thrombosis UK is a charity that aims to identify, inform and partner the NHS, healthcare providers and individuals to work to improve prevention of VTE and the management and care of VTE events (see https://www.thrombosisuk.org/).

For peer review only - http://bmjopen.bmj.com/site/about/guidelines.xhtml

The PPI members were involved in determining the study design and ensuring that the proposal addressed the needs of patients and the NHS, while respecting the needs of potential participants. Their input regarding the importance of providing thromboprophylaxis for potential participants of any prospective cohort study and the need for such a study to yield reliable findings was instrumental in determining our approach to answering the research question. The PPI members also provided input at project management meetings and, where required, in day-to-day running of the project. The members used meetings and surveys of their wider PPI groups to enhance PPI in the project.

For peer review only

RESULTS

We identified 2115 patients with an original hospital admission occurring in the calendar year 2019. Of these, 107 patient episodes were ineligible due to being pregnant or post-partum women (n=49); admitted to a critical care environment of level 2 or above (n=38); children aged under 16 (n=13) or for unrecorded reasons (n=7) leaving 2008 episodes for analysis. All episodes were suitable for data extraction and comparison to routine data sources.

Patient episodes showed an even balance of medical and surgical cases, but with a focus on emergency (73.7%) rather than elective (25.8%) admissions. A broad range of subspecialty interests were represented within the cohort. Median length of stay was three days (IQR 3 to 8) and mean length of stay 7.75 days (SD 16.5). Specialty groups with frequencies and cumulative percentages are shown in Table 1. The vast majority of patient episodes (1809, 90.1%) were taken from 'A' batches. The total sample was augmented by 45 patients (2.2%) with potential bleeding events and 154 (7.7%) patients with potential VTE events. All sites contributed evenly to the sample with one exception; reduced numbers at this site reflect a delay to institutional approval during the pandemic, arising from a high burden of other clinical research studies and high staff turnover. Site and batch numbers are shown in Table 2

Main Findings

Contingency tables for the accuracy of routine data sources compared to case note review for both HAT and major bleeding events are shown in Table 3. Sensitivity was 62% (95% CI 54 to 69) for the use of ICD-10/SNOMED coding data to detect HAT events and 81% (95% CI 75 to 87) for local HAT database entries. Sensitivity by individual site ranged from 45% (95% CI, 28 to 63) to 72% (95% CI, 61 to 82) using ICD-10/SNOMED coding and 68% (95% CI, 51 to 84) to 94% (95% CI 87 to 100) using local HAT database entries.

The sensitivity of ICD10/SNOMED coding to detect major bleeding events identified by case note review was 38% (95% CI 27 to 50). Sensitivity by individual site ranged from 22% (95% CI 0 to 49) to 56% (95% CI 37 to 75).

For peer review only - http://bmjopen.bmj.com/site/about/guidelines.xhtml

Table 1: Clinical category and admission type, with frequency and cumulative percentage

	Frequency	Percentage	Cumulative
**Admission type**			
Missing	1	0.05	0.05
Elective	518	25.80	25.85
Emergency	1,480	73.71	99.55
Unknown	9	0.45	100
Total	2,008	100	
**Specialty Group**			
Missing	9	0.45	0.45
Medical	902	44.92	45.37
Surgical	951	47.36	92.73
Tertiary specialty	146	7.27	100
Total	2,008	100	
**Clinical Category**			
Missing	9	0.45	0.45
Acute medicine	340	16.93	17.38
Ageing and complex medicine	133	6.62	24.0
Cardiology	41	2.04	26.04
Cardiothoracic Surgery	87	4.33	30.38
Dermatology	2	0.1	30.48
Emergency Medicine	87	4.33	34.81
Gastroenterology	61	3.04	37.85
General Surgery	285	14.19	52.04
Medical: Other	169	8.42	60.46
Neurology	10	0.5	60.96
Neurorehabilitation	2	0.1	61.06
Neurosurgery	39	1.94	63.0
Gynaecology	57	2.84	65.84
Renal Medicine	26	1.29	67.13
Respiratory	63	3.14	70.27
Rheumatology	2	0.1	70.37
Trauma and Orthopaedics	158	7.87	78.24
Upper GI Surgery	13	0.65	78.89
Urology	107	5.33	84.22
Surgery: Other	170	8.47	92.69
Tertiary specialty: Other	147	7.32	100
Total	2,008	100	

For peer review only - http://bmjopen.bmj.com/site/about/guidelines.xhtml

Table 2: Number of cases submitted by site and batch type

A – Patient admissions requiring routine risk assessment
B – Potential cases of bleeding (selected from relevant ICD-10 codes)
H – Cases of Hospital Acquired Thrombosis (HAT) identified through local thrombosis committee infrastructure
V - Potential cases of venous thromboembolic disease (selected from relevant ICD-10 codes)

	Batch				Total
	A	B	H	V	
GSTT	504	0	21	0	525
Manchester	241	0	0	0	241
Salford	570	45	44	46	705
Sheffield	494	0	43	0	537
Total	1809	45	108	46	2008

For peer review only - http://bmjopen.bmj.com/site/about/guidelines.xhtml

Table 3: Contingency tables for main outcomes

HAT = Hospital Acquired Thrombosis
*Manchester site excluded from this analysis as unable to access local HAT database.

		**HAT from case note review**		
		Yes	No	
**HAT from ICD10/SNOMED codes**	Yes	95	39	71% (63%, 79%) True positive rate and 95% CI
	No	59	1815	3% (2%, 4%) False negative rate and 95% CI
		62% (54%, 69%) Sensitivity and 95% CI	98% (97%, 99%) Specificity and 95% CI	(N=2008)
		Yes	No	
**HAT from HAT RCA database**	Yes	122	0	100% (100%, 100%) True positive rate and 95% CI
	No	29	1616	2% (1%, 2%) False negative rate and 95% CI
		81% (75%, 87%) Sensitivity and 95% CI	100% (100%, 100%) Specificity and 95% CI	(N=1767)*
		**Major bleed from case note review**		
		Yes	No	
**Major Bleed from ICD10/SNOMED codes**	Yes	25	98	20% (13%, 27%) True positive rate and 95% CI
	No	40	1845	2% (1%, 3%) False negative rate and 95% CI
		38% (27%, 50%) Sensitivity and 95% CI	95% (94%, 96%) Specificity and 95% CI	(N=2008)

For peer review only

Pre-planned Sensitivity Analysis

A sensitivity analysis was conducted using only patient episodes obtained through ‘A’ batches, to remove augmentation of the sample and mitigate bias. The sensitivity of efficient data methods to detect key outcomes identified at case note review remained poor. These results are summarised in Table 4.

We found the HAT event rate on case note review to be 29/1809 (1.6%, 95% CI 1.0 to 2.2) and the major bleeding event rate to be 45/1809 (2.5%, 95% CI 1.8 to 3.2) within this large cohort of hospitalised patients receiving risk assessment and thromboprophylaxis in the context of routine care.

The proportion of outcome HAT events identified by routine data sources that were confirmed by record review (target 100%) was 71% (95% CI 63 to 79) for ICD-10/SNOMED coding and 100% (95% CI 97 to 100) for local HAT database entries. The proportion of cases with no HAT outcome event identified by routine data sources that had an event identified on record review (target 0%) was 3% (95% CI 2 to 4) for ICD-10/SNOMED coding and 2% (95% CI 1 to 2) for local HAT database entries. The proportion of major bleeding events identified by routine data sources that were confirmed by record review (target 100%) was 20% (95% CI 13 to 27) for ICD-10/SNOMED coding. The proportion of cases with no major bleeding outcome event identified by routine data sources that have an event identified on record review (target 0%) was 2% (95% CI 1 to 3) for ICD-10/SNOMED coding. We were able to collect outcome data for 1745/2008 (87%) inpatients (target 90%). This was <100% due to difficulty accessing the local HAT database at a single site. Excluding this issue, the other three sites all managed to collect relevant outcome data for at least 98% of patients.

For peer review only - http://bmjopen.bmj.com/site/about/guidelines.xhtml

Table 4: Sensitivity analysis using only A batch cases. N=1809 following removal of H/B/V batch patients. *Manchester unable to access HAT database

		**HAT from case note review**		
		Yes	No	
**HAT from ICD10/SNOMED codes**	Yes	18	18	50% (34%, 66%) True positive rate and 95% CI
	No	11	1762	1% (0%, 1%) False negative rate and 95% CI
		62% (44%, 80%) Sensitivity and 95% CI	99% (99%, 99%) Specificity and 95% CI	(N=1809)
		Yes	No	
**HAT from HAT RCA database**	Yes	7	0	100% (100%, 100%) True positive rate and 95% CI
	No	19	1542	1% (1%, 2%) False negative rate and 95% CI
		27% (10%, 44%) Sensitivity and 95% CI	100% (100%, 100%) Specificity and 95% CI	(N=1568)*
		**Major bleed from case note review**		
		Yes	No	
**Bleed from ICD10/SNOMED codes**	Yes	14	68	17% (9%, 25%) True positive rate and 95% CI
	No	31	1696	2% (1%, 2%) False negative rate and 95% CI
		31% (18%, 45%) Sensitivity and 95% CI	96% (95%, 97%) Specificity and 95% CI	(N=1809)

For peer review only

DISCUSSION

Statement of principal findings

Our findings suggest that using currently available routine data for identification of HAT and MBE during hospital admission or within 90 days of discharge is not sufficiently sensitive to support a large data-enabled study. We failed to demonstrate feasibility for a number of predefined metrics and conclude that use of routine data to identify outcomes would be highly likely to miss important events, and may erroneously identify false positive events.

Strengths and weaknesses of the study

We engaged a combination of digitally mature and paper-based UK NHS sites in this study, used strict consensus definitions for VTE/bleeding events and evaluated only predefined efficient data sources. We also used topic experts and research staff to iteratively develop our data collection tool and workflow diagram, to limit subjective interpretation of case note data. However, there are limitations to this work. We evaluated patient episodes from large urban hospital sites, two of which are VTE exemplar centres and three of which are tertiary centres, which may limit external validity. Research assistants across sites varied in seniority and clinical experience; although all sites had a principal investigator and strict working definitions for outcome events, this may have introduced variation in reporting. We did not achieve our intended target of 3000 patients. However, it is important to note that the overall results within our cohort of 2008 patients are well outside of feasibility targets and sensitivity values were universally poor. We do not envisage that adding further cases would have significantly affected these values. Finally, we did not routinely collect individual patient characteristics so do not report HAT or MBE stratified by relevant variables (such as the use of thromboprophylaxis).

Strengths and weaknesses in relation to other studies, discussing important differences in results

Previous international work in this area is conflicting. A comparison of hospital episode statistics (HES) data to general practice records in England reported in 2012, initially concluded reliable identification of vascular disease (derived from ICD-10 coding data).[16] However, this analysis was restricted to pulmonary embolism from a VTE

For peer review only - http://bmjopen.bmj.com/site/about/guidelines.xhtml

perspective and sought only to correlate disease states, rather than identify new case episodes. Several authors have used primary care research datasets correlated to evidence of anticoagulation or other secondary care data to identify VTE events, with reported reliable capture. This work does not seek to discriminate between index presentation of VTE and downstream development of hospital acquired thrombosis.[17 18]

A systematic review, with searches run in July 2010 and published in 2012, summarised findings on this topic from nineteen studies. The positive predictive value (PPV) for pulmonary embolism ICD-10 codes ranged from 24% to 92%, with higher values from certain combinations of codes. PPV values for DVT codes ranged from 31% to 97%. More recently, a cross sectional North American study compared ICD-10 codes for VTE in hospitalised medical patients to a 'gold standard' manual review of clinical data in 4000 patients.[19] The authors report a sensitivity of 63% for any DVT and a sensitivity of 83% for PE, implying further discrepancy between types of VTE. Our findings align with these latter reports but offer additional validation of HAT states (in addition to VTE diagnosis) compared with routine data.

Several authors have experimented with composite data sets and diagnostic/procedural/disease coding combinations, similar to our work. One study combined ICD-10 codes for VTE with a common procedural terminology code for a VTE Diagnostic Study plus at least one of the following within 30 days of diagnosis; pharmacy script for anticoagulation, placement of an inferior vena cava filter, or death.[20] This algorithm still lacked sensitivity, reporting a value of 0.67 (0.60, 0.73) although corresponding specificity was high at 0.99 (0.98, 0.99). Alotaibi *et al* subsequently combined routinely collected ICD-10 coding data with imaging procedure codes to identify VTE events over a ten-year period, compared to case note review. Again, they report highly specific results but limited sensitivity, in line with our findings (74.83% (95% CI 67.01-81.62) and 75.24% (95% CI 65.86 to 83.14) for PE and DVT, respectively).[21] Verma et al report using natural language processing (NLP) algorithms for digital interrogation of radiology reports in a large cohort of hospitalised medical patients to identify VTE outcomes.[19] The authors conclude a high level of accuracy, reporting sensitivities of 94% / 91% and PPVs of 90% / 89% for DVT and PE, respectively. Finally, Klil-Drori et al have recently validated an algorithm for confirmation of suspected PE, combining emergency department diagnosis coding, imaging coding and dispensed prescription or hospital treatment.[22] The authors report

For peer review only - http://bmjopen.bmj.com/site/about/guidelines.xhtml

overall agreement of their algorithm with confirmed PE (adjudicated through chart review) in 92.2% cases. Again, such an algorithm would not discriminate between index diagnosis of VTE and subsequent development of HAT. Such algorithms also require external validation in a UK setting.

In 2017, Baumgartner et al highlighted further issues through interrogation of an administrative coding database, looking to determine the accuracy of ICD-10 coding for new episodes of recurrent VTE in patients with a prior history.[23] Only 31.1% of coded encounters were verified by reviewers as true recurrent VTE. More recently, Pellathy *et al* have conducted similar work within the United States, comparing accuracy of HAT diagnoses made through administrative coding to manual case note and radiology review.[24] The authors report only 40% of HAT cases identified through routine coding were confirmed by case note review and 45% of HAT confirmed through diagnostic test records lacked corresponding ICD codes.

Meaning of the study

There are multiple potential explanations for the limited performance of routine data to identify HAT. The condition is a temporal phenomenon and routine coding data can therefore mistake index presentation with VTE as HAT (false positive); patients who present with symptoms but wait >48h for radiological confirmation of diagnosis would erroneously fit the conventional definition of HAT (VTE occurring >24h from hospital admission). International guidelines also now support outpatient diagnosis and management of VTE, so genuine cases of HAT may not require hospital admission or receive appropriate coding (false negative). These two factors are the most important contributors to poor internal validity of efficient data methods, reflected in several studies across different countries.[19 23 24] In particular, Fang et al highlight the poor performance of outpatient coding to predict VTE in a separate cohort of 4642 adult patients.[25] Finally, coding teams may fail to document subsequent HAT (false negative) following index admission with alternative pathology (such as pneumonia) and prior diagnosis of VTE can often be coded during repeat hospital attendance, mistaken for HAT (false positive). In the case of major bleeding, we found that coding of disease states with potential for bleeding (but without actual bleeding) was the biggest contributing factor to the high rate of false positive results. This issue arose due to strict definitions of major bleeding as per ISTH definition which are not mirrored by an ICD coding structure.[10]

For peer review only - http://bmjopen.bmj.com/site/about/guidelines.xhtml

Most UK hospitals conducting root cause analysis of HAT cases in line with NHS contract standards have developed pathways to mitigate these issues, through local reporting arrangements with radiology and pathology. Local leads extract all cases of DVT and PE identified by their Radiology and Ultrasound services and assess whether there was a hospital admission within 90 days prior to the VTE; if so they conduct root cause analysis by reviewing the patients notes to assess whether the VTE was potentially preventable. Such arrangements often work well, but are reliant on individuals and reporting systems subject to human error. These issues are reflected in our findings, which report a positive predictive value of 100% for HAT RCA database findings, but limited sensitivity (implying local identification of positive cases is accurate, but missed cases still occur despite a systematic approach).

Possible explanations and implications for clinicians and policymakers

More generally, these findings raise questions about the current enthusiasm for data enabled trials when outcomes are complex.[26] Such concepts are inherently attractive to researchers and patients, particularly in topic areas with low event rates. However, complex outcome measures which require temporal evaluation and qualification against prior disease states are unlikely to be reliably delivered through use of routinely collected data in isolation. For example, relevant data may contain coding errors arising from ambiguous documentation by physicians and inconsistent definitions.[27 28] Recent case studies have reported significant amounts of missing data and poor interobserver agreement between routinely collected EHR data accessible through HES and case report form evaluation.[29] Electronic records contain an abundance of free text, but often lack necessary intelligence to classify patient episodes appropriately, or allow processing and comparison of routinely collected data.[30] Increasing complexity in outcome is also likely to correspond with decreasing accuracy of routine data. A registry study of Medicare claims following mitral valve repair compared to formal adjudication, reported a positive predictive value for mortality of 97%, heart failure requiring hospitalisation of 69%, bleeding of 40% and renal failure of 19%.[31]

In addition, the time and effort needed to acquire necessary permissions for national routine coding data or to orchestrate data linkage can be substantial. A UK clinical trials unit recently reported a digital request in the context of a randomised controlled trial, highlighting a negotiation process over consent that took several years. Even after consent, the study team were in receipt of data 15 months following application.[32] Such

For peer review only - http://bmjopen.bmj.com/site/about/guidelines.xhtml

timeframes may only be realistic within the context of continually adaptive design trials.

Unanswered questions and future research

This work is restricted primarily to medical, surgical and orthopaedic patients. We did not evaluate efficient data methods for VTE or bleeding events in specific patient subgroups, such as cancer or neurosurgery. In addition, our work is UK based; other countries may be able to demonstrate more confidence in the accuracy of routinely collected data, although our review of the literature does not support this theory.

In their call to action, Sydes et al discuss supplementation of trial specific follow up as an option to realise the full potential of data-enabled research.[26] Such an approach has potential merit to attempt identification of potential HAT, given the high positive predictive value and high specificity of routine data sources. In addition, routine data sources may have a role in other research contexts, such as identification of cases for qualitative work, case control studies, targeted individual follow up or downstream survey work.

CONCLUSIONS

Our study highlights the potential limitations of using routine data methods in the context of future research on VTE risk assessment. Such methods identify both false negative and false positive VTE cases, through failure to identify ambulatory cases without formal hospital coding and overdiagnosis of prior disease. Our findings were similar with regard to bleeding events, showing poor sensitivity of ICD-10 coding data and multiple false positive events identified across four sites. These findings have implications for funders looking to support further work in this area and suggest large studies reliant on routine data collection methods in isolation are likely to be inaccurate and therefore unfeasible.

For peer review only - http://bmjopen.bmj.com/site/about/guidelines.xhtml

Contributorship statement

The authors were involved as follows: SG and DH (conception), RD, CR, SG, BH and DH (execution, analysis and drafting manuscript). SR designed and developed the iterative database. MBu and MBr conducted statistical evaluation of the dataset on behalf of the CTRU. KdW and BH attended PMG meetings and contributed to drafting of the final manuscript. All authors were involved in critical discussion, revision and final approval of the manuscript. DH acts as guarantor. The corresponding author attests that all listed authors meet authorship criteria and that no others meeting the criteria have been omitted.

Competing Interests Statement

On behalf of all authors, I declare the following competing interests:

During the completion of this study, SG, DH, CR, BH, MBu and MB received funding from the National Institute of Health Research (NIHR) for academic work in this area, through competitive grant application and CR was appointed to an NIHR doctoral research fellow position.

Following the completion of this study, CR has been subsequently employed by Pfizer limited. Pfizer did not fund nor support this study and was not involved in drafting or revising this manuscript.

Funding Information

This study was funded by the United Kingdom National Institute for Health Research (NIHR) Health Technology Assessment (HTA) Programme (project number 127454). The views expressed in this report are those of the authors and not necessarily those of the NIHR HTA Programme. Any errors are the responsibility of the authors. The funders had no role in the study design, in the collection, analysis and interpretation of data; in the writing of the manuscript; and in the decision to submit the manuscript for publication.

Data Sharing Statement

Data are not publicly available but may be obtained or interrogated via written request to the Clinical Trials Research Unit at the University of Sheffield.

For peer review only - http://bmjopen.bmj.com/site/about/guidelines.xhtml

For peer review only

Ethics Statement

The study received a favourable opinion from the Proportionate Review Sub-committee of the London - West London & GTAC Research Ethics Committee and approval from the HRA and Care Research Wales (HCRW) on 18th September 2019 (reference 19/LO/1303, IRAS project ID 262220).

Acknowledgements

We would like to acknowledge the support of the research nurses and assistants involved in chart review, data extraction and entry across the four hospital sites, including Reece Doonan, Efia Mainoo, Linda Debattista, Sarah Bird and Anna Wilson. We would also like to acknowledge the wider group directly conducting the VTEAM project (NIHR 127454), including project manager Helen Shulver, literature expert Abdullah Pandor, clerical assistant Heather Dakin, topic expert Xavier Griffin and clinical expert Mark Holland. We would also like to acknowledge the valuable input from the patient and public representatives, Robin Pierce-Williams, Chris Tweedy, Ben Langsdale, Deb Smith (Thrombosis UK), Shan Bennett and Enid Hirst (Sheffield Emergency Care Forum).

Study Registration:

This project is registered at https://fundingawards.nihr.ac.uk/award/NIHR127454

For peer review only - http://bmjopen.bmj.com/site/about/guidelines.xhtml

REFERENCES

1. Raskob GE, Angchaisuksiri P, Blanco AN, et al. Thrombosis: a major contributor to global disease burden. *Semin Thromb Hemost* 2014;40(7):724-35. doi: 10.1055/s-0034-1390325 [published Online First: 2014/10/11]
2. Heit JA, O'Fallon WM, Petterson TM, et al. Relative impact of risk factors for deep vein thrombosis and pulmonary embolism: a population-based study. *Arch Intern Med* 2002;162(11):1245-8. doi: 10.1001/archinte.162.11.1245 [published Online First: 2002/06/01]
3. Horner D, Goodacre S, Davis S, et al. Which is the best model to assess risk for venous thromboembolism in hospitalised patients? *BMJ* 2021;373:n1106. doi: 10.1136/bmj.n1106 [published Online First: 2021/05/29]
4. Riley RD, Ensor J, Snell KI, et al. External validation of clinical prediction models using big datasets from e-health records or IPD meta-analysis: opportunities and challenges. *BMJ* 2016;353:i3140. doi: 10.1136/bmj.i3140 [published Online First: 2016/06/24]
5. Steyerberg EW, Vergouwe Y. Towards better clinical prediction models: seven steps for development and an ABCD for validation. *Eur Heart J* 2014;35(29):1925-31. doi: 10.1093/eurheartj/ehu207 [published Online First: 2014/06/06]
6. Goodacre S, Hogg K, Holland M, et al. The cost-effectiveness of venous thromboembolism risk assessment tools for hospital inpatients https://fundingawards.nihr.ac.uk/: NIHR; 2019 [Available from: https://fundingawards.nihr.ac.uk/award/NIHR127454 accessed 19/10/2021 2021.
7. Goodacre SG, Horner D, Hogg K, et al. The cost-effectiveness of venous thromboembolism risk assessment tools for hospital inpatients fundingawards.nihr.ac.uk: NIHR; 2020 [Available from: https://fundingawards.nihr.ac.uk/award/NIHR127454 accessed 1/7 2020.
8. National Institute for Health and Care Excellence. Venous thromboembolism in over 16s: reducing the risk of hospital-acquired deep vein thrombosis or pulmonary embolism (NG89) nice.org.uk: NICE; 2018 [updated 13th August 2019; cited 2020 1/7]. Available from: https://www.nice.org.uk/guidance/ng89 accessed 22/07/2022 2020.
9. Roberts LN, Hunt B, Arya R. National Thrombosis Survey. In: GIRFT, ed. Thrombosisuk.org, 2021.
10. Schulman S, Angeras U, Bergqvist D, et al. Definition of major bleeding in clinical investigations of antihemostatic medicinal products in surgical patients. *J Thromb Haemost* 2010;8(1):202-4. doi: 10.1111/j.1538-7836.2009.03678.x [published Online First: 2009/11/03]
11. Schulman S, Kearon C, Subcommittee on Control of Anticoagulation of the S, et al. Definition of major bleeding in clinical investigations of antihemostatic medicinal products in non-surgical patients. *J Thromb Haemost* 2005;3(4):692-4. doi: 10.1111/j.1538-7836.2005.01204.x [published Online First: 2005/04/22]
12. Le Gal G, Carrier M, Castellucci LA, et al. Development and implementation of common data elements for venous thromboembolism research: on behalf of SSC Subcommittee on official Communication from the SSC of the ISTH. *J Thromb Haemost* 2021;19(1):297-303. doi: 10.1111/jth.15138 [published Online First: 2021/01/07]

For peer review only - http://bmjopen.bmj.com/site/about/guidelines.xhtml

13. Roberts LN, Porter G, Barker RD, et al. Comprehensive VTE prevention program incorporating mandatory risk assessment reduces the incidence of hospital-associated thrombosis. *Chest* 2013;144(4):1276-81. doi: 10.1378/chest.13-0267 [published Online First: 2013/05/18]
14. Newcombe RG. Two-sided confidence intervals for the single proportion: comparison of seven methods. *Stat Med* 1998;17(8):857-72. doi: 10.1002/(sici)1097-0258(19980430)17:8<857::aid-sim777>3.0.co;2-e [published Online First: 1998/05/22]
15. Hirst E, Irving A, Goodacre S. Patient and public involvement in emergency care research. *Emerg Med J* 2016;33(9):665-70. doi: 10.1136/emermed-2016-205700 [published Online First: 2016/04/06]
16. Wright FL, Green J, Canoy D, et al. Vascular disease in women: comparison of diagnoses in hospital episode statistics and general practice records in England. *BMC Med Res Methodol* 2012;12:161. doi: 10.1186/1471-2288-12-161 [published Online First: 2012/11/01]
17. Abdul Sultan A, Tata LJ, Grainge MJ, et al. The incidence of first venous thromboembolism in and around pregnancy using linked primary and secondary care data: a population based cohort study from England and comparative meta-analysis. *PLoS One* 2013;8(7):e70310. doi: 10.1371/journal.pone.0070310 [published Online First: 2013/08/08]
18. Lawrenson R, Todd JC, Leydon GM, et al. Validation of the diagnosis of venous thromboembolism in general practice database studies. *Br J Clin Pharmacol* 2000;49(6):591-6. doi: 10.1046/j.1365-2125.2000.00199.x [published Online First: 2000/06/10]
19. Verma AA, Masoom H, Pou-Prom C, et al. Developing and validating natural language processing algorithms for radiology reports compared to ICD-10 codes for identifying venous thromboembolism in hospitalized medical patients. *Thromb Res* 2021;209:51-58. doi: 10.1016/j.thromres.2021.11.020 [published Online First: 2021/12/07]
20. Sanfilippo KM, Wang TF, Gage BF, et al. Improving accuracy of International Classification of Diseases codes for venous thromboembolism in administrative data. *Thromb Res* 2015;135(4):616-20. doi: 10.1016/j.thromres.2015.01.012 [published Online First: 2015/01/24]
21. Alotaibi GS, Wu C, Senthilselvan A, et al. The validity of ICD codes coupled with imaging procedure codes for identifying acute venous thromboembolism using administrative data. *Vasc Med* 2015;20(4):364-8. doi: 10.1177/1358863X15573839 [published Online First: 2015/04/03]
22. Klil-Drori AJ, Prajapati D, Liang Z, et al. External Validation of ASPECT (Algorithm for Suspected Pulmonary Embolism Confirmation and Treatment). *Med Care* 2019;57(8):e47-e52. doi: 10.1097/MLR.0000000000001055 [published Online First: 2019/01/05]
23. Baumgartner C, Go AS, Fan D, et al. Administrative codes inaccurately identify recurrent venous thromboembolism: The CVRN VTE study. *Thromb Res* 2020;189:112-18. doi: 10.1016/j.thromres.2020.02.023 [published Online First: 2020/03/22]
24. Pellathy T, Saul M, Clermont G, et al. Accuracy of identifying hospital acquired venous thromboembolism by administrative coding: implications for big data and machine learning research. *J Clin Monit Comput* 2021 doi: 10.1007/s10877-021-00664-6 [published Online First: 2021/02/10]

For peer review only - http://bmjopen.bmj.com/site/about/guidelines.xhtml

25. Fang MC, Fan D, Sung SH, et al. Validity of Using Inpatient and Outpatient Administrative Codes to Identify Acute Venous Thromboembolism: The CVRN VTE Study. *Med Care* 2017;55(12):e137-e43. doi: 10.1097/MLR.0000000000000524 [published Online First: 2017/11/15]
26. Sydes MR, Barbachano Y, Bowman L, et al. Realising the full potential of data-enabled trials in the UK: a call for action. *BMJ Open* 2021;11(6):e043906. doi: 10.1136/bmjopen-2020-043906 [published Online First: 2021/06/18]
27. Spencer FA, Emery C, Lessard D, et al. The Worcester Venous Thromboembolism study: a population-based study of the clinical epidemiology of venous thromboembolism. *J Gen Intern Med* 2006;21(7):722-7. doi: 10.1111/j.1525-1497.2006.00458.x [published Online First: 2006/07/01]
28. Burles K, Innes G, Senior K, et al. Limitations of pulmonary embolism ICD-10 codes in emergency department administrative data: let the buyer beware. *BMC Med Res Methodol* 2017;17(1):89. doi: 10.1186/s12874-017-0361-1 [published Online First: 2017/06/10]
29. Powell GA, Bonnett LJ, Smith CT, et al. Using routinely recorded data in a UK RCT: a comparison to standard prospective data collection methods. *Trials* 2021;22(1):429. doi: 10.1186/s13063-021-05294-6 [published Online First: 2021/07/07]
30. Liaw ST, Chen HY, Maneze D, et al. Health reform: is routinely collected electronic information fit for purpose? *Emerg Med Australas* 2012;24(1):57-63. doi: 10.1111/j.1742-6723.2011.01486.x [published Online First: 2012/02/09]
31. Lowenstern A, Lippmann SJ, Brennan JM, et al. Use of Medicare Claims to Identify Adverse Clinical Outcomes After Mitral Valve Repair. *Circ Cardiovasc Interv* 2019;12(5):e007451. doi: 10.1161/CIRCINTERVENTIONS.118.007451 [published Online First: 2019/05/16]
32. Macnair A, Love SB, Murray ML, et al. Accessing routinely collected health data to improve clinical trials: recent experience of access. *Trials* 2021;22(1):340. doi: 10.1186/s13063-021-05295-5 [published Online First: 2021/05/12]

For peer review only

For peer review only - http://bmjopen.bmj.com/site/about/guidelines.xhtml

Supplementary material: Evaluating routine data sources to determine the incidence of hospital acquired thrombosis and major bleeding in medical and surgical inpatients.

Figure 1: Examples of electronic and paper documentation aids for RAM completion

Site 1- Structured note completed through Electronic Health Record and designed to aid VTE risk assessment at the point of hospital admission, using the Department of Health RAM (Salford Royal NHS Foundation Trust).

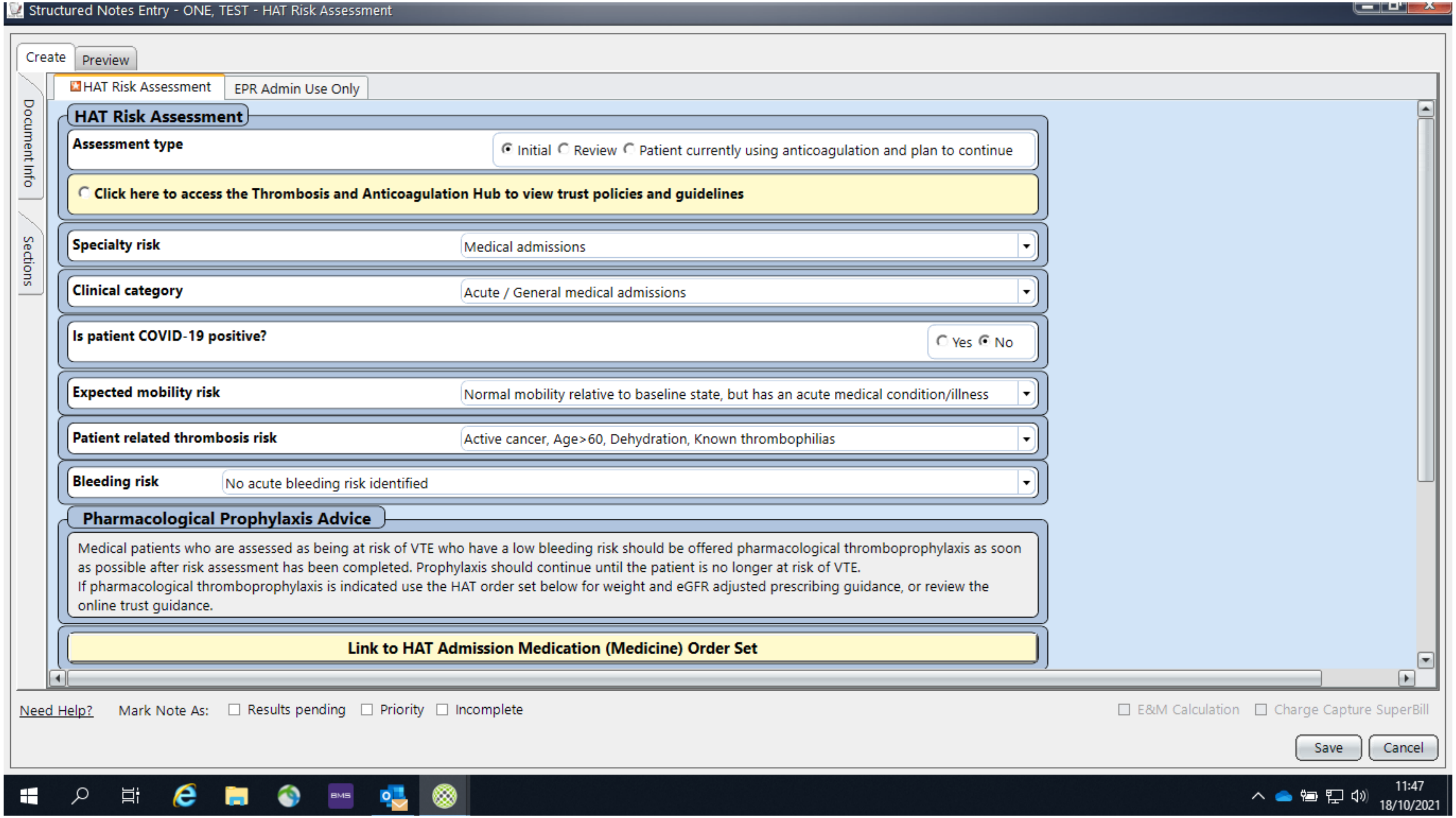

Supplementary material: Evaluating routine data sources to determine the incidence of hospital acquired thrombosis and major bleeding in medical and surgical inpatients.

Site 2 – Paper risk assessment proforma with linked prescribing (Manchester NHS Foundation Trust)

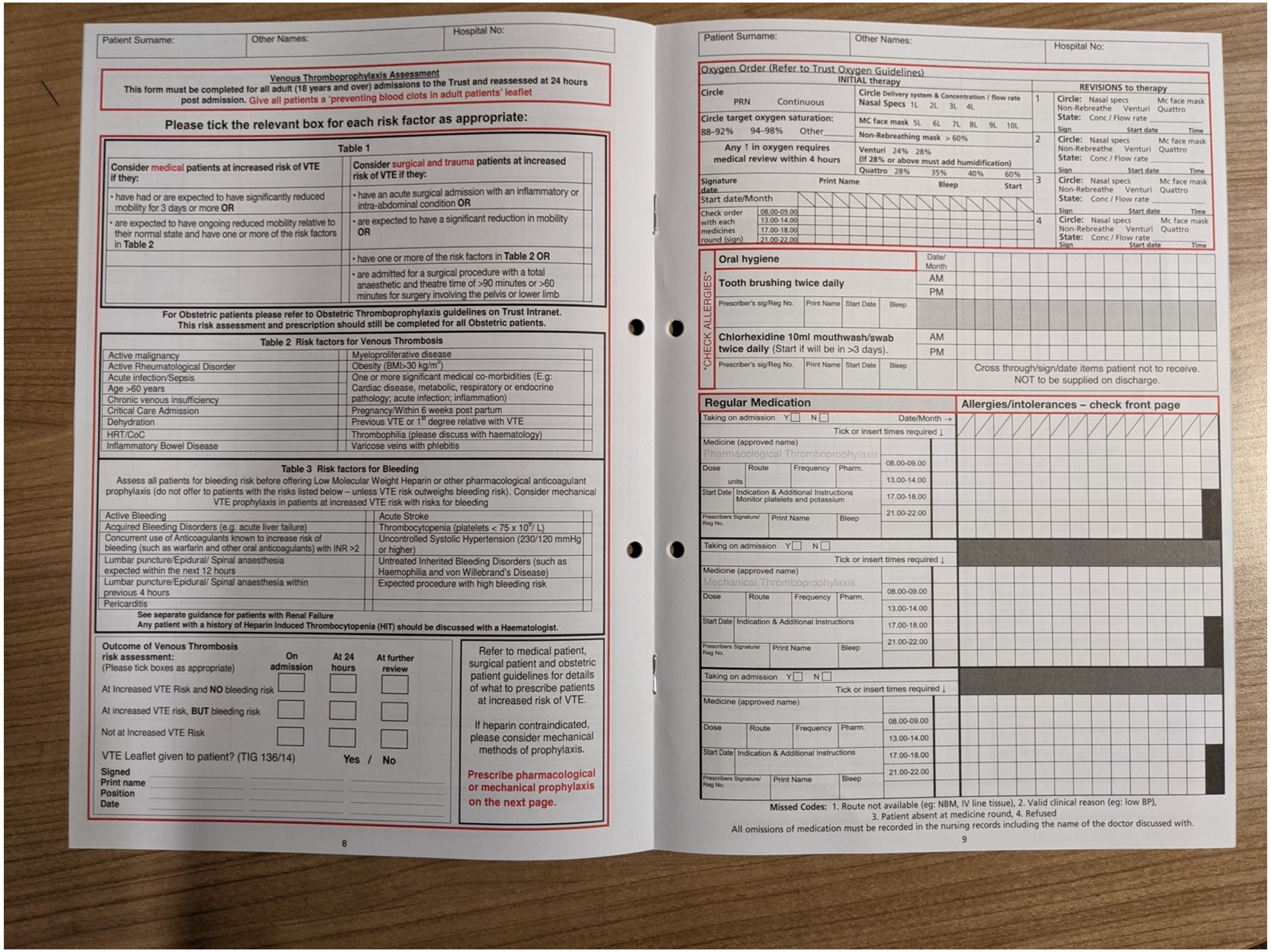

Patient Surname: | Other Names: | Hospital No:

Venous Thromboprophylaxis Assessment
This form must be completed for all adult (18 years and over) admissions to the Trust and reassessed at 24 hours post admission. Give all patients a 'preventing blood clots in adult patients' leaflet

Please tick the relevant box for each risk factor as appropriate:

Table 1	
Consider medical patients at increased risk of VTE if they:	Consider surgical and trauma patients at increased risk of VTE if they:
• have had or are expected to have significantly reduced mobility for 3 days or more **OR**	• have an acute surgical admission with an inflammatory or intra-abdominal condition **OR**
• are expected to have ongoing reduced mobility relative to their normal state and have one or more of the risk factors in **Table 2**	• are expected to have a significant reduction in mobility **OR**
	• have one or more of the risk factors in **Table 2 OR**
	• are admitted for a surgical procedure with a total anaesthetic and theatre time of >90 minutes or >60 minutes for surgery involving the pelvis or lower limb

For Obstetric patients please refer to Obstetric Thromboprophylaxis guidelines on Trust Intranet. This risk assessment and prescription should still be completed for all Obstetric patients.

Table 2 Risk factors for Venous Thrombosis	
Active malignancy	Myeloproliferative disease
Active Rheumatological Disorder	Obesity (BMI>30 kg/m²)
Acute infection/Sepsis	One or more significant medical co-morbidities (E.g: Cardiac disease, metabolic, respiratory or endocrine pathology; acute infection; inflammation)
Age >60 years	
Chronic venous insufficiency	
Critical Care Admission	Pregnancy/Within 6 weeks post partum
Dehydration	Previous VTE or 1st degree relative with VTE
HRT/CoC	Thrombophilia (please discuss with haematology)
Inflammatory Bowel Disease	Varicose veins with phlebitis

Table 3 Risk factors for Bleeding
Assess all patients for bleeding risk before offering Low Molecular Weight Heparin or other pharmacological anticoagulant prophylaxis (do not offer to patients with the risks listed below – unless VTE risk outweighs bleeding risk). Consider mechanical VTE prophylaxis in patients at increased VTE risk with risks for bleeding

Active Bleeding	Acute Stroke
Acquired Bleeding Disorders (e.g. acute liver failure)	Thrombocytopenia (platelets < 75 x 10⁹/ L)
Concurrent use of Anticoagulants known to increase risk of bleeding (such as warfarin and other oral anticoagulants) with INR >2	Uncontrolled Systolic Hypertension (230/120 mmHg or higher)
Lumbar puncture/Epidural/ Spinal anaesthesia expected within the next 12 hours	Untreated Inherited Bleeding Disorders (such as Haemophilia and von Willebrand's Disease)
Lumbar puncture/Epidural/ Spinal anaesthesia within previous 4 hours	Expected procedure with high bleeding risk
Pericarditis	

See separate guidance for patients with Renal Failure
Any patient with a history of Heparin Induced Thrombocytopenia (HIT) should be discussed with a Haematologist.

Outcome of Venous Thrombosis risk assessment: (Please tick boxes as appropriate)	On admission	At 24 hours	At further review
At Increased VTE Risk and **NO** bleeding risk			
At increased VTE risk, **BUT** bleeding risk			
Not at Increased VTE Risk			

VTE Leaflet given to patient? (TIG 136/14) Yes / No
Signed
Print name
Position
Date

Refer to medical patient, surgical patient and obstetric patient guidelines for details of what to prescribe patients at increased risk of VTE.

If heparin contraindicated, please consider mechanical methods of prophylaxis.

Prescribe pharmacological or mechanical prophylaxis on the next page.

8

Patient Surname: | Other Names: | Hospital No:

Oxygen Order (Refer to Trust Oxygen Guidelines)
INITIAL therapy | REVISIONS to therapy
Circle PRN Continuous | Circle Delivery system & Concentration / flow rate
Nasal Specs 1L 2L 3L 4L
Circle target oxygen saturation: 88–92% 94–98% Other
MC face mask 5L 6L 7L 8L 9L 10L
Non-Rebreathing mask > 60%
Any ↑ in oxygen requires medical review within 4 hours
Venturi 24% 28% (If 28% or above must add humidification)
Quattro 28% 35% 40% 60%
Signature date | Print Name | Bleep | Start
Start date/Month
Check order with each medicines round (sign) 08.00-09.00 13.00-14.00 17.00-18.00 21.00-22.00
1–4: Circle: Nasal specs Mc face mask Non-Rebreathe Venturi Quattro State: Conc / Flow rate Sign Start date Time

'CHECK ALLERGIES'
Oral hygiene | Date/Month
Tooth brushing twice daily AM PM
Prescriber's sig/Reg No. | Print Name | Start Date | Bleep
Chlorhexidine 10ml mouthwash/swab twice daily (Start if will be in >3 days). AM PM
Prescriber's sig/Reg No. | Print Name | Start Date | Bleep
Cross through/sign/date items patient not to receive. NOT to be supplied on discharge.

Regular Medication | Allergies/intolerances – check front page
Taking on admission Y N | Date/Month →
Tick or insert times required ↓
Medicine (approved name) Pharmacological Thromboprophylaxis
Dose units | Route | Frequency | Pharm. | 08.00-09.00 13.00-14.00 17.00-18.00 21.00-22.00
Start Date | Indication & Additional Instructions Monitor platelets and potassium
Prescribers Signature/ Reg No. | Print Name | Bleep

Taking on admission Y N
Tick or insert times required ↓
Medicine (approved name) Mechanical Thromboprophylaxis
Dose | Route | Frequency | Pharm. | 08.00-09.00 13.00-14.00 17.00-18.00 21.00-22.00
Start Date | Indication & Additional Instructions
Prescribers Signature/ Reg No. | Print Name | Bleep

Taking on admission Y N
Tick or insert times required ↓
Medicine (approved name)
Dose | Route | Frequency | Pharm. | 08.00-09.00 13.00-14.00 17.00-18.00 21.00-22.00
Start Date | Indication & Additional Instructions
Prescribers Signature/ Reg No. | Print Name | Bleep

Missed Codes: 1. Route not available (eg: NBM, IV line tissue), 2. Valid clinical reason (eg: low BP), 3. Patient absent at medicine round, 4. Refused
All omissions of medication must be recorded in the nursing records including the name of the doctor discussed with.

9

Supplementary material: Evaluating routine data sources to determine the incidence of hospital acquired thrombosis and major bleeding in medical and surgical inpatients.

Site 3 – Paper risk assessment proforma without linked prescribing (Sheffield Teaching Hospitals NHS Foundation Trust)

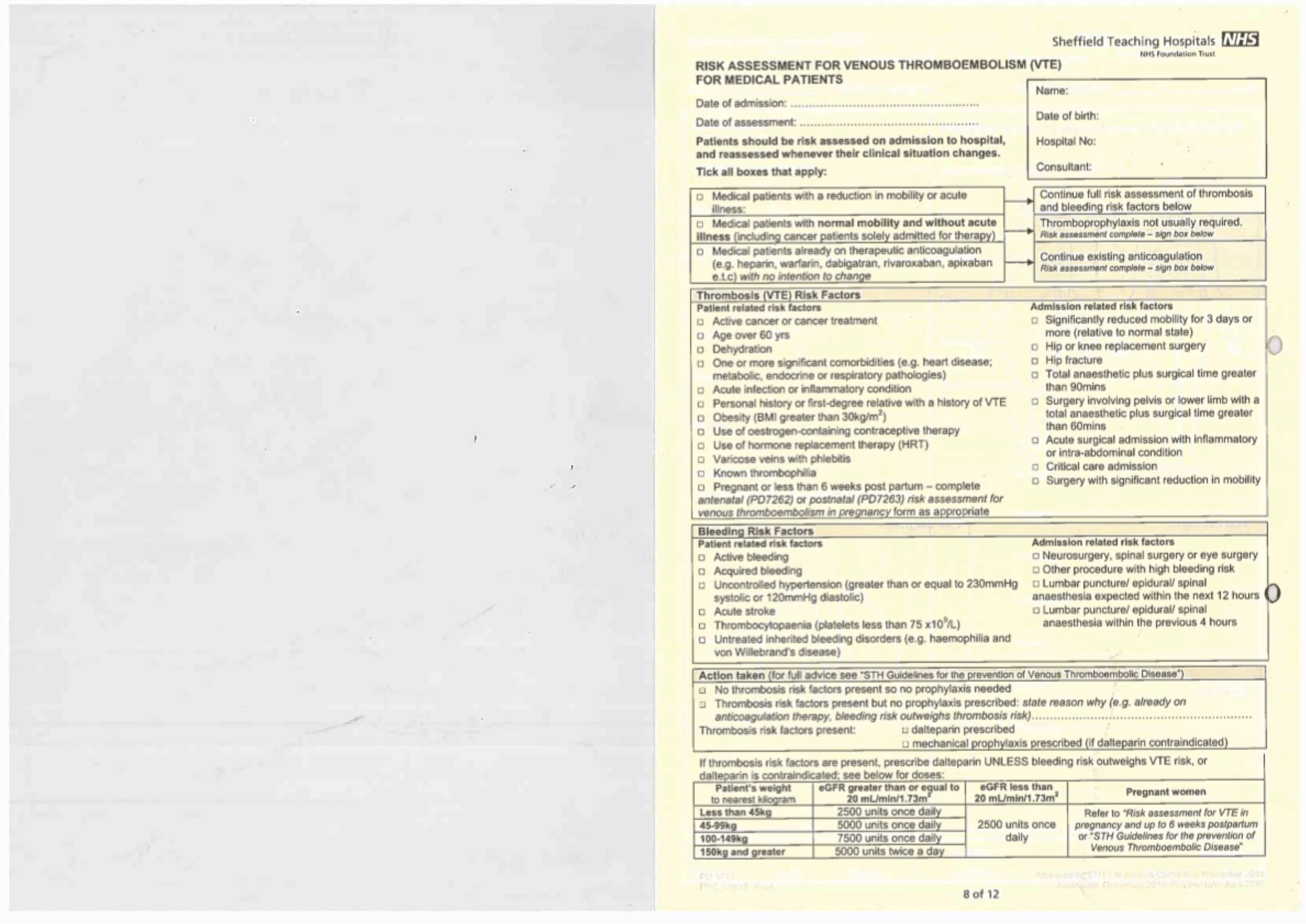

Sheffield Teaching Hospitals NHS
NHS Foundation Trust

RISK ASSESSMENT FOR VENOUS THROMBOEMBOLISM (VTE) FOR MEDICAL PATIENTS

Date of admission: ....

Date of assessment: ....

Patients should be risk assessed on admission to hospital, and reassessed whenever their clinical situation changes.

Tick all boxes that apply:

Name:

Date of birth:

Hospital No:

Consultant:

- □ Medical patients with a reduction in mobility or acute illness: → Continue full risk assessment of thrombosis and bleeding risk factors below
- □ Medical patients with **normal mobility and without acute illness** (including cancer patients solely admitted for therapy) → Thromboprophylaxis not usually required. *Risk assessment complete – sign box below*
- □ Medical patients already on therapeutic anticoagulation (e.g. heparin, warfarin, dabigatran, rivaroxaban, apixaban e.t.c) *with no intention to change* → Continue existing anticoagulation *Risk assessment complete – sign box below*

Thrombosis (VTE) Risk Factors

Patient related risk factors

- □ Active cancer or cancer treatment
- □ Age over 60 yrs
- □ Dehydration
- □ One or more significant comorbidities (e.g. heart disease; metabolic, endocrine or respiratory pathologies)
- □ Acute infection or inflammatory condition
- □ Personal history or first-degree relative with a history of VTE
- □ Obesity (BMI greater than 30kg/m^2)
- □ Use of oestrogen-containing contraceptive therapy
- □ Use of hormone replacement therapy (HRT)
- □ Varicose veins with phlebitis
- □ Known thrombophilia
- □ Pregnant or less than 6 weeks post partum – complete *antenatal (PD7262)* or *postnatal (PD7263) risk assessment for venous thromboembolism in pregnancy* form as appropriate

Admission related risk factors

- □ Significantly reduced mobility for 3 days or more (relative to normal state)
- □ Hip or knee replacement surgery
- □ Hip fracture
- □ Total anaesthetic plus surgical time greater than 90mins
- □ Surgery involving pelvis or lower limb with a total anaesthetic plus surgical time greater than 60mins
- □ Acute surgical admission with inflammatory or intra-abdominal condition
- □ Critical care admission
- □ Surgery with significant reduction in mobility

Bleeding Risk Factors

Patient related risk factors

- □ Active bleeding
- □ Acquired bleeding
- □ Uncontrolled hypertension (greater than or equal to 230mmHg systolic or 120mmHg diastolic)
- □ Acute stroke
- □ Thrombocytopaenia (platelets less than 75 x10^9/L)
- □ Untreated inherited bleeding disorders (e.g. haemophilia and von Willebrand's disease)

Admission related risk factors

- □ Neurosurgery, spinal surgery or eye surgery
- □ Other procedure with high bleeding risk
- □ Lumbar puncture/ epidural/ spinal anaesthesia expected within the next 12 hours
- □ Lumbar puncture/ epidural/ spinal anaesthesia within the previous 4 hours

Action taken (for full advice see "STH Guidelines for the prevention of Venous Thromboembolic Disease")

- □ No thrombosis risk factors present so no prophylaxis needed
- □ Thrombosis risk factors present but no prophylaxis prescribed: *state reason why (e.g. already on anticoagulation therapy, bleeding risk outweighs thrombosis risk)*...

Thrombosis risk factors present: □ dalteparin prescribed
□ mechanical prophylaxis prescribed (if dalteparin contraindicated)

If thrombosis risk factors are present, prescribe dalteparin UNLESS bleeding risk outweighs VTE risk, or dalteparin is contraindicated; see below for doses:

Patient's weight to nearest kilogram	eGFR greater than or equal to 20 mL/min/1.73m^2	eGFR less than 20 mL/min/1.73m^2	Pregnant women
Less than 45kg	2500 units once daily	2500 units once daily	Refer to *"Risk assessment for VTE in pregnancy and up to 6 weeks postpartum* or *"STH Guidelines for the prevention of Venous Thromboembolic Disease"*
45-99kg	5000 units once daily		
100-149kg	7500 units once daily		
150kg and greater	5000 units twice a day		

8 of 12

Supplementary material: Evaluating routine data sources to determine the incidence of hospital acquired thrombosis and major bleeding in medical and surgical inpatients.

Site 4 – Electronic form within prescribing electronic health record designed to trigger consideration of VTE risk assessment at the point of hospital admission and prompt prescribing in accordance with local guidelines (St Thomas' NHS Foundation Trust).

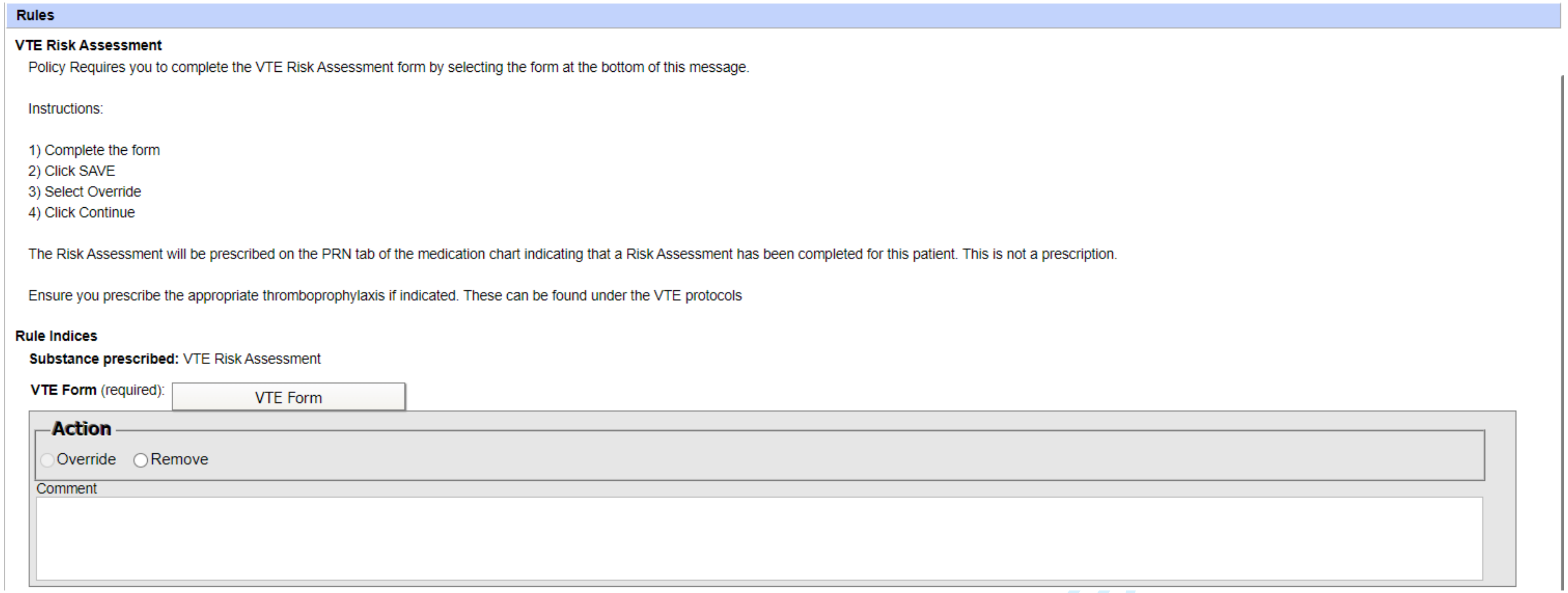

Rules

VTE Risk Assessment

Policy Requires you to complete the VTE Risk Assessment form by selecting the form at the bottom of this message.

Instructions:

1) Complete the form
2) Click SAVE
3) Select Override
4) Click Continue

The Risk Assessment will be prescribed on the PRN tab of the medication chart indicating that a Risk Assessment has been completed for this patient. This is not a prescription.

Ensure you prescribe the appropriate thromboprophylaxis if indicated. These can be found under the VTE protocols

Rule Indices

Substance prescribed: VTE Risk Assessment

VTE Form (required): VTE Form

Action

Override Remove

Comment

Supplementary data

Figure 2: Workflow diagram. CTRU – Clinical Trials Research Unit. VTEAM – Venous Thromboembolism Assessment Models (VTEAM) Study

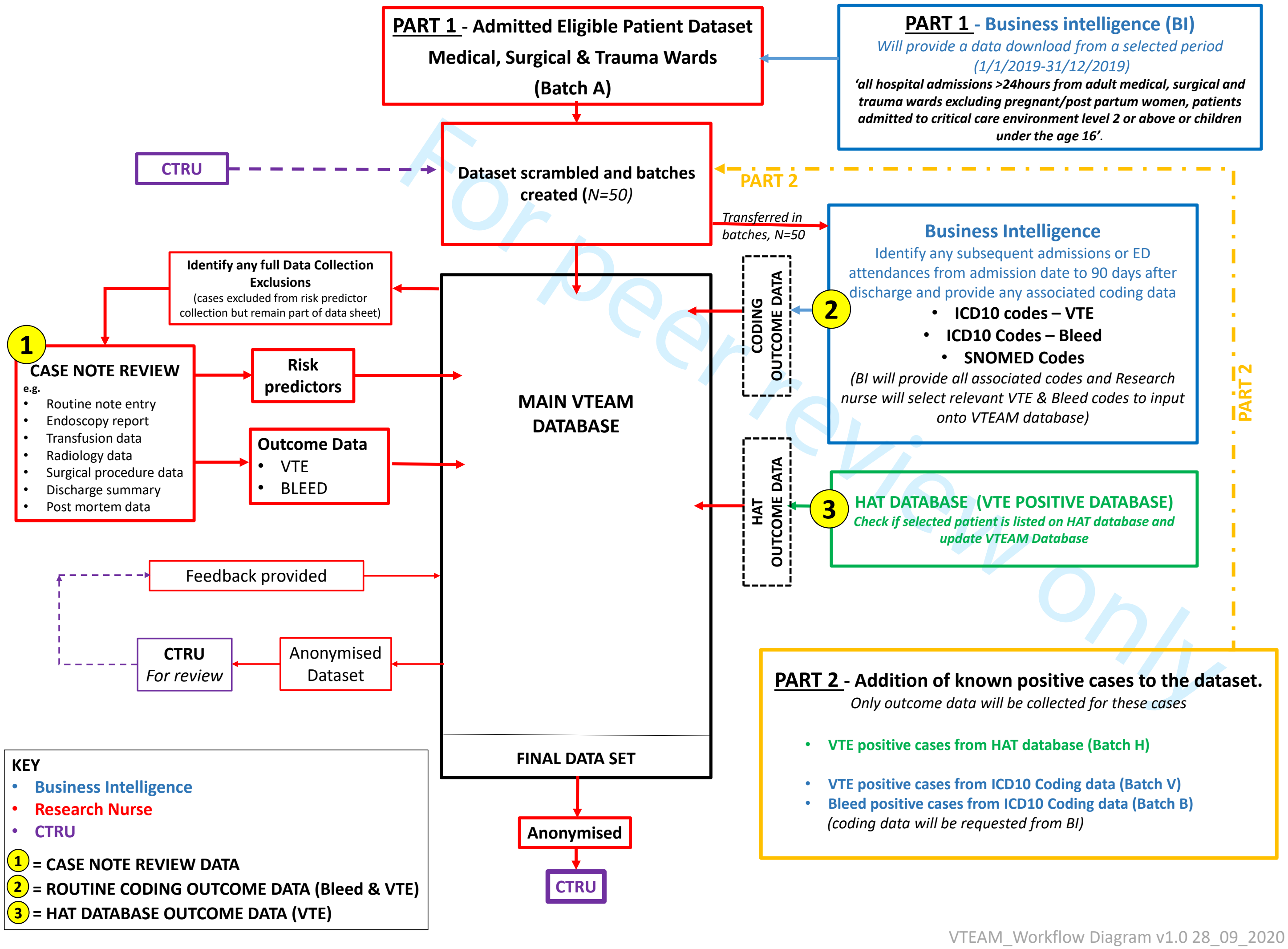

For peer review only

Supplementary data

Table 1: Relevant ICD10 codes for VTE and Bleeding agreed by chief investigators and approved by project management group

ICD10 - 4 digit	3 character description	4 character description	All diagnoses	Main diagnosis	Category	Sub-Category	Final selection
I26.0	Pulmonary embolism	Pulmonary embolism with mention of acute cor pulmonale	4031	2353	VTE		Yes
I26.9	Pulmonary embolism	Pulmonary embolism without mention of acute cor pulmonale	108637	53273	VTE		Yes
I80.1	Phlebitis and thrombophlebitis	Phlebitis and thrombophlebitis of femoral vein	10156	4294	VTE		Yes
I80.2	Phlebitis and thrombophlebitis	Phlebitis and thrombophlebitis of other deep vessels of lower extremities	61647	24297	VTE		Yes
I80.3	Phlebitis and thrombophlebitis	Phlebitis and thrombophlebitis of lower extremities, unspecified	3971	1876	VTE		Yes
I80.9	Phlebitis and thrombophlebitis	Phlebitis and thrombophlebitis of unspecified site	2906	524	VTE		Yes
I82.2	Other venous embolism and thrombosis	Embolism and thrombosis of vena cava	3891	543	VTE		Yes
I82.8	Other venous embolism and thrombosis	Embolism and thrombosis of other specified veins	10001	1870	VTE		Yes
I82.9	Other venous embolism and thrombosis	Embolism and thrombosis of unspecified vein	1124	215	VTE		Yes
I60.0	Subarachnoid haemorrhage	Subarachnoid haemorrhage from carotid siphon and bifurcation	226	212	Bleeding	intracranial bleed	Yes
I60.1	Subarachnoid haemorrhage	Subarachnoid haemorrhage from middle cerebral artery	1125	1014	Bleeding	intracranial bleed	Yes
I60.2	Subarachnoid haemorrhage	Subarachnoid haemorrhage from anterior communicating artery	1731	1599	Bleeding	intracranial bleed	Yes
I60.3	Subarachnoid haemorrhage	Subarachnoid haemorrhage from posterior communicating artery	899	838	Bleeding	intracranial bleed	Yes
I60.4	Subarachnoid haemorrhage	Subarachnoid haemorrhage from basilar artery	384	324	Bleeding	intracranial bleed	Yes
I60.5	Subarachnoid haemorrhage	Subarachnoid haemorrhage from vertebral artery	101	87	Bleeding	intracranial bleed	Yes
I60.6	Subarachnoid haemorrhage	Subarachnoid haemorrhage from other intracranial arteries	564	513	Bleeding	intracranial bleed	Yes

For peer review only

Supplementary data

I60.7	Subarachnoid haemorrhage	Subarachnoid haemorrhage from intracranial artery, unspecified	426	319	Bleeding	intracranial bleed	Yes
I60.8	Subarachnoid haemorrhage	Other subarachnoid haemorrhage	977	737	Bleeding	intracranial bleed	Yes
I60.9	Subarachnoid haemorrhage	Subarachnoid haemorrhage, unspecified	7642	4585	Bleeding	intracranial bleed	Yes
I61.0	Intracerebral haemorrhage	Intracerebral haemorrhage in hemisphere, subcortical	4996	4396	Bleeding	intracranial bleed	Yes
I61.1	Intracerebral haemorrhage	Intracerebral haemorrhage in hemisphere, cortical	5439	4254	Bleeding	intracranial bleed	Yes
I61.2	Intracerebral haemorrhage	Intracerebral haemorrhage in hemisphere, unspecified	1431	1157	Bleeding	intracranial bleed	Yes
I61.3	Intracerebral haemorrhage	Intracerebral haemorrhage in brain stem	1029	866	Bleeding	intracranial bleed	Yes
I61.4	Intracerebral haemorrhage	Intracerebral haemorrhage in cerebellum	1901	1508	Bleeding	intracranial bleed	Yes
I61.5	Intracerebral haemorrhage	Intracerebral haemorrhage, intraventricular	3678	1886	Bleeding	intracranial bleed	Yes
I61.6	Intracerebral haemorrhage	Intracerebral haemorrhage, multiple localized	764	561	Bleeding	intracranial bleed	Yes
I61.8	Intracerebral haemorrhage	Other intracerebral haemorrhage	3854	3103	Bleeding	intracranial bleed	Yes
I61.9	Intracerebral haemorrhage	Intracerebral haemorrhage, unspecified	11863	9028	Bleeding	intracranial bleed	Yes
I62.0	Other nontraumatic intracranial haemorrhage	Subdural haemorrhage (acute)(nontraumatic)	17161	8197	Bleeding	intracranial bleed	Yes
I62.1	Other nontraumatic intracranial haemorrhage	Nontraumatic extradural haemorrhage	318	100	Bleeding	intracranial bleed	Yes
I62.9	Other nontraumatic intracranial haemorrhage	Intracranial haemorrhage (nontraumatic), unspecified	3230	2383	Bleeding	intracranial bleed	Yes
I85.0	Oesophageal varices	Oesophageal varices with bleeding	4074	2876	Bleeding	gastrointestinal	Yes
K22.6	Other diseases of oesophagus	Gastro-oesophageal laceration-haemorrhage syndrome	7232	3237	Bleeding		Yes
K25.0	Gastric ulcer	Gastric ulcer - Acute with haemorrhage	2077	1469	Bleeding	gastrointestinal	Yes
K25.2	Gastric ulcer	Gastric ulcer - Acute with both haemorrhage and perforation	49	20	Bleeding	gastrointestinal	Yes

For peer review only

Supplementary data

K25.4	Gastric ulcer	Gastric ulcer - Chronic or unspecified with haemorrhage	4742	2951	Bleeding	gastrointestinal	Yes
K25.6	Gastric ulcer	Gastric ulcer - Chronic or unspecified with both haemorrhage and perforation	145	74	Bleeding	gastrointestinal	Yes
K26.0	Duodenal ulcer	Duodenal ulcer - Acute with haemorrhage	2955	2161	Bleeding	gastrointestinal	Yes
K26.2	Duodenal ulcer	Duodenal ulcer - Acute with both haemorrhage and perforation	126	96	Bleeding	gastrointestinal	Yes
K26.4	Duodenal ulcer	Duodenal ulcer - Chronic or unspecified with haemorrhage	7607	4972	Bleeding	gastrointestinal	Yes
K26.6	Duodenal ulcer	Duodenal ulcer - Chronic or unspecified with both haemorrhage and perforation	386	263	Bleeding	gastrointestinal	Yes
K27.0	Peptic ulcer, site unspecified	Peptic ulcer, site unspecified - Acute with haemorrhage	78	32	Bleeding	gastrointestinal	Yes
K27.2	Peptic ulcer, site unspecified	Peptic ulcer, site unspecified - Acute with both haemorrhage and perforation	4	1	Bleeding	gastrointestinal	Yes
K27.4	Peptic ulcer, site unspecified	Peptic ulcer, site unspecified - Chronic or unspecified with haemorrhage	231	116	Bleeding	gastrointestinal	Yes
K27.6	Peptic ulcer, site unspecified	Peptic ulcer, site unspecified - Chronic or unspecified with both haemorrhage and perforation	29	9	Bleeding	gastrointestinal	Yes
K28.0	Gastrojejunal ulcer	Gastrojejunal ulcer - Acute with haemorrhage	29	24	Bleeding	gastrointestinal	Yes
K28.2	Gastrojejunal ulcer	Gastrojejunal ulcer - Acute with both haemorrhage and perforation	3	3	Bleeding	gastrointestinal	Yes
K28.4	Gastrojejunal ulcer	Gastrojejunal ulcer - Chronic or unspecified with haemorrhage	149	96	Bleeding	gastrointestinal	Yes
K28.6	Gastrojejunal ulcer	Gastrojejunal ulcer - Chronic or unspecified with both haemorrhage and perforation	11	6	Bleeding	gastrointestinal	Yes
K29.0	Gastritis and duodenitis	Acute haemorrhagic gastritis	3340	1365	Bleeding	gastrointestinal	Yes
K62.5	Other diseases of anus and rectum	Haemorrhage of anus and rectum	37545	21106	Bleeding	gastrointestinal	Yes
K66.1	Other disorders of peritoneum	Haemoperitoneum	3317	642	Bleeding	gastrointestinal	Yes
K92.0	Other diseases of digestive system	Haematemesis	67589	27503	Bleeding	gastrointestinal	Yes
K92.1	Other diseases of digestive system	Melaena	67036	22979	Bleeding	gastrointestinal	Yes
K92.2	Other diseases of digestive system	Gastrointestinal haemorrhage, unspecified	192053	97428	Bleeding	gastrointestinal	Yes

For peer review only

Supplementary data

M25.0	Other joint disorders, not elsewhere classified	Haemarthrosis	3362	1730	Bleeding		Yes
N02.0	Recurrent and persistent haematuria	Recurrent and persistent haematuria - Minor glomerular abnormality	48	21	Bleeding		Yes
N02.1	Recurrent and persistent haematuria	Recurrent and persistent haematuria - Focal and segmental glomerular lesions	286	76	Bleeding		Yes
N02.2	Recurrent and persistent haematuria	Recurrent and persistent haematuria - Diffuse membranous glomerulonephritis	1858	517	Bleeding		Yes
N02.3	Recurrent and persistent haematuria	Recurrent and persistent haematuria - Diffuse mesangial proliferative glomerulonephritis	160	49	Bleeding		Yes
N02.4	Recurrent and persistent haematuria	Recurrent and persistent haematuria - Diffuse endocapillary proliferative glomerulonephritis	10	4	Bleeding		Yes
N02.5	Recurrent and persistent haematuria	Recurrent and persistent haematuria - Diffuse mesangiocapillary glomerulonephritis	47	12	Bleeding		Yes
N02.6	Recurrent and persistent haematuria	Recurrent and persistent haematuria - Dense deposit disease	9	1	Bleeding		Yes
N02.7	Recurrent and persistent haematuria	Recurrent and persistent haematuria - Diffuse crescentic glomerulonephritis	164	48	Bleeding		Yes
N02.8	Recurrent and persistent haematuria	Recurrent and persistent haematuria - Other	10852	1289	Bleeding		Yes
N02.9	Recurrent and persistent haematuria	Recurrent and persistent haematuria - Unspecified	2902	1331	Bleeding		Yes
N93.8	Other abnormal uterine and vaginal bleeding	Other specified abnormal uterine and vaginal bleeding	4801	2899	Bleeding	other bleed	Yes
N93.9	Other abnormal uterine and vaginal bleeding	Abnormal uterine and vaginal bleeding, unspecified	24423	11036	Bleeding	other bleed	Yes
R04.0	Haemorrhage from respiratory passages	Epistaxis	48741	24610	Bleeding	other bleed	Yes
R04.1	Haemorrhage from respiratory passages	Haemorrhage from throat	191	69	Bleeding	other bleed	Yes
R04.2	Haemorrhage from respiratory passages	Haemoptysis	32143	12743	Bleeding	other bleed	Yes
R04.8	Haemorrhage from respiratory passages	Haemorrhage from other sites in respiratory passages	1332	222	Bleeding	other bleed	Yes
R04.9	Haemorrhage from respiratory passages	Haemorrhage from respiratory passages, unspecified	83	23	Bleeding	other bleed	Yes

For peer review only

Supplementary data

R23.3	Other skin changes	Spontaneous ecchymoses	10624	2774	Bleeding		Yes
R58.X	Haemorrhage, not elsewhere classified	Haemorrhage, not elsewhere classified	2747	408	Bleeding		Yes
T81.0	Complications of procedures, not elsewhere classified	Haemorrhage and haematoma complicating a procedure, not elsewhere classified	67338	28601	Bleeding		Yes
T81.7	Complications of procedures, not elsewhere classified	Vascular complications following a procedure, not elsewhere classified	1000	201	Bleeding		Yes

For peer review only

STROBE Statement—Checklist of items that should be included in reports of ***cohort studies***

	Item No	Recommendation	Page No
**Title and abstract**	1	(*a*) Indicate the study's design with a commonly used term in the title or the abstract	1
		(*b*) Provide in the abstract an informative and balanced summary of what was done and what was found	3
**Introduction**			
Background/rationale	2	Explain the scientific background and rationale for the investigation being reported	5
Objectives	3	State specific objectives, including any prespecified hypotheses	5
**Methods**			
Study design	4	Present key elements of study design early in the paper	6-7
Setting	5	Describe the setting, locations, and relevant dates, including periods of recruitment, exposure, follow-up, and data collection	6-7
Participants	6	(*a*) Give the eligibility criteria, and the sources and methods of selection of participants. Describe methods of follow-up	6-7
		(*b*) For matched studies, give matching criteria and number of exposed and unexposed	
Variables	7	Clearly define all outcomes, exposures, predictors, potential confounders, and effect modifiers. Give diagnostic criteria, if applicable	8
Data sources/ measurement	8*	For each variable of interest, give sources of data and details of methods of assessment (measurement). Describe comparability of assessment methods if there is more than one group	7-8
Bias	9	Describe any efforts to address potential sources of bias	8
Study size	10	Explain how the study size was arrived at	8-9
Quantitative variables	11	Explain how quantitative variables were handled in the analyses. If applicable, describe which groupings were chosen and why	8
Statistical methods	12	(*a*) Describe all statistical methods, including those used to control for confounding	8
		(*b*) Describe any methods used to examine subgroups and interactions	
		(*c*) Explain how missing data were addressed	
		(*d*) If applicable, explain how loss to follow-up was addressed	
		(*e*) Describe any sensitivity analyses	
**Results**			
Participants	13*	(a) Report numbers of individuals at each stage of study—eg numbers potentially eligible, examined for eligibility, confirmed eligible, included in the study, completing follow-up, and analysed	11
		(b) Give reasons for non-participation at each stage	
		(c) Consider use of a flow diagram	
Descriptive data	14*	(a) Give characteristics of study participants (eg demographic, clinical, social) and information on exposures and potential confounders	Table 1&2, 11
		(b) Indicate number of participants with missing data for each variable of interest	
		(c) Summarise follow-up time (eg, average and total amount)	
Outcome data	15*	Report numbers of outcome events or summary measures over time	11

For peer review only

Main results	16	(*a*) Give unadjusted estimates and, if applicable, confounder-adjusted estimates and their precision (eg, 95% confidence interval). Make clear which confounders were adjusted for and why they were included<br>(*b*) Report category boundaries when continuous variables were categorized<br>(*c*) If relevant, consider translating estimates of relative risk into absolute risk for a meaningful time period	11
Other analyses	17	Report other analyses done—eg analyses of subgroups and interactions, and sensitivity analyses	12
**Discussion**			
Key results	18	Summarise key results with reference to study objectives	13
Limitations	19	Discuss limitations of the study, taking into account sources of potential bias or imprecision. Discuss both direction and magnitude of any potential bias	13-14
Interpretation	20	Give a cautious overall interpretation of results considering objectives, limitations, multiplicity of analyses, results from similar studies, and other relevant evidence	15-16
Generalisability	21	Discuss the generalisability (external validity) of the study results	16
**Other information**			
Funding	22	Give the source of funding and the role of the funders for the present study and, if applicable, for the original study on which the present article is based	2

*Give information separately for exposed and unexposed groups.

Note: An Explanation and Elaboration article discusses each checklist item and gives methodological background and published examples of transparent reporting. The STROBE checklist is best used in conjunction with this article (freely available on the Web sites of PLoS Medicine at http://www.plosmedicine.org/, Annals of Internal Medicine at http://www.annals.org/, and Epidemiology at http://www.epidem.com/). Information on the STROBE Initiative is available at http://www.strobe-statement.org.

For peer review only

For peer review only - http://bmjopen.bmj.com/site/about/guidelines.xhtml
